# Explainable deep learning improves human mental models of self-driving cars

**DOI:** 10.1038/s41586-026-10950-5

**Published:** 2026-09-02

**Authors:** Eoin M. Kenny, Akshay Dharmavaram, Sang Uk Lee, Tung Phan-Minh, Shreyas Rajesh, Yunqing Hu, Laura Major, Momchil S. Tomov, Julie A. Shah

**Affiliations:** 1 Computer Science and Artificial Intelligence Laboratory (CSAIL), Massachusetts Institute of Technology, Cambridge, MA USA; 2 Motional AD Inc., Boston, MA USA; 3 Department of Psychology and Center for Brain Science, Harvard University, Cambridge, MA USA; 4 Department of Aeronautics and Astronautics, Massachusetts Institute of Technology, Cambridge, MA USA

**Keywords:** Computer science, Computational science

## Abstract

Self-driving cars increasingly rely on deep neural networks to achieve human-like driving^1–3^. The opacity of these black-box planners makes it challenging to accurately anticipate when they will fail^4–6^, with potentially catastrophic consequences^7–9^. Although research into interpreting these systems has surged, most of it is confined to simulations or toy setups because of the difficulty of real-world deployment^10,11^, leaving the practical utility of these techniques unknown. Here, we introduce the Concept-Wrapper Network (CW-Net), a method for faithfully explaining the behaviour of machine-learning-based planners that causally grounds their reasoning in human-interpretable concepts without sacrificing performance. We deploy CW-Net on a real self-driving car and show that the resulting explanations improve the human driver’s mental model of the vehicle, allowing them to better predict its behaviour, particularly in surprising situations. This demonstrates that explainable deep learning integrated into self-driving cars can be both understandable and useful in a realistic deployment setting. We anticipate our method could be applied to other safety-critical systems, such as autonomous drones and robotic surgeons, as well as to other architectures, such as end-to-end learning systems and vision–language–action models. Overall, our study establishes a deployment-validated pathway to interpretability for autonomous agents, which could help make them more transparent and safe.

## Abstract (web summary)

Concept-Wrapper Network, a concept-based explanation approach deployed on a real autonomous vehicle, improves drivers’ ability to understand and predict its behaviour.

## Main

There are hundreds of companies developing autonomous vehicle (AV) technology globally^12^, promising to revolutionize transportation for everyone. At present, the industry spans two segments: fully autonomous ride-hail systems and consumer vehicles with driver assistance^13^. In consumer vehicles, machine learning (ML) solutions have markedly improved the technology, yet they still require human intervention in unusual or challenging situations in which learned planners may not determine the correct action^14^. As driving is safety-critical, these infrequent failures matter, making it essential that the human driver is able to anticipate and be prepared for these situations^15^. However, the opaque nature of ML planners makes it challenging to interpret and communicate the causes of their decisions, hampering the ability of human drivers to understand and predict AV behaviour while achieving real-time situational awareness^16–18^.

Lack of effective communication between the AV and the human driver has contributed to multiple high-profile incidents, some resulting in fatalities^7–9^, highlighting the urgent need to make ML planners interpretable^11^. Previous studies have sought to address this using surveys and simulated scenarios^19–26^, a human driver emulating the AV^27,28^, or language models providing rationales for the driving policy in natural language^3,29^. However, these studies were theoretical, did not provide causally faithful explanations, were only evaluated in simulation or did not convincingly show the practical utility of the explanations to end users. This leaves open the question of how to provide explanations that are understandable, useful and faithful to the decision-making process of the AV in a realistic setting.

To answer this question, we scale up our work on interpretable-by-design deep reinforcement learning^10^ using motifs from the literature on concept-bottleneck models^30^ to propose the Concept-Wrapper Network (CW-Net). CW-Net grounds the reasoning of a black-box ML planner in human-interpretable concepts, such as ‘Approaching stopped vehicle’ or ‘Close to cyclist’. This method is rooted in case-based reasoning, a classical artificial intelligence (AI) approach^31–34^ inspired by cognitive models of human reasoning and memory^35^. CW-Net can be applied to arbitrary pretrained deep neural networks, does not require retraining from scratch and does not degrade the performance of the original black-box ML planner. As the inferred concepts are the sole input to the final decision-making module of CW-Net and therefore directly determine AV behaviour, we refer to them as causally faithful. Notably, our approach contrasts with popular post hoc explanation methods^36,37^, which are applied to pretrained models and do not, by construction, guarantee faithfulness to the model decision process^38^.

We apply CW-Net to an ML planner trained to imitate human driving behaviour using inverse reinforcement learning^1,2^. We replace the final (reward) layer of the pretrained deep neural network with a concept classifier, followed by a new reward layer. We then jointly train the classifier and the new reward layer to predict scenario types and driving decisions, respectively, without modifying the rest of the network. Evaluation on a large-scale benchmark^39^ confirms that CW-Net is able to classify concepts without compromising driving behaviour. To study the utility of the explanations, we deploy CW-Net on a real self-driving car with a safety driver in a semi-naturalistic study^40^. We demonstrate three situations in which the driver’s initially inaccurate mental model is subsequently improved by the CW-Net explanations, which in turn allows the driver to more accurately predict AV behaviour. We then observe similar mental model improvement in larger online studies simulating these scenarios. Last, we link these results to more complex real-world situations by deploying CW-Net on public roads in Las Vegas. We use these new data in a large online study (*n* = 100) to demonstrate that improvements in mental model goodness lead to improved predictive ability and situational awareness. Overall, our work demonstrates how explainable deep learning can help users of an advanced autonomous system improve their mental model of the system and better anticipate its future behaviour.

## Black-box planner

**Fig. 1:**
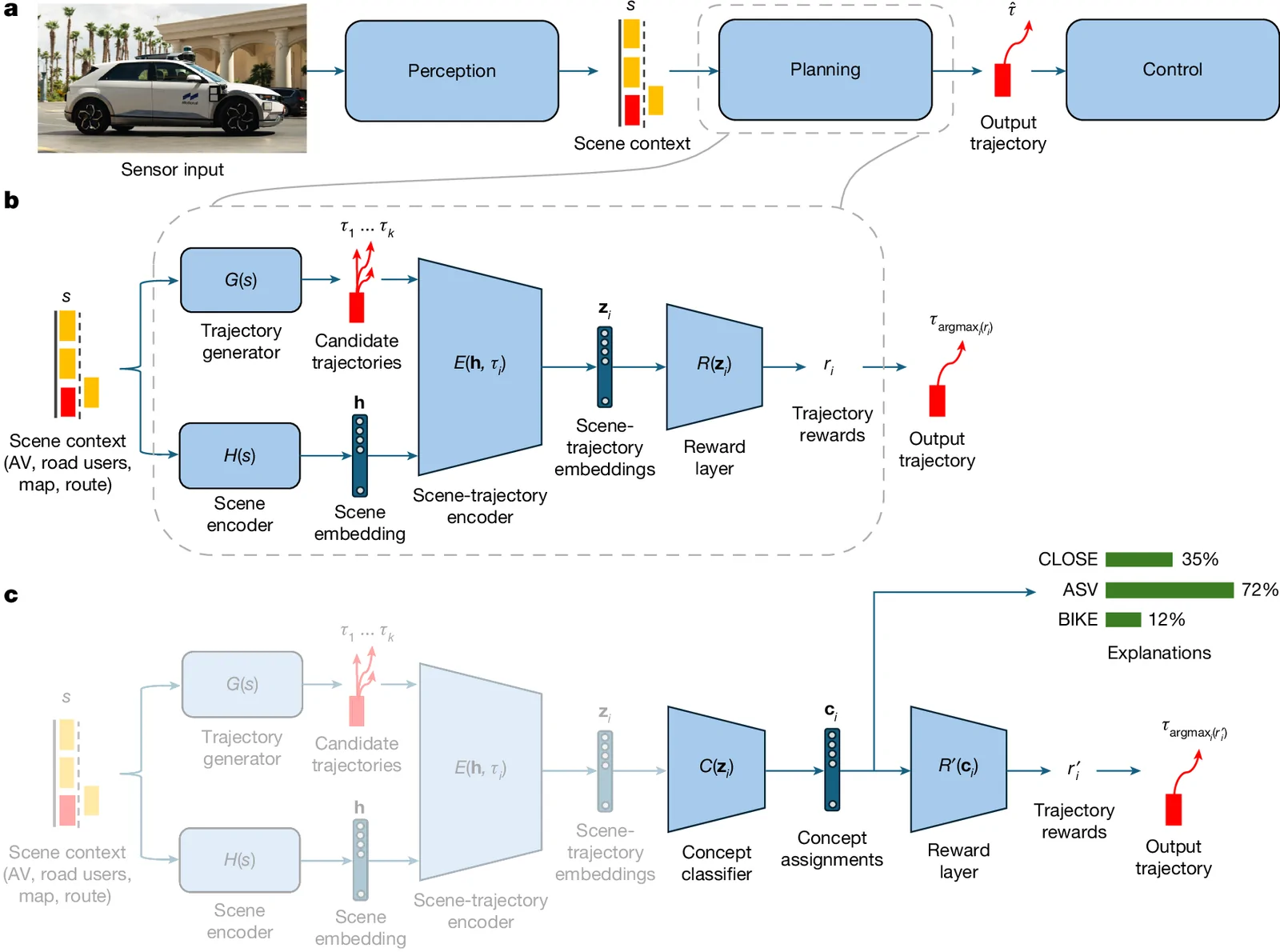
Planner architecture. **a**, AV stack. Sensory input is processed by the perception module to generate scene context *s*. The planning module processes *s* to compute trajectory $\widehat{\tau }$, which is followed by the control module. **b**, Black-box ML planner. Here, *s* is fed to trajectory generator *G*, which produces candidate trajectories {*τ*_1_, …, *τ*_*k*_}, and scene encoder *H*, which produces a scene embedding **h**. These are fed into encoder *E*, which produces scene-trajectory embeddings **z**_*i*_ for each *τ*_*i*_, which are in turn fed into the reward layer *R*, which computes a reward *r*_*i*_ for each trajectory. The model is trained to output higher rewards for trajectories closer to the ground-truth human trajectories. **c**, CW-Net. Identical to **b**, except **z**_*i*_ is fed to a concept classifier *C*, which produces concept assignments **c**_*i*_. These are fed to a new reward layer *R*′ to produce rewards ${r}_{i}^{{\prime} }$. In parallel, **c**_*i*_ is processed to generate the explanations. The reward layer is trained to prefer the same trajectories as the black-box ML planner, whereas the concept layer is supervised with ground-truth scenario labels. The weights of the faded components are frozen during training. CLOSE, Close to another vehicle; ASV, Approaching stopped vehicle; BIKE, Close to cyclist. Adapted with permission from ref. ^1^, IEEE.

We focus on the planning module of the AV stack (Fig. 1a), which takes as input a scene context *s* and outputs a trajectory $\widehat{\tau }$. Here, *s* is a symbolic object-oriented representation of the scene, computed by the perception module, and $\widehat{\tau }$ is the trajectory that the subsequent controller module should follow. We use a deep neural network architecture^1,2^ consisting of a scene encoder *H*(*s*) → **h** and a trajectory generator *G*(*s*) → {*τ*_1_, …, *τ*_*k*_}, followed by a scene-trajectory encoder *E*(**h**, *τ*_*i*_) → **z**_*i*_ and a final reward layer *R*(**z**_*i*_) → *r*_*i*_ (Fig. 1b). *H* computes a scene embedding **h** and *G* computes a set of *k* candidate trajectories {*τ*_1_, …*, τ*_*k*_}. Those are combined in *E* to compute an embedding **z**_*i*_ for each trajectory *τ*_*i*_. Finally, *R* computes an estimated reward *r*_*i*_ for each trajectory *τ*_*i*_, quantifying how human-like it is. In other words, *r*_*i*_ is higher when *τ*_*i*_ is more similar to how a human would drive in this situation.

During inference, the trajectory with the highest reward is selected on each iteration:

$$\widehat{\tau }=\mathop{\mathrm{arg}\,\mathrm{max}\,}\limits_{\tau \in \{{\tau }_{1}\ldots {\tau }_{k}\}}R(E({\bf{h}},\tau ))$$

We use inverse reinforcement learning to train the planner on 80 h of human expert driving^1,2^.

## Planning over human-friendly concepts

To make the ML planner interpretable, we modify the planner architecture to additionally provide explanations for its behaviour^10^. Our working definition of explainable AI follows ref. ^41^, who define it as ‘AI systems that can explain their rationale to a human user, characterize their strengths and weaknesses, and convey an understanding of how they will behave in the future’.

Specifically, we replace *R* with a concept classifier *C*(**z**_*i*_) → **c**_*i*_, followed by a new reward layer ${R}^{{\prime} }({{\bf{c}}}_{i})\to {r}_{i}^{{\prime} }$ (Fig. 1c). The concept classifier *C* computes a logit vector **c**_*i*_ that is passed through a softmax and/or sigmoid layer that, in turn, assigns probabilities to different human-interpretable concepts. As ${R}^{{\prime} }$ computes trajectory rewards from **c**_*i*_, the final decisions are based solely on these concept assignments; hence, they constitute a causally faithful explanation. The rest of the network remains the same.

Similarly to the black-box planner, trajectories are selected according to

$$\hat{\tau }=\mathop{\text{arg max}\,}\limits_{\tau \in \{{\tau }_{1}...{\tau }_{k}\}}{R}^{{\prime} }(C(E({\bf{h}},\tau )))$$

**Extended Data Fig. 1:**
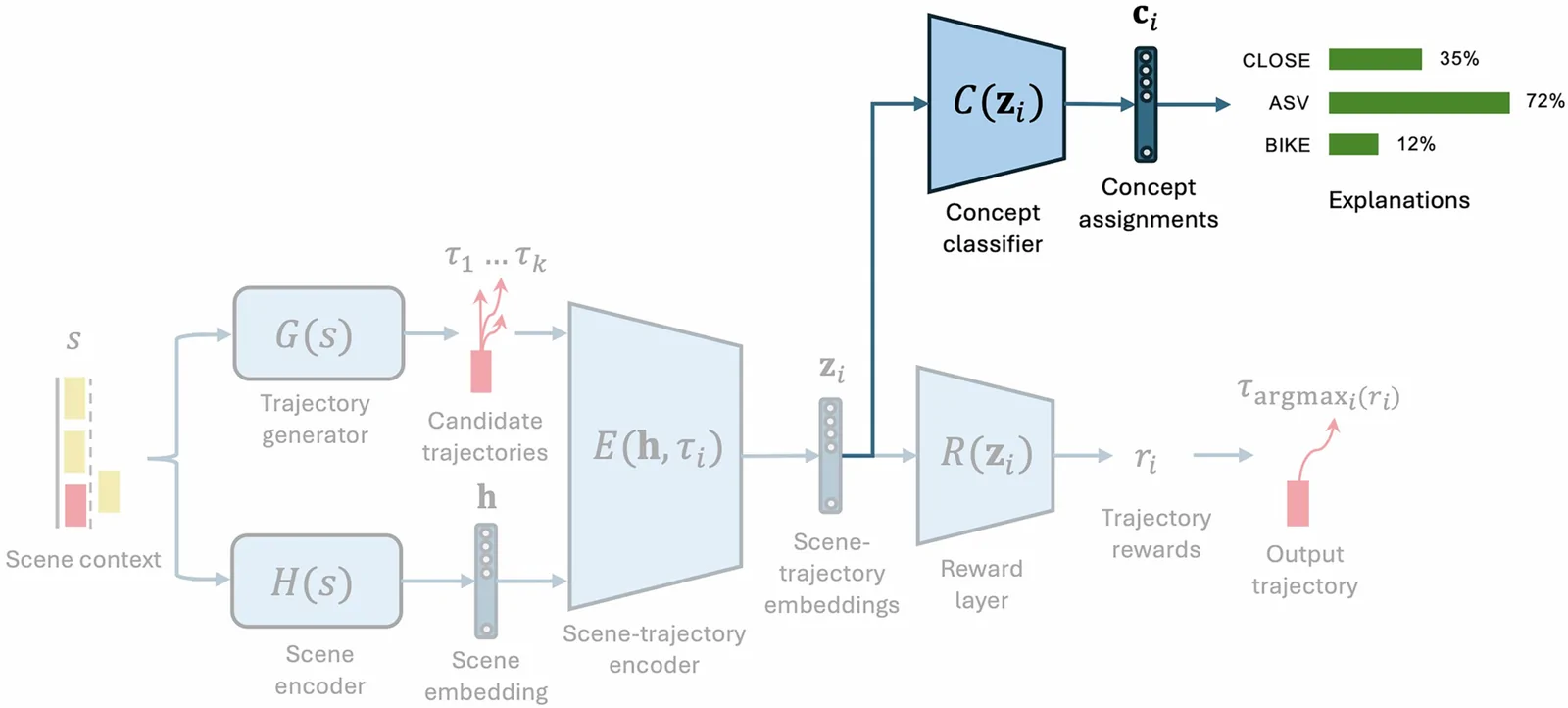
Parallel architecture. This is identical to the black-box planner (Fig. 1b; greyed out here), except the scene-trajectory embeddings are fed to the concept classifier *C* in parallel to the (original) reward model *R*. The black-box part of the architecture (greyed out) is kept frozen. These concept classifications are then converted to probabilities (x100 to convert to percentages) and presented to the user. This approach is analogous to probing techniques in the mechanistic interpretability literature^56^.

We train CW-Net to jointly predict concept labels and mimic the driving decisions of the black-box planner. Specifically, **c**_*i*_ is supervised with labels corresponding to types of scenario, such as ‘Approaching stopped vehicle’ or ‘Close to cyclist’ (see Methods, ‘Concept details’ for a full list of concepts). This ensures that CW-Net assigns a unique interpretable concept to each unit in **c**_*i*_. At the same time, ${r}_{i}^{{\prime} }$ is supervised with trajectories selected by the black-box planner using a cross-entropy loss. During training, the rest of the deep neural network (*H*, *G* and *E*) is kept frozen (see Methods, ‘CW-Net training’ for more details). We focus our study on this particular ML planner architecture (but do consider alternatives; see Extended Data Fig. 1), noting that previous work indicates CW-Net would generalize effectively to other architectures^10^.

## Classifying concepts

As a baseline, we first evaluated the black-box planner (without CW-Net) using closed-loop simulations with the nuPlan simulator on the nuPlan dataset^39^ (Extended Data Table 1). Overall, the results were competitive with the top submissions to the nuPlan challenge, although performance was slightly lacking when starting from a stop (see Methods, ‘Simulation results’). This suggests that there is room for improvement and, importantly, opportunities to study explanations of undesirable behaviour.

We then evaluated the driving performance of CW-Net wrapped around the black-box planner (Extended Data Table 1). The results were equivalent, with less than 1% difference across all metrics, confirming that our method did not degrade driving performance. We also evaluated concept classification on held-out datasets (Supplementary Tables 1 and 2). Mean accuracy was 54%, with 23% precision, 77% recall and an F1 score of 0.31 (see Methods, ‘Simulation results’). Overall, these results indicate that CW-Net can be used to ground the decision making of high-performance ML planners in human-interpretable concepts, without sacrificing driving performance.

## Mental model improvement in deployment

Our central hypothesis is that the explanations from CW-Net would improve the human driver’s mental model of the AV and, by extension, their situational awareness. This would be particularly salient in surprising situations, which is when explanations are most useful^42^. Specifically, the explanations should improve the driver’s ability to understand and address the reasons for AV failures and also to predict its future behaviour.

**Fig. 2:**
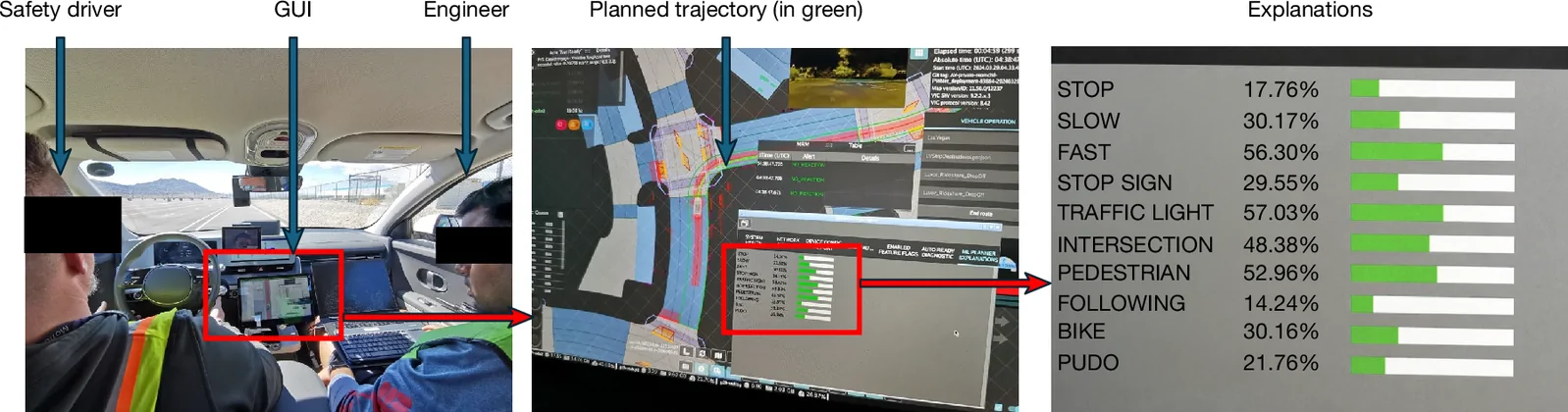
Deployment setup. A safety driver, a support engineer and a researcher were present. The safety driver drove the AV manually between road tests, engaged self-driving mode at the start of each test, monitored AV performance during the test and took over in case of unsafe driving. The support engineer deployed CW-Net and set scenario destinations. The researcher directed testing. The dashboard included a map with overlaid object detections (*s*) from the perception module and the output trajectory ($\widehat{\tau }$). Explanations ${{\bf{c}}}_{\widehat{i}}$ from CW-Net were shown as percentages for easier interpretation^50^. PUDO, pedestrian pickup and drop-off.

To test this hypothesis, we deploy CW-Net on a real AV on a private track using the Lab2Car wrapper^40^ and observe how safety drivers react to surprising events and the corresponding CW-Net explanations (Fig. 2). These situations were not planned, but instead occurred naturally, with minimal intervention from the researchers, who dictated only high-level plans for each day. This semi-naturalistic study design allowed us to assess the utility of the explanations in naturally occurring surprising situations. To measure AV predictability, we record the drivers’ ability to make counterfactual predictions about AV behaviour in these situations. To note the drivers’ mental models, together with their predictive ability, we additionally record their think-aloud thoughts before and after considering CW-Net explanations^43^. We focus on concepts that relate to other road users and can be easily tested counterfactually (see Methods, ‘Concept details’).

### Unexpected stopping for nearby vehicles

**Fig. 3:**
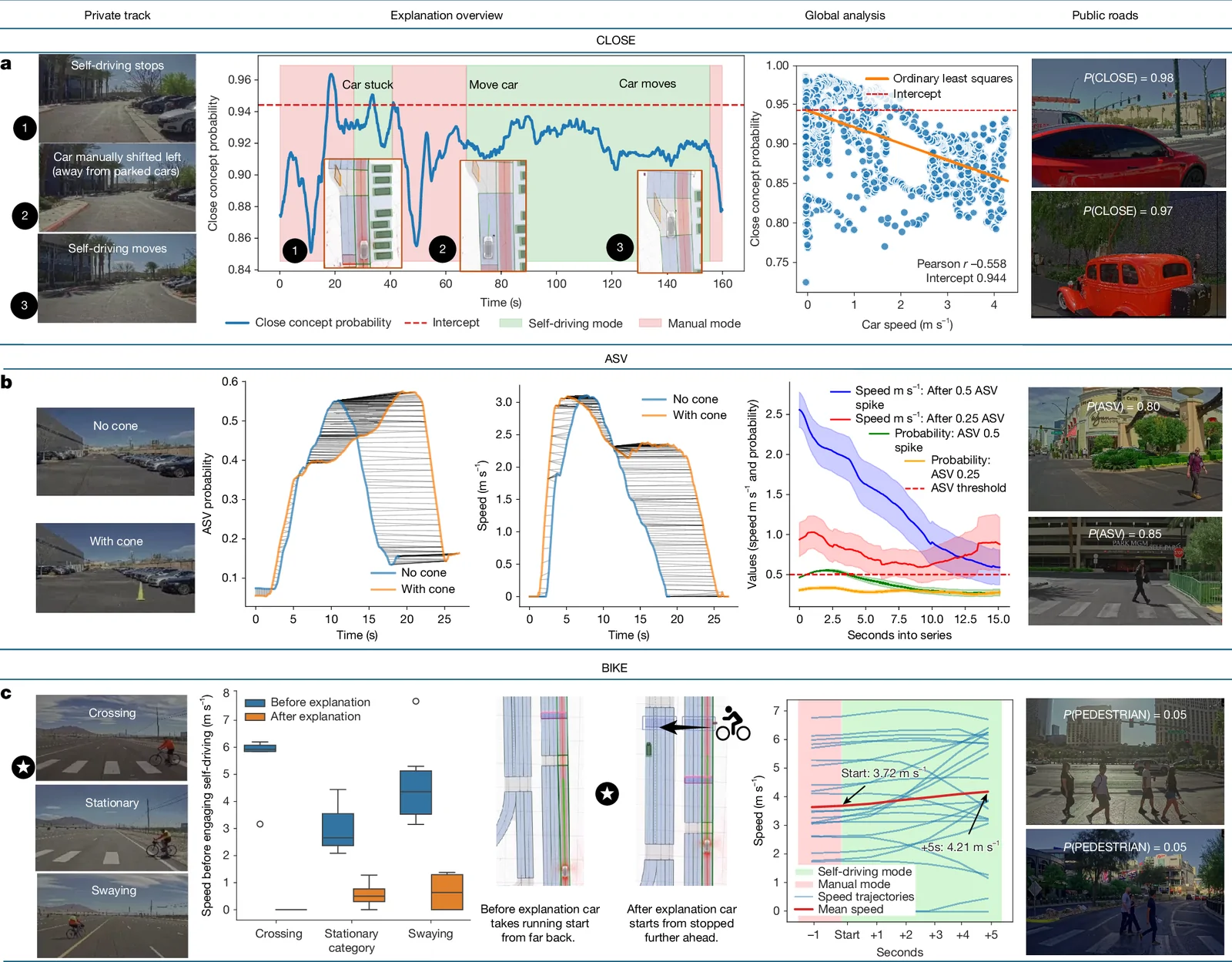
Results. **a**, The CLOSE concept activated when the car got stuck next to parked vehicles. The driver initially thought the pickup–drop-off area was the cause, but the explanation suggested it was the parked vehicles. When the driver engaged self-driving away from the parked vehicles, activation of the CLOSE concept decreased, and the AV moved again, counter to their initial mental model and consistent with the explanations. Across tests, CLOSE correlated with speed and the intercept accurately predicted this event (*n* = 5,545; ordinary least squares). **b**, ASV activated when the car stopped next to a traffic cone. The driver thought the cone was the cause, but the explanation suggested the AV was hallucinating a stopped vehicle. When we removed the cone, the same phantom braking and concept activation occurred. Across all deployment data, ASV correlated with reductions in speed when spiking above 0.5 probability (*n* = 50; s.e.m. shown). **c**, BIKE failed to activate in our first round of tests with a cyclist, but the car always stopped safely for the cyclist. After observing the explanation, the driver engaged self-driving from slower speeds in a second round of tests (*n *= 23). Follow-up analyses showed that the AV stopped for the cyclist because of backup safety mechanisms unrelated to CW-Net, which indicated that the driver’s increased level of caution was appropriate. (**a**–**c**), Right column, scenarios from public-road deployment, analogous to those observed on the private track. We used the PEDESTRIAN concept instead of BIKE as it behaved similarly and pedestrians were more frequently encountered on public roads. Likewise, we used pedestrians in place of the cone in our ASV tests as there were no cones encountered on public roads. The s.e.m. and 3-s rolling averages are shown in relevant plots. Source Data

We observed that the AV repeatedly came to a stop shortly before a pedestrian pickup–drop-off zone (Fig. 3a). The driver’s intuition was that the car stopped because of the pickup–drop-off zone, but the explanations indicated that the planner stopped because it detected that it was ‘Close to another vehicle’ (the CLOSE concept). To test this hypothesis, the driver manually moved the car farther from the parked cars. At this point, the probability of CLOSE decreased and the AV began moving again, thus supporting the alternative hypothesis. A full timeline of events is detailed in Fig. 3a. We fitted the intercept of the CLOSE probability against the speed of the AV globally and found it accurately predicts stopping and starting for this event.

### Hallucinating a stopped vehicle ahead

At another location, the AV would reliably come to a stop next to a traffic cone (Fig. 3b). The driver’s initial mental model was that the cone was responsible for the phantom brake. However, the ‘Approaching stopped vehicle’ (ASV) concept peaked shortly before the car stopped. This suggested an alternative hypothesis that the planner matched the current situation with training scenarios labelled ASV, which in turn promotes stopping behaviour associated with these scenarios. As a counterfactual test, the cone was removed. The AV exhibited the same stopping behaviour at the same location, along with similar ASV probability and speed profiles (*L*_2_ similarities of 7.37 and 1.6 between the respective time-warped profiles, compared with an average *L*_2_ > 200 for random events), thus supporting the alternative hypothesis. Note that although there was no vehicle in front of the AV, the explanation is causally faithful to the underlying planner and explains why it stopped (namely, because it incorrectly detected a stopped vehicle). Figure 3b shows a global analysis of ASV, showing it to be a powerful predictor of braking.

### Reacting safely to cyclist

Finally, we tested the ability of the AV to stop safely for cyclists (Fig. 3c). For each test, the driver engaged self-driving mode while approaching a cyclist. The driver was instructed to engage self-driving from a speed at which they felt safe, because this determines the subsequent speed of the AV. During the initial tests, the AV reliably stopped for the cyclist. However, the BIKE concept maintained a low probability throughout each test (<1%), indicating that CW-Net was failing to detect the cyclist. Over time, the driver became aware of the concept reading and gradually increased their caution by initiating self-driving from slower speeds. A post hoc analysis showed that although the perception system detected the cyclist, the ML planner was not configured to consume inputs for cyclists. As a result, it chose unsafe trajectories that would have collided with the cyclist. In reality, the AV stopped because of a built-in safety backup system, which commands a brake if collision is imminent. This indicates that the increased caution dictated by the driver’s updated mental model was warranted.

## Mental model improvement in simulation

**Extended Data Fig. 2:**
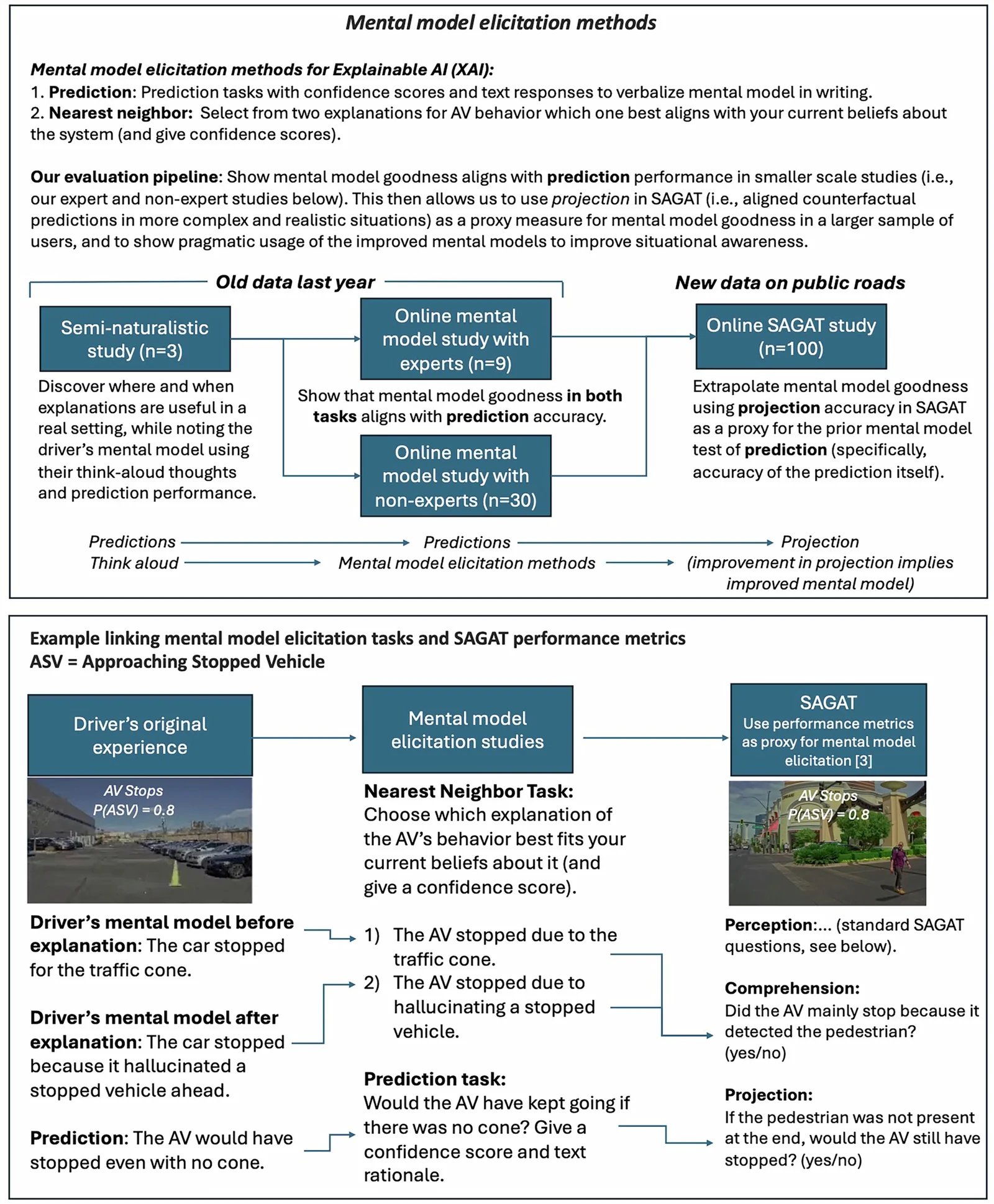
Study methodology and pipeline example. **a**, **Overall pipeline**. First we collected a series of surprising situations expert safety drivers encountered during their day-to-day job testing the AV where the explanations proved to be useful to understand why the AV made certain decisions and how it would behave in alternative scenarios. During these situations, we noted the driver’s mental model through their think-aloud thoughts and behaviour. A mental model elicitation study was run with more expert drivers and non-experts online to more robustly verify these findings and whether mental model goodness did indeed correlate with counterfactual predictive performance. Then, in a large-scale SAGAT study, we used *projection* as a surrogate metric for mental model quality, since it perfectly matches the prior prediction tasks. **b**, **Worked example**. ASV concept.

**Extended Data Fig. 3:**
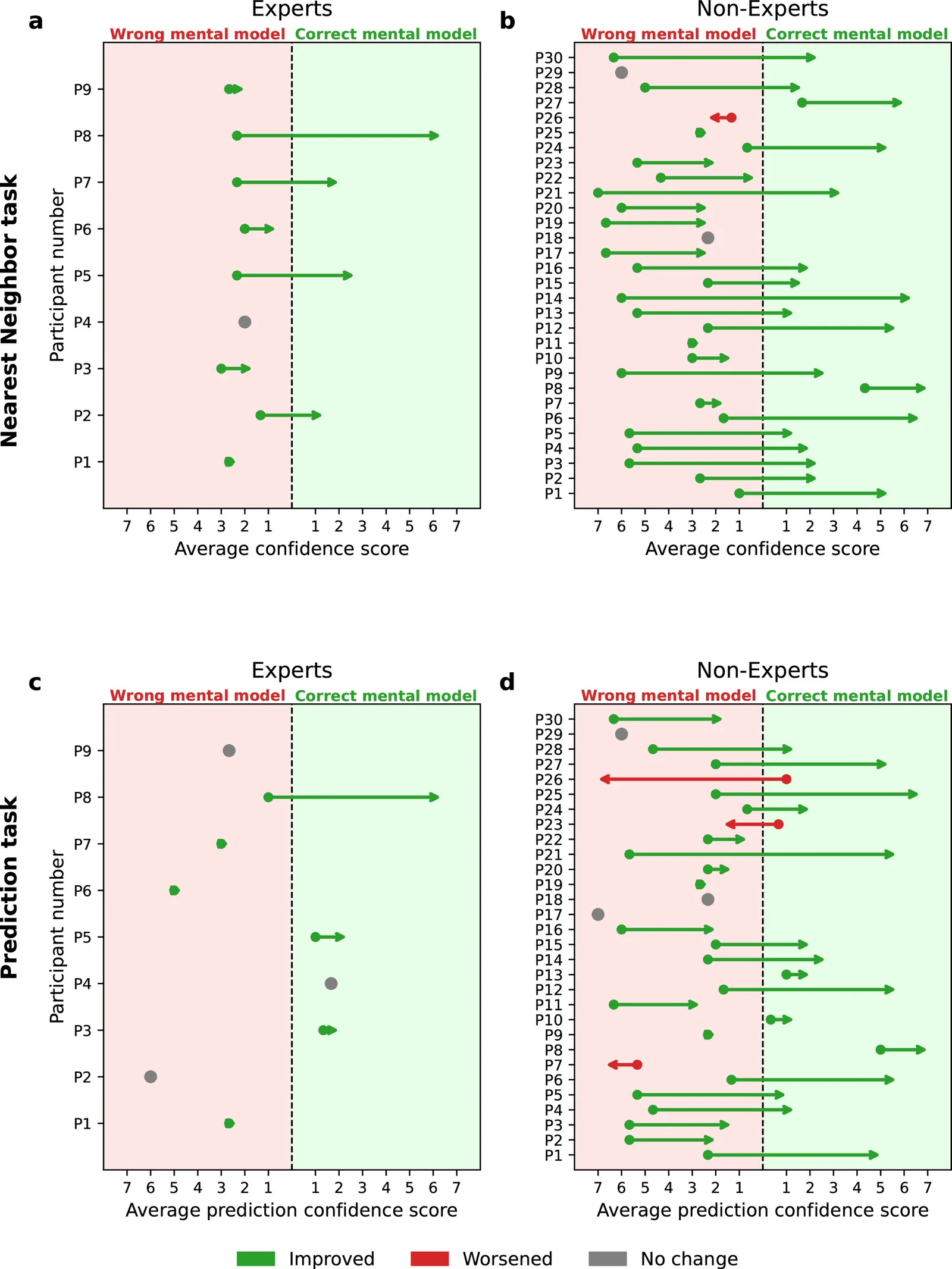
Belief updates in response to explanations on the nearest-neighbor and prediction tasks. **a**–**d**, Mental model improvement on the nearest-neighbor task (**a**, **b**) and performance improvement on the prediction task (**c**, **d**), for experts (*N* = 27; **a**, **c**) and non-experts (*N* = 90; **b**, **d**). Arrows originate at the participants’ initial accuracy/confidence (before observing the explanation) and terminate at their final accuracy/confidence (after observing the explanation). For each participant, initial and final accuracy/confidence are averaged across all 3 scenarios. Green arrows indicate improvement in choice accuracy/confidence, red arrows indicate worsening, and gray points represent participants with no change in beliefs. Source Data

**Extended Data Fig. 4:**
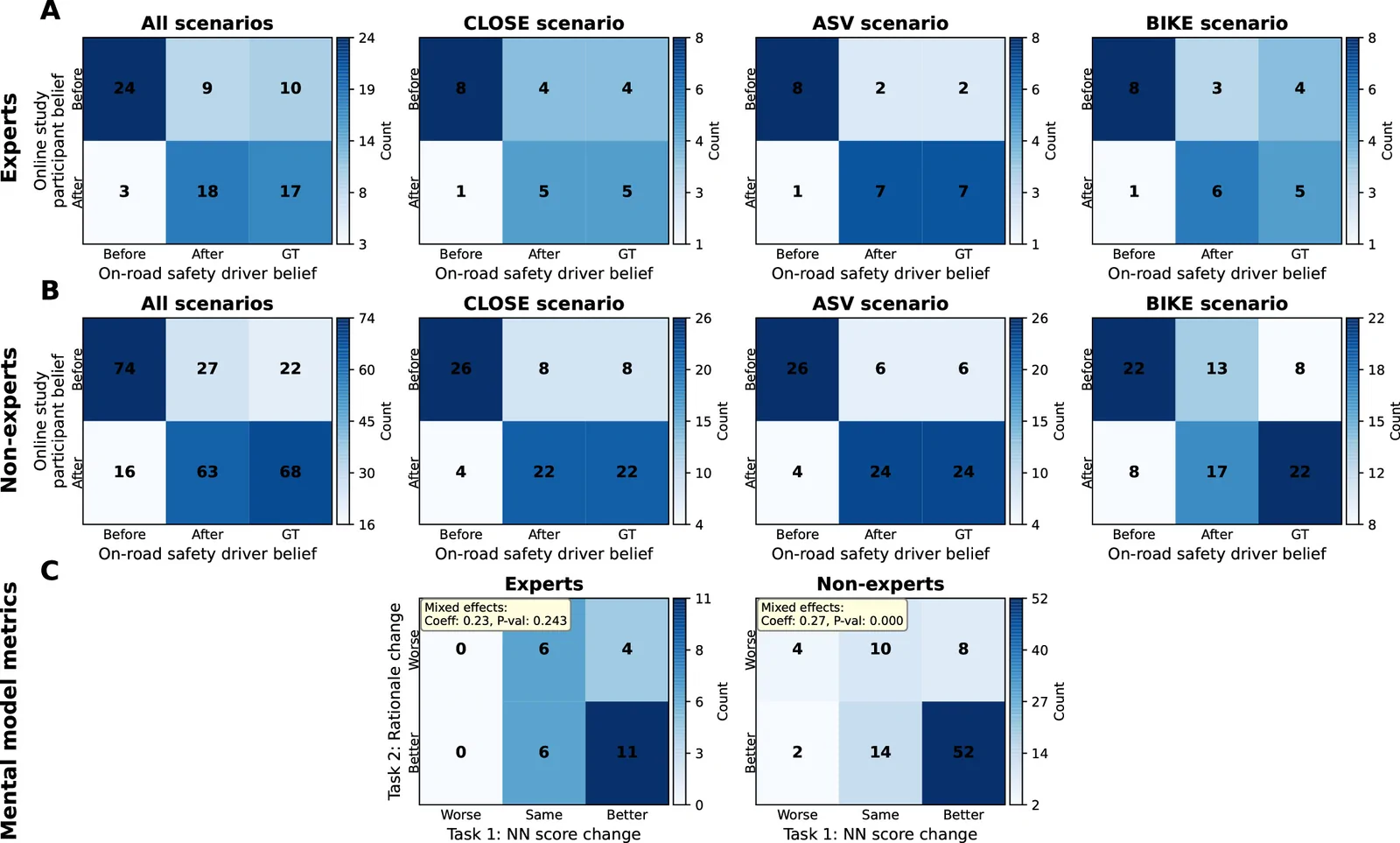
Mental model improvement on free-form text rationale. **A**,**B**, For *N* = 9 experts (**A**) and *N* = 30 non-experts (**B**). Confusion matrices with participant beliefs (mental model and predictions) before/after observing the explanation, extracted from free-form responses in the mental model elicitation study (y-axis), compared to beliefs from the safety drivers from the on-road tests before/after observing the explanation, as well as the ground truth (GT) explanation for AV behavior (x-axis). Numbers indicate raw response counts. **C**. Correlation between mental model metrics across groups. Heatmaps displaying the frequency of alignment between changes in nearest-neighbor (NN) task responses and the free-form text rationale. To determine if the relationship between these two mental model proxies differs by expertise, we performed ordinary least-squares (OLS) regression with clustered standard errors, including an interaction term. The interaction between group (experts vs. non-experts) and score change was not significant (*β* = 0.04, *p* = 0.842), indicating that the relationship between quantitative score improvements and qualitative rationale improvements is largely consistent across both groups. Source Data

**Extended Data Fig. 5:**
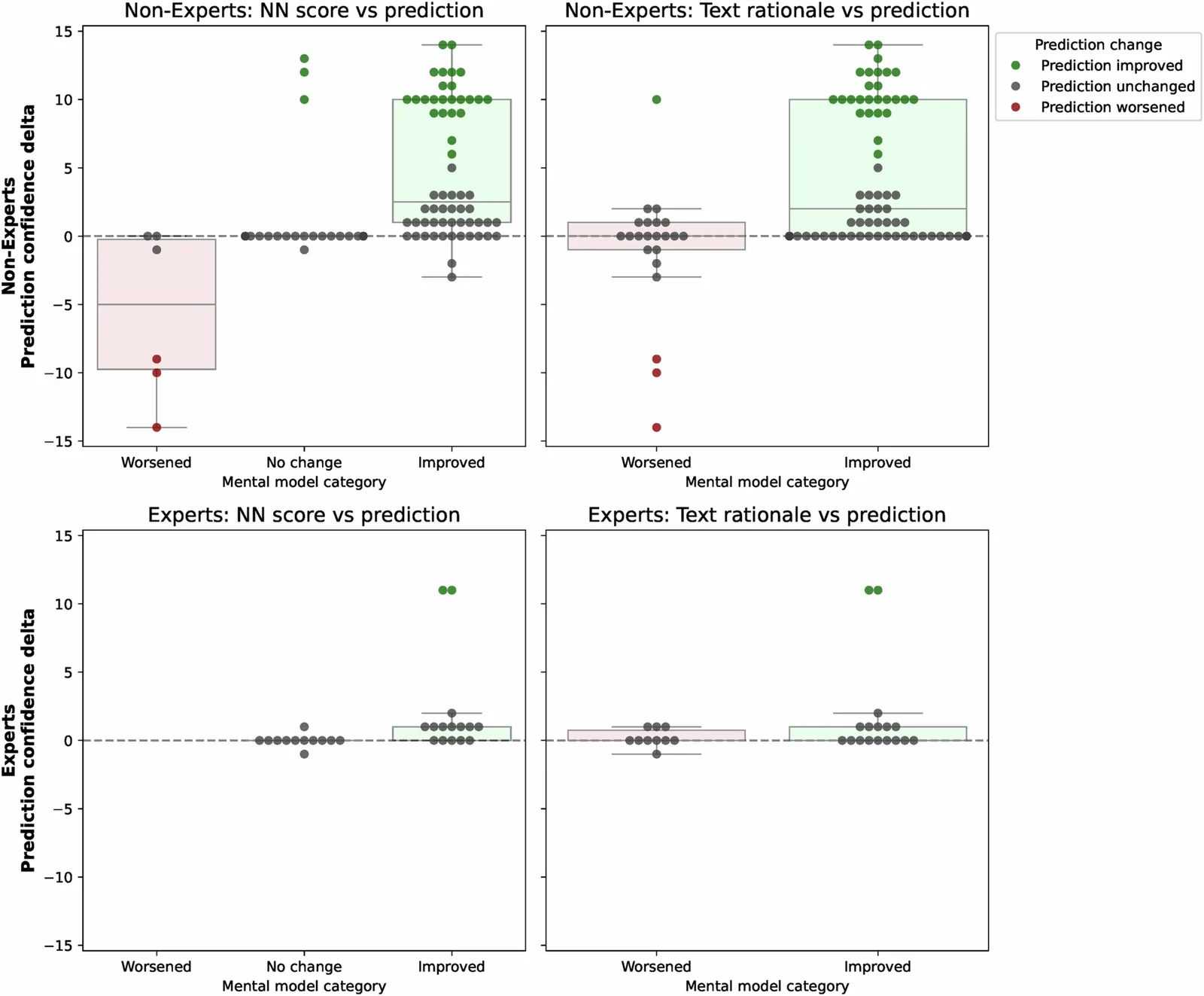
Mental model improvement correlates with prediction improvement. Linear mixed effects (LME) models were used to analyze the relationship between changes in mental model proxies (nearest-neighbor task and free-form text rationale) and prediction accuracy/confidence. Specifically, the y-axis indicates each participant’s Likert confidence after explanation minus before, positive scores indicate they moved towards the correct prediction, and green dots indicate the prediction flipped correctly. For experts (*N* = 9 participants x 3 scenarios), improvement in the nearest-neighbor (NN) task significantly correlates with prediction improvement (*β* = 2.02, *SE* = 0.87, *p* = 0.02). The text rationale shows a similar positive effect (*β* = 1.70, *SE* = 0.91, *p* = 0.06), although it does not reach significance. For non-experts (*N* = 30 participants x 3 scenarios), both NN improvement (*β* = 9.86, *SE* = 2.07, *p* < 0.001) and text rationale improvement (*β* = 5.03, *SE* = 1.23, *p* < 0.001) are significantly correlated with prediction improvement. Positive betas indicate that improving the mental model proxy is associated with a higher increase in prediction accuracy/confidence. Source Data

**Extended Data Fig. 6:**
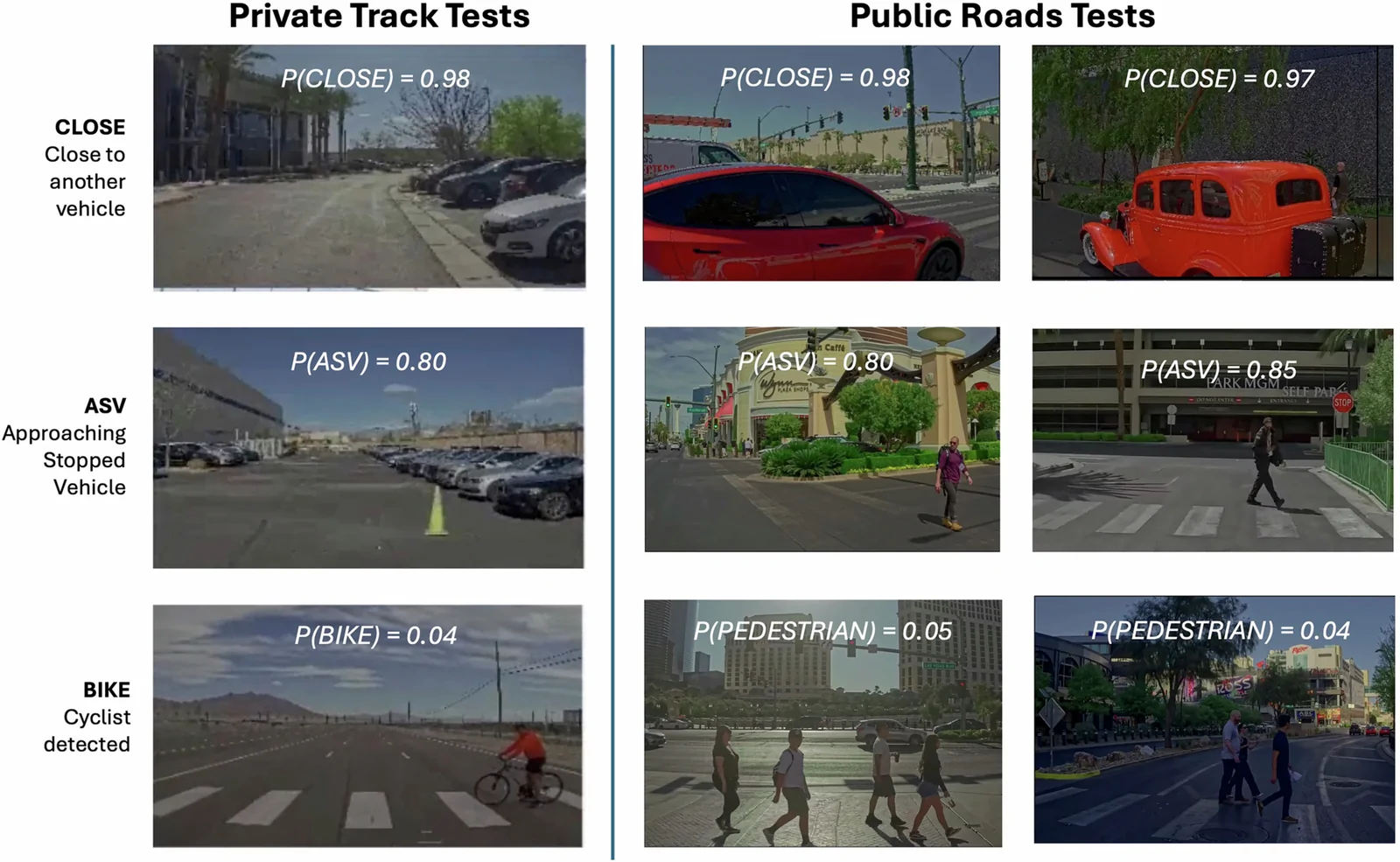
Naturalistic scenario examples. The original surprising scenarios discovered on the private track and the analogous scenarios discovered on public roads.

**Extended Data Fig. 7:**
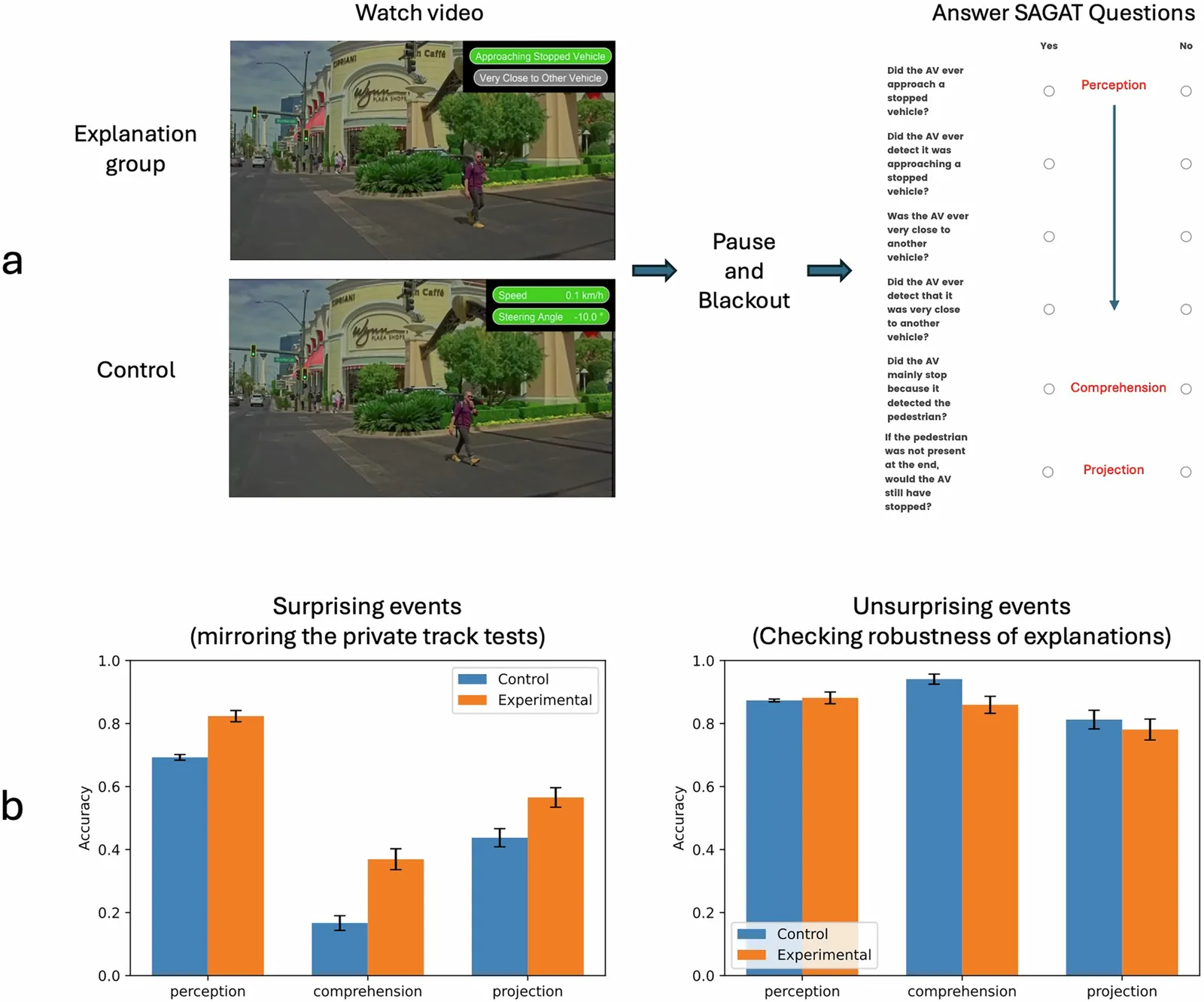
SAGAT materials example and online SAGAT study results. **a:** **SAGAT materials example**. The two conditions (explanation vs. no explanation) were presented with the same video, followed by a ‘blackout’, and then 6 questions related to situational awareness. **b: Online SAGAT study results**. Users in the experimental group reported significantly higher accuracy on perception, comprehension, and projection during surprising events. During unsurprising events, there were no significant differences. Although a negative trend was noted for comprehension in the experimental group, it did not reach significance after Bonferroni correction (see Table S6). Taken with the results of the mental model elicitation studies, which found counterfactual prediction accuracy correlated with mental model goodness, these SAGAT results strongly indicate the improvements in situational awareness were due to underlying improvements in user mental models of the AV (*n* = 99; s.e.m. shown). Source Data

**Extended Data Fig. 8:**
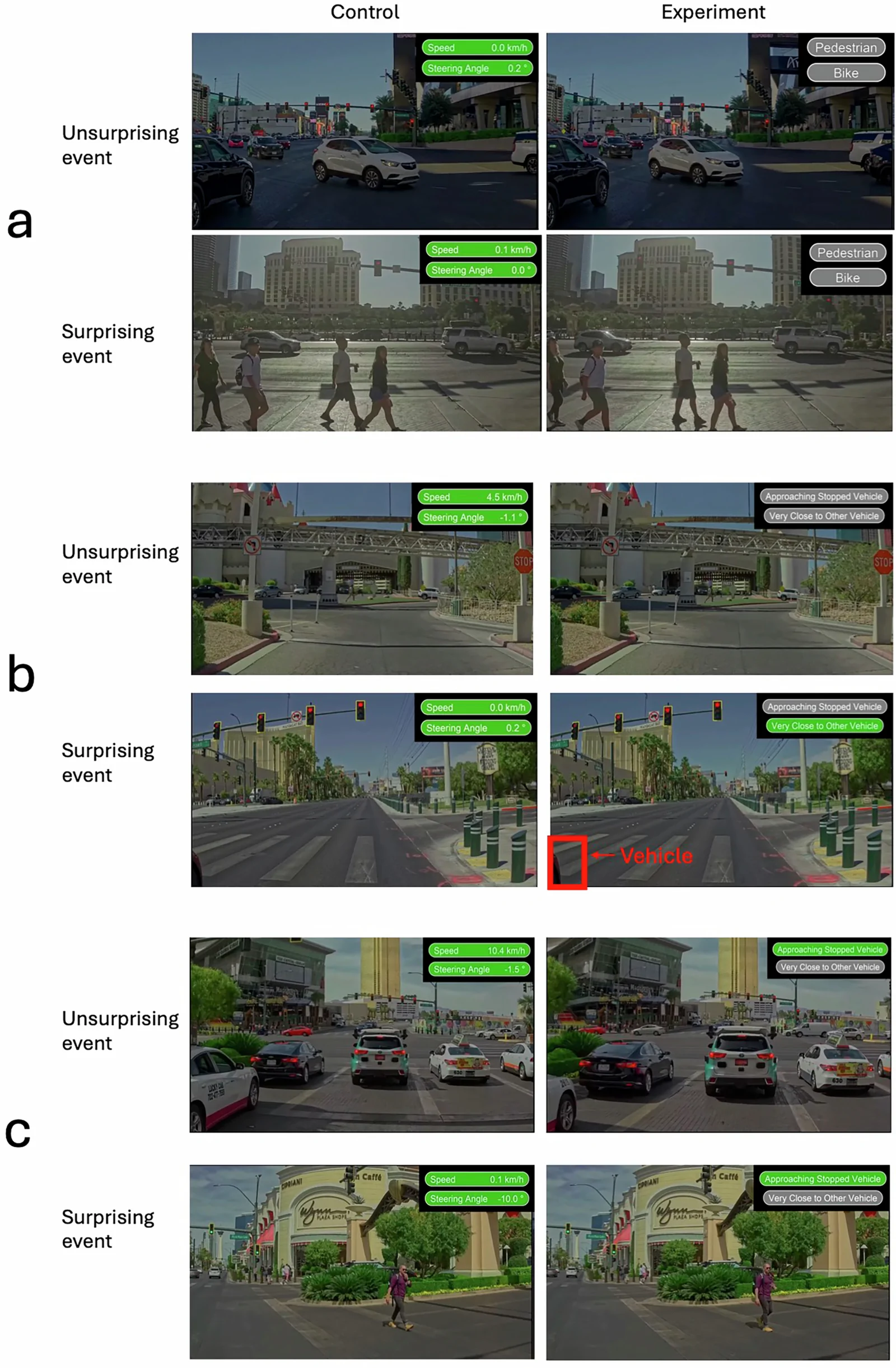
User material examples for each concept. Shown during a surprising and an unsurprising event, with the control condition on the left (speed and steering angle only) and the experiment condition on the right (concept labels overlaid). **a**, PEDESTRIAN concept (which replaced BIKE in our SAGAT study). **b**, CLOSE (very close to other vehicle) concept. **c**, ASV (approaching stopped vehicle) concept.

The semi-naturalistic study above illustrates how CW-Net explanations can improve the human driver’s mental model of the AV in surprising real-world situations, leading to better understanding and predictability of AV behaviour. To validate that these results replicate in larger populations and generalize to naturalistic scenarios, we conduct several larger follow-up studies (Extended Data Figs. 2–8). First, we show that in simulations of the real-world scenarios in the previous section, CW-Net explanations consistently improve mental models and predictions for both experts and non-experts alike. Second, we collect data from CW-Net in naturalistic real-world scenarios on public roads and show that the explanations improve situational awareness—an indirect measure of mental model goodness^44^—in surprising situations, without degrading it in unsurprising situations.

### Mental models and predictability

The purpose of the first study is to (1) demonstrate that the effects observed on the road reproduce in larger populations, and (2) establish a link between direct measures of mental model goodness and performance on downstream tasks—such as predicting AV behaviour—so that the latter can be used as a proxy for the former. We conducted an online survey in which we showed participants replays of the scenarios from the private track (front camera recordings with overlaid CW-Net explanations; see Extended Data Fig. 6).

For each scenario, we asked participants to choose the reason for the AV’s behaviour that best aligns with their current beliefs (that is, ‘Why did the AV do that?’) and to make a counterfactual prediction (that is, ‘What would the AV do if ...?’). Following ref. ^43^, we refer to the former as the nearest-neighbour task—because it forces participants to pick the nearest explanation to their beliefs—and the latter as the prediction task. Both tasks consist of a multiple-choice question followed by a confidence score. The prediction task additionally includes a free-form text response in which participants describe the reasons for their prediction. Both directly probe the participants’ mental models, whereas the prediction task itself also serves as a proxy performance metric for mental model goodness. Importantly, we collected responses before and after observing the explanations. This within-participant design mirrors the experience of the drivers during the real-world tests (Extended Data Figs. 2 and 6). The study was conducted with experts (*n* = 9 Motional drivers and engineers) and non-experts (*n* = 30 pseudo-random participants from Prolific.com).

On the nearest-neighbour task, CW-Net explanations improved mental models for almost all participants (8/9 experts and 27/30 non-experts; Extended Data Fig. 3). Free-form text responses were initially similar to the initial beliefs of the safety drivers during the on-road tests (*P* < 10^−9^) and then shifted towards their final beliefs (*P* < 0.0002) and the ground-truth reasons for AV behaviour (*P* < 10^−5^; exact binomial tests for non-experts; Extended Data Fig. 4a,b). The distribution of mental model updates on both tasks was similar across both groups (*β* = 0.04, *P* = 0.8, ordinary least squares regression; Extended Data Fig. 4c). Importantly, mental model improvement on the nearest-neighbour task correlated with improvements in prediction accuracy (*β* = 2.02 ± 0.87, *P* = 0.02 for experts, *β* = 9.86 ± 2.07, *P* < 0.001 for non-experts; linear mixed-effects models (LMEs); Extended Data Fig. 5, left). A similar effect was observed for the free-form text responses (*β* = 1.70 ± 0.91, *P* = 0.06 for experts, *β* = 5.03 ± 1.23, *P* < 0.001 for non-experts; LMEs; Extended Data Fig. 5, right, and Supplementary Tables 3 and 4). Together, these results support the conclusion that CW-Net explanations can reliably improve mental models of the AV and that prediction performance can serve as a reliable proxy for mental model goodness.

### Explanations and situational awareness

To verify that the effects observed on the private track generalize to more complex, naturalistic scenarios, we deployed CW-Net on public roads in Las Vegas (Extended Data Figs. 6 and 8, and Fig. 3, right panels). Owing to safety reasons and the experimental nature of the ML planner, the safety driver operated the AV in ‘manual’ mode for several hours, with CW-Net running in the background. This allowed us to collect naturalistic scenarios analogous to those encountered on the private track^28,45^ (Extended Data Fig. 6 and Supplementary Table 7). We use replays of those scenarios to conduct a large-scale online study (*n* = 100) within the Situation Awareness Global Assessment Technique (SAGAT) framework tailored for explainable AI^44,46^.

The SAGAT framework is the gold standard for evaluating situational awareness in complex, dynamic tasks^44,46^. In contrast to previous benchmark applications of SAGAT in driving research, which have been conducted almost exclusively within controlled driving-simulator environments^47,48^, we evaluate situational awareness on replays drawn from real-world public-road operation of the AV, addressing long-standing concerns about the ecological validity of simulator-only assessments. It measures the situational awareness of a human operator by freezing a scenario, querying the operator about their perception (that is, ‘What are the inputs to the AV?’), comprehension (that is, ‘Why is the AV doing that?’) and projection (that is, ‘What would the AV do if ...?’) of the situation (Extended Data Fig. 7) and comparing their responses with the ground truth. As counterfactual predictive performance in the previous section aligns with mental model goodness, we use projection in SAGAT as a proxy for mental model elicitation and goodness^43^. For each type of scenario and corresponding concept, we sample two instances in which AV behaviour was surprising (for example, stopping unnecessarily) and two corresponding instances in which it was unsurprising (Supplementary Table 5), to verify that CW-Net did not negatively affect situational awareness. We used a between-participant design in which the experimental group received explanations from CW-Net, whereas the control group received placeholder explanations of AV kinematics to balance cognitive load^49^ (Extended Data Fig. 8).

We found that the CW-Net explanations significantly improved measures of situational awareness for surprising events (Extended Data Fig. 7, left, and Supplementary Table 6, top), with large effect sizes for perception (Cohen’s *d* = 1.290, 95% confidence interval (CI) [0.857, 1.723]; averaged across items, see Supplementary Tables 8 and 9 for internal consistency measures), comprehension (Cohen’s *d* = 0.996, 95% CI [0.578, 1.413]) and a medium effect size for projection (Cohen’s *d* = 0.606, 95% CI [0.203, 1.009]). Conversely, explanations did not affect situational awareness for unsurprising events after Bonferroni correction (Extended Data Fig. 7 and Supplementary Table 6). Together, these results show that the explanations from CW-Net are robust across various conditions and can reliably improve mental models in surprising situations, without significant negative effects in unsurprising situations. Moreover, they demonstrate pragmatic usage of the improved mental models.

## Conclusion

Our work shows how explainable deep learning can provide useful explanations for AVs in a real-world setting. CW-Net achieves this by grounding the reasoning of a pretrained black-box ML planner in human-interpretable concepts that are directly used to make driving decisions. By showing otherwise inaccessible information about the decision-making process of the AV in real time, CW-Net helps improve the human driver’s mental model of the AV. This, in turn, improves the driver’s situational awareness and reveals limitations of the robotic system, helping the driver better anticipate its mistakes. Although we showcase CW-Net using a particular kind of classification-based ML planner architecture, the core idea can be similarly applied to other architectures, including end-to-end learning systems and vision–language–action models. Moreover, although our experimental setup assumes a human driver observing the explanations in real time—an application more suited to advanced driver-assistance systems—CW-Net could equally be applied to debugging and improving fully autonomous AVs. Critically, whereas previous work on explanations and situational awareness for AV decision making has largely been confined to simulated or controlled scenarios^19–26,46^, our deployment of CW-Net on public roads extends these findings to the environmental complexity of real-world driving, providing evidence of ecological validity and practical relevance for the AV industry.

Many systems involving human–robot interaction require real-time explanations, including AI wingmen, drone navigation systems and robotic surgeons. Similarly to AVs, many of these applications increasingly rely on deep learning, with a long tail of potentially catastrophic failure cases. Many regulatory bodies have already made explainable AI a core component of their legislation, with AVs likely to follow suit as they are widely deployed with various users^11^. As such, the success of CW-Net suggests that similar algorithms may prove essential for meeting the regulatory standards for deploying AVs, while building appropriate trust in the technology. In future work, it would be prudent to extend CW-Net to a larger set of concepts—perhaps in an unsupervised manner to overcome the challenges of labelling—and better cover the vast array of concepts relevant to AV settings.

## Methods

### Architecture

The black-box ML planner uses a modified version of the DriveIRL architecture^1,2^ (Fig. 1b). For the trajectory generator *G*, we use a heuristic generator that produces 143 jerk-optimal trajectories to anchor waypoints along the route. For the scene encoder *H*, we use the hierarchical vector transformer (HiVT)^51^ pretrained for multi-agent motion prediction. Apart from the scene embedding **h**, this produces an additional 3 trajectories for the AV, for a total of *k* = 146 candidate trajectories. In the scene-trajectory encoder *E*, trajectories are encoded using a recurrent neural network and then fed jointly with the scene embedding into a transformer layer, which produces the scene-trajectory embeddings **z**_*i*_. The reward model *R* is a multilayer perceptron (MLP). In CW-Net (Fig. 1c), the classifier *C* and the new reward model ${R}^{{\prime} }$ are MLPs.

We avoided testing other methods apart from CW-Net because, at the time of writing, we are unaware of other works that are capable of modelling interpretable-by-design IRL systems. Moreover, we broadly seek a general comparison of concept-based explanations (that is, CW-Net) against no explanation (that is, our control) to help generalizability of the results across the myriad concept-based explainability techniques in the literature.

### CW-Net training

We used two datasets:

- Dataset 1: 500,000 scenarios and 8 concept labels (Supplementary Table 1)
- Dataset 2: 3,000,000 scenarios and 10 concept labels (Supplementary Table 2)

For a full list of the concept labels and their meanings, see section ‘Concept details’. Each scenario was associated with 146 trajectories, thus giving between 73–438 million training data points for the concept classifier, each with multiple concept labels. Our algorithm assumes CW-Net training has access to the original dataset used to train the black-box ML planner, along with annotated human-understandable concept labels for each of these data points. The annotations can be multi-label, meaning that one datum can be associated with as many concepts as desired or useful. The experiments with CLOSE and ASV concepts used models trained on dataset 1. The experiments with BIKE and PEDESTRIAN concepts used models trained on dataset 2.

During training, the parameters of the trajectory generator *G*, the scene encoder *H* and the scene-trajectory encoder *E* are frozen, and only the concept classifier *C* and the new reward model *R*′ are trainable. Two separate losses are optimized jointly.

First, a concept classification loss ${{\mathcal{L}}}_{\mathrm{concept}}$ is used to train *C* to predict the correct concept label(s). In our setting, this loss combines cross-entropy with binary cross-entropy for different concepts, depending on the semantics of the corresponding scenario types. For example, in dataset 1, we use cross-entropy to model the steering concepts of the car (LEFT, RIGHT and STRAIGHT), and the speed concepts (STOP and SLOW), while also using binary cross-entropy to predict the presence of other concepts such as ASV, INTERSECTION and CLOSE. These losses are then averaged into one:

$${{\mathcal{L}}}_{\mathrm{concept}}=\frac{1}{2k}\mathop{\sum }\limits_{i=1}^{k}\left(\frac{1}{{M}_{\mathrm{CCE}}}\mathop{\sum }\limits_{j=1}^{{M}_{\mathrm{CCE}}}{{\mathcal{L}}}_{\mathrm{CCE}}({c}_{i,j},{\hat{c}}_{i,j})+\frac{1}{{M}_{\mathrm{BCE}}}\mathop{\sum }\limits_{l=1}^{{M}_{\mathrm{BCE}}}{{\mathcal{L}}}_{\mathrm{BCE}}({c}_{i,l},{\hat{c}}_{i,l})\right)$$

where *M*_CCE_ is the number of concepts modelled using categorical cross-entropy (for example, steering and speed), and *M*_BCE_ is the number of concepts modelled using binary cross-entropy (for example, presence of features such as ASV, INTERSECTION and CLOSE). *c*_*i*,*j*_ and ${\widehat{c}}_{i,j}$ represent the true and predicted labels for the *j*th concept under CCE for the *i*th data point, whereas *c*_*i*,*l*_ and ${\widehat{c}}_{i,l}$ represent the true and predicted labels for the *l*th concept under BCE for the *i*th data point. On dataset 2, we take a different approach and model everything, including the speed concepts (STOP, SLOW and FAST), with binary cross-entropy. In general, these parameters can be tuned to fit the task at hand.

Second, a cross-entropy loss ${{\mathcal{L}}}_{\mathrm{trajectory}}$ is used to train the network to predict the correct trajectory, which we define as the original trajectory chosen by the black-box planner. Both losses are averaged:

$${{\mathcal{L}}}_{\mathrm{total}}=\frac{1}{2}({{\mathcal{L}}}_{\mathrm{concept}}+{{\mathcal{L}}}_{\mathrm{trajectory}})$$

A focal loss^52^ is applied to counteract data imbalances, just as in the original DriveIRL planner^1,2^. Computationally, our networks were trained on a large distributed setup using PyTorch Lightning.

### Concept separation

When adding interpretability modules post hoc, as we have, there is the possibility that the network will not have learned to separate the concepts of interest, and thus fail to be able to predict them accurately^53^. We observed this in the experimental prototype we tested (Supplementary Table 2), when certain concepts such as CLOSE and PEDESTRIAN had poor precision and high recall, relatively speaking. There are two important points to note here. First, the better trained and more sophisticated an architecture is, the more it naturally learns to separate an impressive number of concepts in an unsupervised manner^54–56^, so this is unlikely to be an issue for most companies with the flagship models in the future. Second, even if CW-Net has not learned to separate certain concepts (for example, red traffic lights compared with green ones), this could highlight the reason why the AV fails to act appropriately—such as stop compared with go—in a given situation (for example, because there are insufficient traffic light scenarios in the training data). Hence, from an explainability perspective, concepts with low accuracy are often particularly useful, as we demonstrate in the paper.

### Alternative architecture

Alongside our primary causal architecture shown in Fig. 1, we also developed an alternative that gave post hoc justifications for AV behaviour (Extended Data Fig. 1). Specifically, we froze the weights of the pretrained black-box ML planner and trained a concept classifier head *C* to work in parallel to the reward layer *R*. Similarly to the causal architecture (Fig. 1c), *C* used the scene-trajectory embeddings **z**_*i*_ to classify the concepts. This approach is beneficial because of its relative simplicity and accessibility, although the drawback is that it may be less faithful to the model’s reasoning process, as the concept classifications are not directly used by the model to rank state-trajectory pairs. However, there is ample evidence that these explanations are often capable^57^, so we include both as an option and demonstrate their utility. Specifically, this parallel architecture was used in the real-world scenarios with the CLOSE concept (section ‘Unexpected stopping for nearby vehicles’).

### Concept details

**Dataset 1** concepts were as follows:

- LEFT, RIGHT, STRAIGHT: Classification of driving direction concepts, trained with cross-entropy loss. For example, the concept LEFT represents training scenarios in which the car was turning left.
- STOP, SLOW: Classification of car speed concepts, trained with cross-entropy loss. The concept of, for example, STOP represents training scenarios in which the car was stopped.
- ASV (approaching stopped vehicle): Scenarios in which the car was approaching a stopped vehicle. Trained with binary cross-entropy.
- INTERSECTION: Scenarios in which the car was at an intersection. Trained with binary cross-entropy.
- CLOSE: Scenarios in which the car was within 3 m of another vehicle. Trained with binary cross-entropy.

**Dataset 2** had the following concepts:

- SLOW: Scenarios in which the car was driving at 1–2 m s^−1^.
- STOP: Scenarios in which the car was stationary.
- FAST: Scenarios in which the car was driving faster than 2 m s^−1^.
- STOP SIGN: Scenarios in which the car was close to a stop sign.
- TRAFFIC LIGHT: Scenarios in which the car was close to a traffic light.
- INTERSECTION: Scenarios in which the car was at an intersection.
- PEDESTRIAN: Scenarios in which the car was close to a pedestrian.
- FOLLOWING: Scenarios in which the car was following another vehicle.
- BIKE: Scenarios in which the car was close to a cyclist.
- PUDO (pedestrian pickup/drop-off): Scenarios in which the car was in a pedestrian pickup–drop-off zone.

All concepts in dataset 2 were trained with a binary cross-entropy loss.

Although the AV was trained on 8–10 concepts (depending on the dataset), we focus our study on a subset of concepts (CLOSE, ASV, BIKE and PEDESTRIAN) because they are the only ones that relate to other road users and are easy to test counterfactually. The concept FOLLOWING involves other road users too, but no notable occurrences happened during real-world deployment.

### Simulation results

We tested our CW-Net model across the entire nuPlan validation dataset to see how its driving performance compares with the original black-box ML planner it was trained from. The dataset is a large-scale planning benchmark for autonomous driving^39^ and measures how close a trained AV is to a human expert in *L*_2_ distance, progress along the route and safety (no collisions). In the black-box model, when following the lane or decelerating from high speed, the planner was able to make progress along the route (>93% of human driving distance), while avoiding collisions (>90% collision-free) and staying close to the ground-truth human expert trajectory (<1 m displacement at 5 s). Performance was worse when starting from a stop, with less progress (74% of human driving distance), more collisions (81% collision-free) and greater deviation from the human expert (1.2 m displacement at 5 s). Overall, the results showed our variation of the AV architecture had less than 0.01 *L*_2_ difference to the original black-box agent on average across all measurements, and not meaningfully different, showing that it is possible to train our more interpretable model in Fig. 1 without sacrificing performance. The full results are in Extended Data Table 1.

For concept accuracy verification, we used 5% holdout data from our training datasets; the results are given in Supplementary Tables 1 and 2. Across both datasets, the mean accuracy was 0.54, precision 0.23, recall 0.77 and F1 score 0.31. Overall, the results indicated that CW-Net did not separate all concepts equally well, which suits our purposes as the explanations will highlight when and how this happens, and how it relates to driving performance, thus helping with mental model refinement (see section ‘Concept separation’). Notable results include an F1 score of 0.82 for detecting the SLOW concept, and close to zero for detecting the BIKE concept, showing the latter is perhaps not well encoded or understood by the car.

### Mental model elicitation studies

These studies were designed to replicate the driver’s experiences in the private track tests (section ‘Mental model improvement in deployment’) with a larger cohort, to help demonstrate the robustness of our findings regarding the drivers’ mental models (Extended Data Fig. 2). The same study was run separately with experts (other drivers and test engineers from Motional) and non-experts (randomly sampled users from Prolific.com). Specifically, we were interested in testing the following hypotheses:

- Hypothesis 1: The participants’ responses before observing the explanation (in the video replay) would be more consistent with the belief of the safety driver before observing the explanation (in the car).
- Hypothesis 2: The participants’ responses after observing the explanation would be more consistent with the belief of the safety driver after observing the explanation.
- Hypothesis 3: The participants’ responses after observing the explanation would be more consistent with the ground-truth reason for AV behaviour.
- Hypothesis 4: Counterfactual prediction ability would correlate with user mental model goodness, the latter as measured by the nearest-neighbour tasks and the free-form text response in ref. ^43^.
- Hypothesis 5: The two measures of mental model goodness would correlate with each other and have similar distributions across groups.

#### Design and materials

We focused on the same three surprising events observed during the private track tests. The experiment was designed to measure mental models through a combination of nearest-neighbour and prediction tasks, accompanied by confidence scores and free-form text responses, in which we could probe the participants’ mental models^43^ (Extended Data Fig. 2). First, users saw the respective video and were asked to rate two possible reasons for AV behaviour, along with confidence scores (that is, the nearest-neighbour mental model elicitation task). Then, users were asked to make the same counterfactual prediction as the driver (that is, the prediction task), along with a confidence score, and a free-form text rationale explaining their reasoning. Then, users saw the same video with the explanation and repeated the questions. This within-participant design mirrors the experience of the safety drivers during the on-road tests.

#### Participants

For the expert group, we recruited nine safety drivers, test engineers and test specialists from Motional (aged between 18 years and 80 years, 8 male and 1 female). All participants volunteered to participate and were not paid. For non-experts, we sampled users sourced from Prolific.com (www.prolific.com), which is known for its high-quality user base. Thirty users were randomly sampled US citizens aged between 18 years and 80 years, native English speakers and an even number of male and female. To ensure high-quality text responses, we paid users above the average rate with USD15 per hour and stressed that they should take the study only if they were certain they understood the instructions. The study received MIT IRB approval from the Committee on the Use of Humans as Experimental Subjects, exempt ID: E-5903, start date 1 July 2024, end date 31 August 2026.

#### Metrics

We used the nearest-neighbour task and the free-form text rationale as direct measures of mental models. For the nearest-neighbour task, we measured mental model goodness as a combination of choice accuracy and confidence. Specifically, we formalized mental model improvement as shifting from an incorrect to a correct belief (with respect to the ground-truth reasons for AV behaviour), or increasing confidence in the correct belief, or decreasing confidence in the incorrect belief (Extended Data Fig. 3). For the free-form text rationale, we used an LLM-as-a-judge (GPT-5) to determine which text response (before or after observing the CW-Net explanation) is closer to the ground-truth reason for AV behaviour (see Supplementary Methods for details). The LLM prompts were tuned over three iterations on the expert responses (Extended Data Fig. 4a) and then evaluated once on the non-expert responses (Extended Data Fig. 4b). Mental model improvement was formalized as instances where the free-form response after observing the CW-Net explanation is closer to the ground truth, compared with the free-form response before observing the explanation.

We used the prediction task as a measure of downstream performance that relies on mental models, thus measuring them indirectly^44,58^. We measured prediction improvement as a combination of choice accuracy and confidence in the same way as for the nearest-neighbour task (Extended Data Fig. 5).

To analyse the relationship between direct (nearest neighbour, free-form text rationale) and indirect (prediction) measures of mental models, we used a linear mixed-effects model (LME) to evaluate the effect of the mental model change (improve or worsen) on the prediction change variable (that is, the delta in confidence change in the prediction), while controlling for individual participant variation as a random effect (Extended Data Fig. 5). To collapse accuracy and confidence on a single scale for computing the confidence deltas, we simply flipped the sign of confidence values for inaccurate beliefs to negative. All code for this analysis will be available on publication (see section ‘Code availability’ below).

#### Study conclusions

We found evidence favouring all of our hypotheses.

- Hypothesis 1: The participants’ initial belief (before observing the explanation) was often more similar to the safety driver’s* initial* belief (*P* < 10^−9^, exact binomial test).
- Hypothesis 2: The participants’ final belief (after observing the explanation) was often times more similar to the safety driver’s final belief (*P* < 0.0002, exact binomial test).
- Hypothesis 3: The participants’ final belief (after observing the explanation) was often times more similar to the ground truth (*P* < 10^−5^, exact binomial test).
- Hypothesis 4: There was a clear relationship between mental model category and predictive ability of users, with only free-form text response categories failing to reach significance with experts (Extended Data Fig. 5).
- Hypothesis 5: Interaction analysis showed no significant difference in the relationship between nearest-neighbour score improvement and text rationale improvement across the two groups (*P* = 0.842; Extended Data Fig. 4c), indicating a consistent underlying mechanism for both experts and non-experts. Non-experts demonstrated a significant positive correlation (*β* = 0.27, *P* < 0.001), suggesting that improved scores were strong predictors of improved mental model rationale in this group. Although experts exhibited a nearly identical positive coefficient (*β* = 0.23), the relationship did not reach statistical significance, probably because of a smaller sample size (*P* = 0.243).

### Public roads evaluation using SAGAT

We deployed CW-Net in manual mode on Las Vegas public roads to collect complex, naturalistic scenarios analogous to those already discovered during the private track tests (Extended Data Fig. 6). We used these as materials for an online SAGAT study (*n* = 100). This setup allows us to validate the robustness of the CW-Net explanations using a well-established rigorous framework (Extended Data Fig. 2). We used a between-participant design in which we compared CW-Net explanations (experimental group) against baseline explanations describing speed and steering (control group)^49^ (Extended Data Fig. 7).

#### Gathering materials

We collected data for ASV (01:02:55), CLOSE (00:50:42), and BIKE ( > 3 hours). We labelled sequences where human driving mimicked the ML planner in surprising scenarios that resembled those already discovered in the private track tests (for example, CLOSE: stuck beside vehicles; ASV: braking for hallucinations; Extended Data Fig. 6). PEDESTRIAN replaced BIKE because there were no naturally occurring cyclists in the new video data. We sampled two surprising events per concept (6 total) and six corresponding ‘unsurprising’ events to ensure explanations did not degrade situational awareness in regular driving (Supplementary Table 5). Activation thresholds were 0.5 for ASV/PEDESTRIAN and 0.94 for CLOSE, derived from prior private track data (Fig. 3).

Note the distribution of concept activations did not change significantly between the private-track tests and the public-road tests (Supplementary Table 7), despite the tests being performed more than a year apart, on different AVs, with different software stacks and under completely different conditions. This demonstrates the robustness and reliability of the algorithm.

#### Study design

We used a between-participants design (*n* = 100) assessing SAGAT perception, comprehension, and projection. Participants were split into experimental (CW-Net explanations) and control (speed/steering placeholder) groups. Attention checks based on material content and viewing times reduced the pool to 99 participants, 51 in the experimental and 48 in the control.

#### Materials

Stimuli included 13 videos (6 surprising, 6 unsurprising and 1 attention check). The experimental group saw concept activations, whereas the control saw speed and steering data (Extended Data Fig. 8). Following each video, a ‘blackout’ screen presented six binary questions: four related to perception, one to comprehension and one to projection.

#### Participants

We recruited gender-balanced US residents (18+ years, native English speakers) by Prolific.com. Participants were paid $12 per hour. The study received MIT IRB approval from Committee on the Use of Humans as Experimental Subjects, exempt ID: E-5903, start date 1 July 2024, end date 31 August 2026.

#### Metrics

We analysed the average of each participant on each question type using two-tailed *t*-tests, splitting data by surprising and unsurprising events for each situational awareness dimension.

#### Study conclusions

After collection and attention check filtering, we collected 99 responses out of the target 100. The results are shown in Extended Data Fig. 7. In surprising events, explanations significantly improved situational awareness after Bonferroni correction, showing large effect sizes for perception (Cohen’s *d* = 1.290) and comprehension (*d* = 0.996), alongside a medium effect for projection (*d* = 0.606). In unsurprising events, no significant differences occurred (perception *d* = 0.085, projection *d* = −0.142, comprehension *d* = −0.514), confirming that explanations provided benefit in anomalous situations without adversely affecting situational awareness during routine operations. Furthermore, we confirmed feature robustness by comparing concept distributions to previous private track tests using Wasserstein distance (Supplementary Table 7), finding no meaningful changes.

### Reporting summary

Further information on research design is available in the Nature Portfolio Reporting Summary linked to this article.

## Online content

Any methods, additional references, Nature Portfolio reporting summaries, source data, extended data, supplementary information, acknowledgements, peer review information; details of author contributions and competing interests; and statements of data and code availability are available at https://doi.org/10.1038/s41586-026-10950-5.

## Supplementary information

Supplementary Information This file contains two Supplementary Methods sections detailing free-form response evaluation and perception item reliability. It includes Supplementary Tables 1–9, collectively demonstrating CW-Net classification performance, mental model prediction accuracy, SAGAT study results, distribution shifts and statistical consistency.

Reporting Summary

Peer Review File

Supplementary Data (Extended Data Table 1) This file contains source data for Extended Data Table 1.

## Source data

Source Data Fig. 3

Source Data Extended Data Fig. 3

Source Data Extended Data Fig. 4

Source Data Extended Data Fig. 5

Source Data Extended Data Fig. 7

## Figures and Tables

**Extended Data Table 1:**
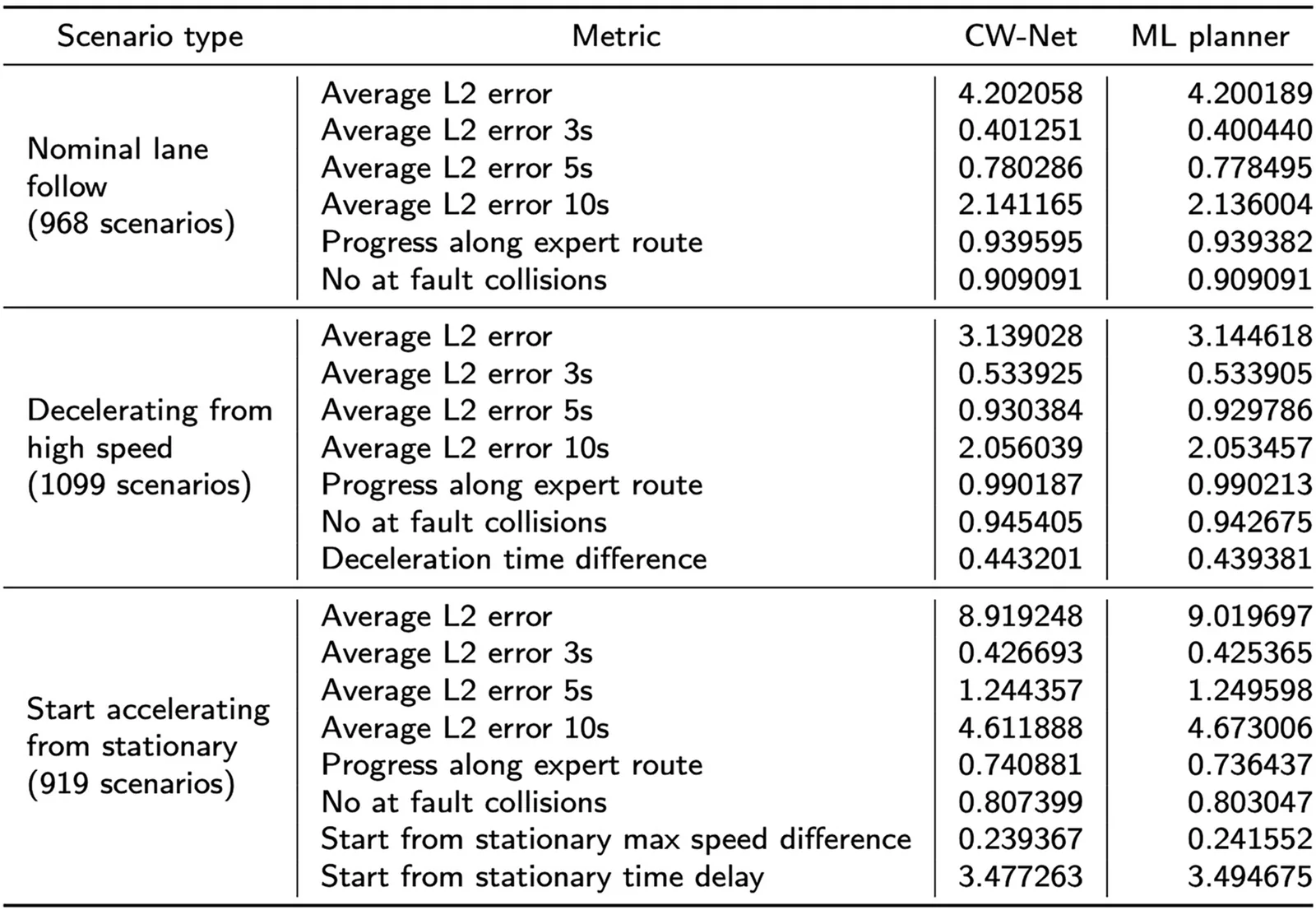
Full nuPlan results

Comparing driving performance of our CW-Net planner to the original black-box ML planner, showing minimal difference.

Source Data

## Data Availability

The data used for plotting the figures in this study are available at GitHub (https://github.com/EoinKenny/CW-Net-Autonomous-Driving), with the exception of Extended Data Table 1, Supplementary Tables 1 and 2. The AV model weights are not available because of intellectual property restrictions. The videos of CW-Net and corresponding explanations shown to participants in our studies are available on the project website at GitHub (https://tomov.github.io/CW-Net/). Source data are provided with this paper.

## Code availability

The code for reproducing the plots in this study is available at GitHub (https://github.com/EoinKenny/CW-Net-Autonomous-Driving). Owing to Motional intellectual property issues, the code for training the real-world AV used in the paper cannot be made available. However, we have provided a Code Ocean capsule that reproduces the algorithm in another toy self-driving domain.
