# Supplementary material for: Explainable deep learning improves human mental models of self-driving cars
Source: Nature. 2026 Sep 2;657(8130):114–20. doi: 10.1038/s41586-026-10950-5 (PMC13538056; doi:10.1038/s41586-026-10950-5)
Supplement: Supplementary file 1 [file 41586_2026_10950_MOESM1_ESM.pdf]

nature portfolio

https://doi.org/10.1038/s41586-026-10950-5

Supplementary information

Explainable deep learning improves human mental models of self-driving cars

In the format provided by the authors and unedited

Supplementary Information

Supplemental Methods

Evaluating free-form human responses

We used a large language model (LLM) as a cost-effective, reproducible, and unbiased way to semantically parse free-form text responses. We used the following prompts:

- Instruction prompt: *"Imagine you're a human participant in a psychological study. You are given a anchor mental model and prediction (denoted by ANCHOR) for the behavior of an autonomous vehicle (AV) and two candidate explanations and predictions for AV behavior (denoted by CANDIDATE 1 and CANDIDATE 2) given by passengers in the AV. Please indicate which of the two candidate explanations and predictions are most consistent with the anchor mental model and prediction. Respond simply with the number of the candidate (1 or 2)."*
- Input prompt:
  "ANCHOR: ⟨anchor⟩
  CANDIDATE 1: ⟨before⟩
  CANDIDATE 2: ⟨after⟩"

The input prompt was unique for every (participant, scenario, anchor) tuple. ⟨*before*⟩ and ⟨*after*⟩ were the participants' free-form responses before and after observing the explanation, respectively. There were three ⟨anchor⟩'s for each scenario, based on the private track tests and our post-hoc analyses (see main paper results figure) – the safety driver's initial belief, the safety driver's final belief, and the ground truth reason for AV behavior:

- CLOSE scenario:
  - Anchor 1 – safety driver's initial belief: *"The AV stopped because of the pickup/drop-off zone ahead. Therefore, if the AV was slightly to the left, it would not change its behavior; it would still stop."*,
  - Anchor 2 – safety driver's final belief: *"The AV stopped because of the parked cars nearby to the right. If it was further to the left, it would be further from the parked cars, and therefore it would keep moving."*,
  - Anchor 3 – ground truth: *"The AV stopped because of the parked cars nearby to the right. If it was further to the left, it would be further from the parked cars, and therefore it would keep moving."*,
- ASV scenario:
  - Anchor 1 – safety driver's initial belief: *"The AV stopped because of the traffic cone. Therefore, if the traffic cone was not there, it would have kept moving."*,
  - Anchor 2 – safety driver's final belief: *"The AV stopped because it hallucinated a stopped vehicle, not because of the traffic cone. Therefore, if the traffic cone was not there, it may have still stopped."*,
  - Anchor 3 – ground truth: *"The AV stopped because it hallucinated a stopped vehicle, not because of the traffic cone. Therefore, if the traffic cone was not there, it would have still stopped."*,

- BIKE scenario:
  - ANCHOR 1 – safety driver's initial belief: *"The AV can detect and stop for cyclists reliably, which is why it stopped for the cyclist. Therefore, we can trust that the AV will keep stopping reliably for cyclists in the future."*,
  - ANCHOR 2 – safety driver's final belief: *"The AV cannot detect and stop for cyclists reliably. Therefore, we cannot trust that the AV will keep stopping reliably for cyclists in the future."*,
  - ANCHOR 3 – ground truth: *"The AV cannot detect and respond to cyclists at all; instead, it stopped because of the automatic emergency braking system. Therefore, we cannot trust that the AV will keep stopping reliably for cyclists in the future."*,

We used GPT-5, a state-of-the-art reasoning LLM. The prompts were tuned over 3 iterations on the expert responses and then evaluated once on the non-expert responses (see Extended Data). All code and data for this analysis is available at https://github.com/EoinKenny/CW-Net-Autonomous-Driving.

To validate the judgments of the LLM, we performed an interrater reliability check by having two human raters act as judges for 6 of the non-expert participants' responses. The human judges were presented with the same prompts as the LLM with the goal of checking if the their judgements match those of the LLM. This resulted in a total of 54 human judgments (6 participants $\times$ 3 scenarios $\times$ 3 anchors) which were compared with each other and with the corresponding LLM judgments.

Interrater reliability between the human judges was substantial (Cohen's κ = 0.66), with agreement on 45 of the 54 (83%) of judgments. The LLM had similar agreement with the two human judges: 85% (46/54 judgments; Cohen's κ = 0.70) with the first judge and 80% (43/54 judgments; Cohen's κ = 0.59) with the second judge. For the 45 queries on which the humans agreed with each other, the LLM achieved an even higher overall accuracy of 89% (40/45 judgments; Cohen's κ = 0.77). Together, these results indicate that the LLM judgments can serve as reliable substitutes for human judgments of the participants' free-form text responses.

Reliability of perception items

We report the internal consistency of the SAGAT perception items and provide additional diagnostics to clarify how the composite perception score should be interpreted.

Measurement structure.

We treat perception as a single latent construct measured by 48 binary indicators: 12 materials $\times$ 4 items per material. Each item probes a distinct concept. Across the full dataset, the resulting participant-by-item matrix has size 99×48. Because our main analyses use the aggregated composite perception score, reliability at this aggregated level is the relevant quantity for our manuscript.

Classical Cronbach's alpha.

Computing Cronbach's alpha on the full 99×48 binary response matrix gives $\alpha = 0.754$, which is above the conventional 0.70 threshold for acceptable internal consistency (Table S8). For binary items, this calculation is equivalent to the KR-20 reliability coefficient. We therefore consider the full perception composite sufficiently reliable for the purposes of our analysis.

Interpretation for binary items.

Alpha for binary correct/incorrect responses can be conservative when many items are near ceiling or floor. In such cases, limited item variance reduces the observed inter-item correlations, which can in turn depress alpha even when the items reflect a common underlying construct. This issue is relevant here because several of the world-state items were answered correctly by nearly all participants.

Item structure and ceiling effects.

Our perception items divide naturally into two groups. World-state items (Q1 and Q3) ask what was present in the video, whereas AV-state items (Q2 and Q4) ask what the autonomous vehicle detected. The world-state items were closer to ceiling: pooled mean accuracy was 0.858 for Q1 and 0.846 for Q3 across $n = 99$ participants, with 9 of 12 material-level Q1 items near ceiling, defined as accuracy ≥ 0.85 (Table S9). By comparison, pooled mean accuracy was 0.786 for Q2 and 0.785 for Q4. The AV-state items also exhibited greater item-level response variance than the world-state items, making them less affected by ceiling restriction (Table S9).

Subscale reliability.

For completeness, we also computed classical Cronbach's alpha separately for the AV-state and world-state subscales. The AV-state subscale achieves $\alpha = 0.792$, indicating good internal consistency. This is the component most directly targeted by the CW-Net explanations used in the main paper. The world-state subscale has a lower classical estimate, $\alpha = 0.408$, consistent with its substantially stronger ceiling restriction and lower item-level variance (Table S8).

Ordinal alpha.

As a complementary reliability analysis for these binary items, we additionally report ordinal alpha. Ordinal alpha estimates reliability on an underlying latent response scale by using tetrachoric correlations rather than raw correlations between observed $0/1$ responses. This is appropriate when binary responses are viewed as discretized observations of an underlying continuous trait. Using this approach, ordinal alpha is 0.929 for the full perception scale, 0.907 for the AV-state subscale, and 0.862 for the world-state subscale (Table S8). These values indicate that the binary-response format and associated ceiling effects likely make the classical alpha estimates conservative, particularly for the world-state items.

Excluded item for ordinal alpha.

One item, an1–Q1, was excluded from the ordinal-alpha calculation because all 99 participants answered it correctly, resulting in zero variance. Since tetrachoric correlations are not estimable for an invariant item, the full-scale ordinal-alpha estimate is based on 47 of the 48 items, and the world-state ordinal-alpha estimate is based on 23 of the 24 items. The AV-state ordinal-alpha estimate uses all 24 AV-state items (Table S8).

Summary.

Taken together, these analyses support the use of the composite perception score in the main text. Classical alpha on the full set of perception items is acceptable, the AV-state subscale is reliable on its own, and ordinal alpha shows that the binary-response format and ceiling effects likely attenuate the classical estimates.

Supplemental Tables

	Accuracy	Precision	Recall	F1 Score
Steering	0.62	(cross entropy)		
Speed	0.83	(cross entropy)		
Approaching stopped vechicle (ASV)	0.55	0.20	0.89	0.33
Intersection	0.51	0.42	0.61	0.49
CLOSE to another vechicle	0.45	0.10	0.81	0.17
Ranker accuracy	0.94			

Table S1 CW-Net concept classification performance on Dataset 1 on holdout validation data.

	Accuracy	Precision	Recall	F1 Score
Slow	0.83	0.73	0.93	0.82
Stopped	0.48	0.16	0.81	0.27
Fast	0.68	0.49	0.86	0.63
Stop sign	0.43	0.03	0.84	0.05
Traffic light	0.39	0.16	0.62	0.25
Intersection	0.42	0.20	0.65	0.31
Pedestrian	0.44	0.03	0.84	0.07
Following	0.43	0.02	0.84	0.04
BIKE	0.21	0.00	0.42	0.00
PUDO	0.58	0.30	0.86	0.44
Ranker accuracy	0.95			

Table S2 CW-Net concept classification performance on Dataset 2 on holdout validation data.

Scenario/concept	Before explanation	After explanation
CLOSE	0.00%	11.11%
ASV	33.33%	44.44%
BIKE	77.78%	77.78%
**Overall average**	37.04%	44.44%

Table S3 Performance improvement on prediction task ($N = 9$ experts).

Scenario/concept	Before explanation	After explanation
CLOSE	3.33%	33.33%
ASV	36.67%	60.00%
BIKE	40.00%	66.67%
**Overall average**	26.67%	53.33%

Table S4 Performance improvement on prediction task ($N = 30$ non-experts).

Surprising events			
	Concept activation	AV speed	Concept accuracy
	Original		
ASV	High	Brakes	Bad
CLOSE	High	Frozen	Good
BIKE	Low	Moving	Low
	Modified for unsurprising events		
ASV	High	Brakes	**Good**
CLOSE	**Low**	Frozen	Good
BIKE	Low	**Stopped**	Low

Table S5 Selecting unsurprising events for SAGAT study. To select unsurprising events we modifed what was unusual about each surprising event which fell into three categories. **ASV**: This concept was surprising because of the misclassification of the concept, so we selected a scenario in which it accurately classified the concept. **CLOSE**: This was surprising because the concept activation was higher than usual while the AV stopped, so we chose scenarios in which the AV stopped, but the concept activation was closer to its mean value. **BIKE**: This was unusual because the AV stopped seemingly due to the cyclist, so we chose scenarios in which the AV again stopped for other reasons. In all three setups, we only altered the one respective dimension, the other two remained static.

**Valence**	**Dimension**	**Effect direction**	**Cohen's d**	**95% CI**	p**-value**	**Bonferroni significant**
Surprising events	Perception	Exp > Control	1.290 (large)	$[0.857, 1.723]$	< 0.001	Yes
	Comprehension	Exp > Control	0.996 (large)	$[0.578, 1.413]$	< 0.001	Yes
	Projection	Exp > Control	0.606 (medium)	$[0.203, 1.009]$	0.003	Yes
Unsurprising events	Perception	No effect	0.085 (negligible)	$[-0.310, 0.479]$	0.667	No
	Comprehension	Control > Exp	-0.514 (medium)	$[-0.915, -0.114]$	0.011	No
	Projection	No effect	-0.142 (negligible)	$[-0.536, 0.253]$	0.481	No

Table S6 SAGAT study results. During the 'surprising' events, explanations had an all-around positive impact on people's situational awareness and by extension their mental models. Conversely, during 'unsurprising' events, there were no significant differences between groups, after Bonferroni correction.

FEATURE	Model 1	Model 2	Model 3
STOPPED	0.0862	0.0014	0.0324
SLOW	0.1575	0.0014	0.0324
FAST	0.1903	—	—
STOP SIGN	0.2368	—	—
TRAFFIC LIGHT	0.0938	—	—
INTERSECTION	0.0856	0.0247	0.0535
PEDESTRIAN	0.0998	—	—
FOLLOWING	0.1056	—	—
BIKE	0.1222	—	—
PUDO	0.2347	—	—
LEFT	—	0.0472	0.0332
RIGHT	—	0.0408	0.0410
STRAIGHT	—	0.0428	0.0592
ASV	—	0.0269	0.1050
CLOSE	—	0.0659	0.0702

Table S7 Concept distribution shift: Wasserstein distances between the distribution of concept probabilities during the semi-naturalistic tests on the private track and the subsequent public-road test. Overall, the differences varied from mostly insignificant to low/medium in `STOP SIGN` and `PUDO`.

Table S8 Internal-consistency estimates for the SAGAT perception items. Classical Cronbach's alpha was computed on the raw binary item matrix. Ordinal alpha was computed from the tetrachoric item-correlation matrix after excluding zero-variance items. The full-scale and world-state ordinal-alpha estimates exclude an1–Q1, which was answered correctly by all 99 participants.

Scale	n	Items for classical α	Items for ordinal α	Classical α	Ordinal α
Full perception scale (Q1–Q4)	99	48	47	0.754	0.929
AV-state subscale (Q2, Q4)	99	24	24	0.792	0.907
World-state subscale (Q1, Q3)	99	24	23	0.408	0.862

Table S9 Response-distribution diagnostics for the four SAGAT perception item types. Pooled mean accuracy is computed across all 99 participants and 12 materials for each question type. Mean item variance is the average variance across the 12 material-level binary indicators for that question. "Near ceiling" denotes material-level items with accuracy ≥ 0.85.

Question	Item type	Pooled mean accuracy	Mean item variance	Near-ceiling items
Q1	World-state	0.858	0.094	9/12
Q2	AV-state	0.786	0.130	7/12
Q3	World-state	0.846	0.105	7/12
Q4	AV-state	0.785	0.124	7/12
