# Supplementary material for: Explainable deep learning improves human mental models of self-driving cars
Source: Nature. 2026 Sep 2;657(8130):114–20. doi: 10.1038/s41586-026-10950-5 (PMC13538056; doi:10.1038/s41586-026-10950-5)
Supplement: Supplementary file 2 [file 41586_2026_10950_MOESM2_ESM.pdf]

Corresponding author(s): Eoin M. Kenny
Last updated by author(s): Aug 19, 2026

Reporting Summary

Nature Portfolio wishes to improve the reproducibility of the work that we publish. This form provides structure for consistency and transparency in reporting. For further information on Nature Portfolio policies, see our Editorial Policies and the Editorial Policy Checklist.

Statistics

For all statistical analyses, confirm that the following items are present in the figure legend, table legend, main text, or Methods section.

n/a	Confirmed	
☐	☒	The exact sample size (*n*) for each experimental group/condition, given as a discrete number and unit of measurement
☐	☒	A statement on whether measurements were taken from distinct samples or whether the same sample was measured repeatedly
☐	☒	The statistical test(s) used AND whether they are one- or two-sided<br>*Only common tests should be described solely by name; describe more complex techniques in the Methods section.*
☒	☐	A description of all covariates tested
☐	☒	A description of any assumptions or corrections, such as tests of normality and adjustment for multiple comparisons
☐	☒	A full description of the statistical parameters including central tendency (e.g. means) or other basic estimates (e.g. regression coefficient) AND variation (e.g. standard deviation) or associated estimates of uncertainty (e.g. confidence intervals)
☐	☒	For null hypothesis testing, the test statistic (e.g. *F*, *t*, *r*) with confidence intervals, effect sizes, degrees of freedom and *P* value noted<br>*Give P values as exact values whenever suitable.*
☒	☐	For Bayesian analysis, information on the choice of priors and Markov chain Monte Carlo settings
☐	☒	For hierarchical and complex designs, identification of the appropriate level for tests and full reporting of outcomes
☐	☒	Estimates of effect sizes (e.g. Cohen's *d*, Pearson's *r*), indicating how they were calculated

Our web collection on statistics for biologists contains articles on many of the points above.

Software and code

Policy information about availability of computer code

Data collection	Most of the code used to collect data is IP of Motional and cannot be shared. However, code related to CW-Net itself is freely available at the paper's GitHub repo: https://github.com/EoinKenny/CW-Net-Autonomous-Driving/tree/main
Data analysis	All code/data for analysing the data is freely available, and fully reproducible at: https://github.com/EoinKenny/CW-Net-Autonomous-Driving/tree/main

For manuscripts utilizing custom algorithms or software that are central to the research but not yet described in published literature, software must be made available to editors and reviewers. We strongly encourage code deposition in a community repository (e.g. GitHub). See the Nature Portfolio guidelines for submitting code & software for further information.

Data

Policy information about availability of data

All manuscripts must include a data availability statement. This statement should provide the following information, where applicable:

- Accession codes, unique identifiers, or web links for publicly available datasets
- A description of any restrictions on data availability
- For clinical datasets or third party data, please ensure that the statement adheres to our policy

The data used for plotting the figures in this study are available at GitHub (https://github.com/EoinKenny/CW-Net-Autonomous-Driving), with the exception of Extended Data Table 1, Supplementary Tables 1 and 2. The AV model weights are not available because of intellectual property restrictions. The videos of CW-Net

and corresponding explanations shown to participants in our studies are available on the project web- site at GitHub (https://tomov.github.io/CW-Net/). Source data are provided with this paper.

Research involving human participants, their data, or biological material

Policy information about studies with human participants or human data. See also policy information about sex, gender (identity/presentation), and sexual orientation and race, ethnicity and racism.

Reporting on sex and gender	Sex (biological attribute) was recorded at group level only and was not an experimental variable. Expert group (n = 9 Motional safety drivers, test engineers and test specialists): 8 male, 1 female — this reflects the available employee pool rather than a design choice. Non-expert elicitation group (n = 30): recruited to be balanced, 15 male and 15 female. SAGAT study (n = 99 analysed): recruited to be sex-balanced via Prolific prescreening. In all cases this was self-reported by participants at recruitment, not assigned by the researchers, prolific ensures that all groups are balanced in this regard. Biological sex was not analysed or recorded by the researchers, but it was controlled during recruitment when possible.
Reporting on race, ethnicity, or other socially relevant groupings	Race and ethnicity data were not collected and no such variables are used in this manuscript. The only participant categorisation used is expertise: experts are Motional safety drivers, test engineers and test specialists with professional experience operating and testing the AV platform; non-experts are general-population participants with no such experience. Classification was by recruitment channel (direct recruitment at Motional versus Prolific.com), not self-report. Nationality and language (US citizen/resident, native English speaker) were used solely as eligibility criteria and were self-reported through Prolific prescreening; they are not used as analysis variables or as proxies for any other characteristic. No adjustment for socially constructed confounders was performed.
Population characteristics	Experts (n = 9): Motional safety drivers, test engineers and test specialists, age between 18-80, with professional experience of the AV platform used; unpaid volunteers. Non-experts, elicitation study (n = 30): US citizens, aged 18–80, native English speakers, no professional AV experience. SAGAT study (n = 99 of 103 submitted) US residents aged 18 or over, native English speakers, sex-balanced, recruited independently of the elicitation study. Semi-naturalistic private-track study: n = 3 trained Motional safety drivers. Expertise is the only covariate-relevant characteristic entering any analysis. No clinical, health, genotypic, diagnosis or treatment information was collected. Mean age not collected.
Recruitment	Experts were recruited directly from Motional's AV testing operations; they volunteered and were not paid. Non-experts were recruited through Prolific.com with prescreening for US citizenship/residency, native English, age, and gender balance, and were paid above the platform average (USD 15 per hour, elicitation study; USD 12 per hour, SAGAT study). Participants were instructed to take part only if they were certain they understood the instructions.<br>Potential biases: (i) experts were colleagues of the study team with prior familiarity with the vehicle — the parallel non-expert sample was run to address this, and showed the same effects at equal or larger magnitude; (ii) online panel participants self-select and may be more attentive and more technology-familiar than the general population; (iii) the private-track safety drivers were not randomly sampled and that study is descriptive, which is why the online studies were conducted to test generalisation; (iv) four SAGAT participants was excluded on attention checks, viewing time, and incomplete studies.
Ethics oversight	All human-participant studies were approved by the Massachusetts Institute of Technology Committee on the Use of Humans as Experimental Subjects (COUHES), exempt protocol ID E-5903. The original field study (n=3) did not qualify as human subject research.

Note that full information on the approval of the study protocol must also be provided in the manuscript.

Field-specific reporting

Please select the one below that is the best fit for your research. If you are not sure, read the appropriate sections before making your selection.

☐ Life sciences ☒ Behavioural & social sciences ☐ Ecological, evolutionary & environmental sciences

For a reference copy of the document with all sections, see nature.com/documents/nr-reporting-summary-flat.pdf

Behavioural & social sciences study design

All studies must disclose on these points even when the disclosure is negative.

Study description	Mixed-methods, three studies. (1) A semi-naturalistic observational study on a private test track in which safety drivers operated a real AV running CW-Net; quantitative vehicle telemetry and concept activations were recorded alongside qualitative think-aloud reports and counterfactual predictions. (2) A quantitative within-participant online experiment (mental model elicitation) in which participants answered nearest-neighbour and prediction items, with confidence scores and free-form text rationales, before and after seeing CW-Net explanations. (3) A quantitative between-participants online experiment using the Situation Awareness Global Assessment Technique (SAGAT), comparing CW-Net explanations against speed/steering placeholder explanations on replays from public-road AV operation.
Research sample	Study 1: n = 3 Motional safety drivers operating the AV during routine testing; chosen because they are the target end users of in-vehicle explanations and the only population able to interact with a live AV.<br>Study 2: 9 experts (Motional safety drivers, test engineers and test specialists; age screened to be 18-80; 8 male, 1 female; unpaid volunteers) and 30 non-experts (US citizens, aged 18–80, native English speakers, even split male/female) recruited via Prolific.com.

Both groups were sampled to test whether the effects observed in Study 1 hold for domain experts and for the general public alike.
Study 3: 103 participants submitted responses (51 control, 52 experimental); 99 were analysed (48 control, 51 experimental).; US residents aged 18+, native English speakers, sex-balanced, recruited via Prolific.com.
Samples are not intended to be nationally representative. The expert samples are small and drawn from a single AV company, which is inherent to the population of interest; the non-expert samples are general-population online panels and are broadly representative of English-speaking US adults on that platform.

Sampling strategy	Study 1: convenience sampling of the safety drivers assigned to the test programme; the surprising events analysed occurred naturally and were not pre-planned.<br>Study 2: convenience/census sampling of available Motional drivers and engineers (experts); pseudo-random sampling from the Prolific.com participant pool with prescreening on citizenship, age, native language and sex balance (non-experts).<br>Study 3: pseudo-random sampling from Prolific.com with the same prescreening criteria; participants who had taken Study 2 were not eligible.<br>No a priori power analysis was performed. Sample sizes were chosen pragmatically: the expert n was limited by the number of qualified personnel available, and the non-expert and SAGAT sample sizes follow conventions in the XAI and situation-awareness user-study literature. Sufficiency is supported post hoc by the observed effect sizes (Cohen's d = 1.290, 0.996 and 0.606 for SAGAT perception, comprehension and projection in surprising events) and by consistent effects across two independently recruited non-expert samples. Data saturation was not applicable, as qualitative think-aloud data were used descriptively rather than for thematic saturation.
Data collection	Study 1: conducted in a real AV on a private track using the Lab2Car wrapper. Present in the vehicle were the safety driver, a support engineer who deployed CW-Net and set destinations, and a researcher who directed testing. Data recorded were vehicle telemetry and CW-Net concept probabilities logged by the onboard system, an in-cabin dashboard display showing perception detections, the planned trajectory and concept activations as percentages, and researcher-recorded think-aloud reports. Public-road data in Las Vegas were collected with the AV in manual mode and CW-Net running in the background. Researchers were not blind to condition or hypothesis; the study was observational and exploratory, and the counterfactual manipulations (moving the vehicle away from parked cars, removing the traffic cone) were physically verifiable rather than researcher-scored.<br>Studies 2 and 3: computer-administered online surveys hosted by Qualtrics/Prolific and completed by participants alone and unsupervised. Stimuli were front-camera video replays with overlaid explanations. Study 3 used a blackout screen after each video followed by six binary questions. Data collection was automated, so no researcher was present and no researcher judgement entered response recording; free-form text responses in Study 2 were scored by an LLM-as-a-judge (GPT-5) blind to whether a response was collected before or after the explanation. Participants were aware of the general purpose of the study but not of the specific hypotheses.
Timing	Original semi-naturalistic field study: March 25-28 2024<br>Mental model study experts: 7 Nov 2025<br>Mental model study non-experts: 13 Nov 2025<br>Second data collection in car for SAGAT: April 7-23 2025.<br>SAGAT: 9 Sep 2025
Data exclusions	Studies 1 and 2 (private-track deployment; mental model elicitation): no data were excluded.<br>Study 3 (SAGAT): of 103 submissions (51 control, 52 experimental), 4 were excluded, leaving 99 (48 control, 51 experimental). Two participants (one per group) abandoned the survey without providing any scoreable responses and were removed. A further two participants, both in the control group, failed the embedded attention check — one of these had also abandoned the survey partway through, completing 36 of 72 scored items. Both criteria were pre-established, implemented in the analysis code, and applied identically to both groups. Minimum viewing-time thresholds were also specified per stimulus; applying them excluded no additional participants. Results are unchanged in a complete-cases-only sensitivity analysis.
Non-participation	Study 2: all 9 experts approached agreed to take part; no expert or non-expert participant withdrew after beginning the survey.<br>Study 3: 3 of 103 participants (2.9%) abandoned the survey after starting — two with no scoreable responses (progress 20% and 0% respectively) and one after completing half of the scored items.
Randomization	Study 1: no allocation to groups; all safety drivers experienced the same conditions and served as their own controls through counterfactual manipulations.<br>Study 2: participants were not allocated to groups. The design was within-participant — every participant answered the same items before and after seeing the explanation, in fixed order, since the manipulation is the provision of the explanation and cannot be counterbalanced. Expert versus non-expert is a pre-existing grouping, not an allocation.<br>Study 3: participants were allocated to the experimental (CW-Net explanations) or control (speed/steering placeholder) group at random by Prolific. Both groups saw identical videos and identical questions, and the control explanations were designed to match cognitive load, controlling for display-related covariates.

Reporting for specific materials, systems and methods

We require information from authors about some types of materials, experimental systems and methods used in many studies. Here, indicate whether each material, system or method listed is relevant to your study. If you are not sure if a list item applies to your research, read the appropriate section before selecting a response.

Materials & experimental systems

n/a	Involved in the study
☒	☐ Antibodies
☒	☐ Eukaryotic cell lines
☒	☐ Palaeontology and archaeology
☒	☐ Animals and other organisms
☒	☐ Clinical data
☒	☐ Dual use research of concern
☒	☐ Plants

Methods

n/a	Involved in the study
☒	☐ ChIP-seq
☒	☐ Flow cytometry
☒	☐ MRI-based neuroimaging

Plants

Seed stocks	N/A
Novel plant genotypes	N/A
Authentication	N/A
