# Supplementary material for: Explainable deep learning improves human mental models of self-driving cars
Source: Nature. 2026 Sep 2;657(8130):114–20. doi: 10.1038/s41586-026-10950-5 (PMC13538056; doi:10.1038/s41586-026-10950-5)
Supplement: Supplementary file 3 [file 41586_2026_10950_MOESM3_ESM.pdf]

nature portfolio

Peer Review File

Explainable deep learning improves human mental models of self-driving cars

Corresponding Author: Dr Eoin Kenny

Any redactions in this file are there to maintain patient confidentiality, the confidentiality of unpublished data, or to remove third-party material.

This file contains all reviewer reports in order by version, followed by all author rebuttals in order by version.

Version 0:

Reviewer comments:

Referee #1

(Remarks to the Author)
Topic
The paper introduces the Concept-Wrapper Network (CW-Net), a framework designed to enhance the interpretability of deep learning-based motion planners in autonomous vehicles (AVs). By grounding the decision-making process in human-interpretable concepts, CW-Net is claimed to align the mental models of human drivers with the AV's decision-making, thereby increasing driving behaviors and ultimately safety.

General Assessment
While the paper addresses a critical issue—interpretable AI for safety-critical systems—the work exhibits substantial shortcomings in conceptual framing, methodological clarity, and evidentiary support. The claims of advancing driver mental models and improving alignment between humans and AVs are overinterpreted and insufficiently supported by the results. Below are the key limitations as observed through the Human-Machine Interaction lens:

Major Weaknesses
- Lack of Clarity and Logical Coherence. It is sometimes hard to follow the authors rationale, and the general approached used. The paper lacks a clear explanation of how CW-Net aligns human mental models with AV decision-making. The relationship between the AV's explanations and the driver's understanding is described in vague terms, without demonstrating causal links. The rationale for focusing on only three scenarios (unexpected stopping, phantom braking, and cyclist detection) is absent, despite the model being trained on a much broader dataset. This raises concerns about cherry-picking favorable situation and the usefulness of the approach in a variety of more frequent situations.

- Absence of Direct Measurement of Mental Models. The study does not include a direct or validated measure of changes in driver mental models. Instead, it relies on indirect proxies such as behavioral adjustments (from a single participant?) and subjective ratings from an online user study.

- Insufficient Theoretical Framing. The paper's framing highlights the opacity of deep neural networks but does not engage with prominent theoretical models of transparency and explainability. No clear reference is made to drivers' representations of the situation, even in a pragmatic fashion such as Situation Awareness assessment.

- Weakness of the Confirmation Study. The so-called "confirmation study" relies on an online survey where participants view pre-recorded videos and provide subjective rating. This approach is far removed from the operational context of AVs and fails to replicate the real-world cognitive and emotional demands of driving. It undermines the validity of the findings regarding improved driver understanding. This is surprising as the authors underlined the benefit of the real driving experiment also reported as compared to simulated studies.

Overinterpretation of Results
The authors make sweeping claims about the impact of CW-Net on driver behavior and safety, which are not substantiated by the data. In the abstract, the authors state: "We deploy CW-Net on a real self-driving car and show that the resulting

explanations refine the human driver's mental model of the car, allowing them to better predict its behavior and adjust their own behavior accordingly." This assertion is not supported by any rigorous evaluation of driver mental models or substantial behavioral adaptation in real-world conditions. The evidence presented—three handpicked scenarios and a limited online study—is insufficient to draw such conclusions.

Overall, the study centers on technological solution without adequately considering the human factors involved in human-AV interaction. It presumes that providing explanations will inherently lead to safer driving behaviors, overlooking the complexity and the variability among users.

(Remarks on code availability)

Referee #2

(Remarks to the Author)
This paper introduces the concept-wrapper network (CW-Net), a novel approach to explaining the motion planning component of autonomous driving via intelligible/human-interpretable concepts. The architecture, in its essence, extends the authors' recent study on interpretable-by-design
deep reinforcement learning with prototype-wrapper networks (PW-Nets) (ICLR 2023), grounded on concept bottleneck models. CW-Net is tested by providing explanations in three scenarios, as case-based reasoning, where an autonomous vehicle 1) gets close to another vehicle, 2) approaches a stopped vehicle, and 3) gets closer to a cyclist. The authors conduct both a simulation study and further deploy their architecture to a real autonomous vehicle. The empirical results and validation of results with human judgment show that CW-Net is a strong, well-grounded, and human-interpretable explainability approach to explaining real-time motion planning of an autonomous vehicle and helps a safety-driver to have more informed predictions about an autonomous vehicle's decisions, enabling a human driver to adjust their own behavior, particularly for takeover situations.

Overall, I found this paper very worthwhile as I think this is one of the rare studies leveraging concept bottleneck models for grounding explanations to humans' explanation needs with three concrete situations. My overall recommendation at this point is to accept the paper with minor revisions, as there are still a few points the paper needs to address. I list them as follows:

1. Summary of the key results: Figure 3 indicates the key findings of empirical results regarding the experiment and human evaluation. The delivery of results is understandable, as Figure 3 reports explanation effect, global analysis, and human evaluation.
2. Originality and significance: This paper is original and has a significant and valuable contribution to explainable autonomous driving via a new architecture, i.e., CW-Net, that provides explanations via concept bottleneck models. As far as I know, there have not been many studies on this topic before.
3. Data, methodology, clarity, and context: The CW-Net is a solid approach as its goal is to present human-interpretable explanations to humans (i.e., a safety driver in this case) in real time. The paper has also been written very well and is legible to a reader.
4. Statistical validation: The authors have leveraged the Mann–Whitney U test and two-sided T-test to validate the judgment of 120 people. The statistical findings reflect that explanations, indeed, support the alignment of the mental model of the target population.
5. Conclusions: Supports findings appropriately but can still be improved further, which I show below with details.

Based on these points, I would recommend the authors fix the following minor comments/address questions, which are about clarification, adding references, and fixing minor typos:

(I) Explanation conveyance is a challenging problem and needs to consider who they are targeted for. Furthermore, equally important, explanations should be intelligible for targeted interaction partners of various functional and cognitive capabilities and preferences (people with sight, hearing issues, age factors, etc.) to gain real-time situation awareness. I'm not sure if the user study considers this aspect. If not, this nuance, as a rule of thumb, should be discussed briefly in the introduction. Refer to the following papers, which show the challenges with explanation delivery for various user categories in real-time:
[1] S. Arfini, P. Bellani, A. Picardi, M. Yan, F. Fossa, and G. Caruso, "Design for Inclusivity in Driving Automation: Theoretical and Practical Challenges to Human-Machine Interactions and Interface Design," in Connected and Automated Vehicles: Integrating Engineering and Ethics. Springer, pp. 63-85, 2023.
[2] S. Atakishiyev, M. Salameh, R. Goebel. Incorporating Explanations into Human-Machine Interfaces for Trust and Situation Awareness in Autonomous Vehicles. In Proceedings of the 2024 IEEE 35th Intelligent Vehicles Symposium, Jeju, South Korea, pp. 2948-2955, 2024.

(II) How did you select the control group and experimental group? Was it done randomly?
(III) What simulator did you use? Please clarify and cite it.
(IV) Minor corrections:
Figure 3a: anther→ another
(V) Please provide future directions in the Conclusion section. How can CW-Net be extended for other scenarios, and what are the potential challenges with those cases?

If these comments are resolved, I believe the paper will be ready for publication. Thanks for your time and dedication to this research.

(Remarks on code availability)
I have reviewed the source code. It contains a README file with information for DEMO purposes and the modular code of CW-Net, but details on the System Requirements and Installation Guide are still missing. Therefore, the authors still need to improve the README file.

Referee #3

(Remarks to the Author)
The paper effectively highlights the need for explainability in AVs.

The proposed CV-Net is not aimed to improve the accuracy of the AV motion planner but to add interpretability and improve a more similar motion model to the human mental model.
Real-world implementation is considered using a semi-automated vehicle. However, only simple scenarios in a designated private lot are evaluated.

• While the manuscript emphasises a valid concern of interoperability in autonomous vehicles and provides some references on simulation and previous works of the same authors, it suffers from an in-depth literature review about the other research works done on the interpretability of AVs. Some of the common methods such as SHAP and LIME in XAI can be reviewed as well.

• The proposed model CV-Net adds explainability to existing black-box planners by using interpretable concepts for humans such as "Close to another vehicle", "Approaching stopped vehicle", or “close to a bike”; however, there is no justification on how and why these concepts are selected? Obviously, these three concepts cannot cover the AV decision-making domain.

• It is not clear how an AV can cope with other complex scenarios in multi-lane roads or intersections. The obtained results might be very different and non-acceptable.

• The manuscript expands the previous work of authors on PW-NET, however, no alternative models other than CW-Net are discussed or evaluated.

• The approach is only tested on the DriveIRL planner architecture. There is no evidence that CW-Net generalizes effectively to other planners or architectures used in AV systems.

• The proposed solution is more like an engineering setup with limited novelty. Obviously, the authors have tried to link some basic human decision-making with interpretability, however, I do not believe the model can be generalizable in complex traffic environments or other domains like autonomous drones, despite the claims made at the beginning of the manuscript.

• The authors need to provide further evidence and more experiments to support the validity, reliability, and robustness of the model.

• In the section conclusion, the authors might advise on how this work may influence AV regulatory standards or enhance the users' trust.

• The structure of the article can be significantly improved. Section 8 (Methodology), the most important part of the paper, comes after the section References. Then at the end of Section 8, there is another References section. And then after the second Reference a section for Appendix. I do not like the current structure of the manuscript with two sections for references and the methodology section out of the main context. This is very unusual and makes the manuscript hard to read/follow.

• There are a couple of complex terminologies which worth it further clarifications including "causally faithful explanations", and "mental model alignment".

(Remarks on code availability)

Version 1:

Reviewer comments:

Referee #2

(Remarks to the Author)
1. Summary of the key results: Figures 3 and Figure 15s indicate the key findings of experiments showing 1) drivers’ mental

alignment with presented explanations, and 2) the enhanced situation awareness was due to underlying improvements in user mental models of the AV as supported by the SAGAT study.

2. Originality and significance: This paper is original and has a significant and valuable contribution to explaining autonomous driving via a new architecture, i.e., CW-Net, that provides a method for explaining the behavior of ML planners by grounding their reasoning in human-interpretable concepts.

3. Data, methodology, clarity, and context: The CW-Net is a solid approach as its goal is to present human-interpretable explanations to humans (i.e., a safety driver in this case) in real time. The revised paper is well written, more careful in its wording, and easier for the reader to follow, reflecting the study's concrete research goals.

4. Statistical validation: The statistical findings reflect that AV explanations, indeed, support the alignment of the mental model of the target population.

5. Conclusions: Supports findings appropriately.

6. Suggested improvements: The quality of the paper has improved significantly after the first-round revision; I do not have any further suggestions.

7. References: References are appropriate.

8. Clarity and context: In the revised manuscript, the authors have significantly clarified their claims with more appropriate wording that presents evidence from their data/results, and have avoided talking beyond the data, which is good.

Recommendation: Accept for publication

Overall comments:

Dear Authors,

Thank you very much for revising the manuscript in response to the Reviewers' comments. Upon reviewing the revised manuscript, all my concerns have been addressed. Furthermore, I appreciate your detailed cover letter describing your efforts to improve the wording of the claims, including an analysis of the results within SAGAT and addressing of other concerns raised by other Reviewers. I do not have any further comments at this point and recommend acceptance of the manuscript for publication. Thanks for your dedication to this research.

Kind Regards,
The Reviewer

(Remarks on code availability)
The authors have improved the README file with a clearer description and meta instructions to enable registered users to reproduce their experiment and produce a GIF file. Pretty clear for reproducibility.

Referee #4

(Remarks to the Author)
This is a high-quality empirical study that successfully bridges the long-standing gap between theoretical XAI and real-world autonomous vehicle (AV) deployment. By demonstrating that concept-based explanations support mental-model formation across three layered environments—a real autonomous vehicle in deployment, a private test track, and public roads in Las Vegas—the authors provide qualitatively different evidence from the simulator- and MTurk-based studies that dominate the XAI literature. In my judgment, the revision faithfully addresses the previous concerns, and the paper provides rare and valuable evidence from a successful industry-academic partnership. Therefore, I recommend Accept with Minor Revisions subject to a few clarifying revisions described below.

A. Summary of the key results
The paper proposes a Concept-Wrapper Network (CW-Net), an architecture where the reward layer of a pre-trained IRL-based ML planner is replaced with a human-interpretable concept classifier and a new reward layer. The authors validate this approach through three independent and distinct layers of evidence, which constitutes the paper's principal strength. First, on the nuPlan benchmark, CW-Net exhibits less than 1% performance degradation relative to the black-box planner, satisfying the foundational requirement of "introducing interpretability without sacrificing driving performance." Second, in a semi-naturalistic private-track deployment, the explanations reveal that drivers' initial mental models were incorrect in all three tested situations (CLOSE/ASV/BIKE); counterfactual tests, such as vehicle repositioning and cone removal, further provide empirical support for the causally faithful nature of these explanations. Third, a large-scale follow-up simulator study (experts N=9, non-experts N=30) and an online SAGAT study (N=100) consistently yield large-to-medium effect sizes for "surprising events"—specifically in perception (d=1.29), comprehension (d=0.996), and projection (d=0.606)—while demonstrating no adverse effects during unsurprising events. This converging evidence across multiple environments effectively demonstrates the utility of CW-Net in supporting human situational awareness.

B. Originality and significance
The novelty of this work lies not in the methodology itself but in the strategic framing of the research question: the empirical demonstration of XAI utility within a real-world deployment context. Over the past decade, the XAI field has produced hundreds of explanation-method papers, yet studies that directly confront the repeatedly noted "lack of effectiveness validation in front of actual practitioners"—especially in safety-critical domains like automated driving—are exceedingly rare. Through a successful partnership with an industry partner, the authors secured qualitatively different evidence that would be difficult for an academic research group to reproduce on its own. By bridging the gap between theoretical XAI and operational deployment, this work provides a valuable baseline and a significant data point for the research community to reference, distinguishing itself from the simulator-only studies that dominate the current literature.

C. Data & methodology: validity of approach, quality of data
The methodology is a significant strength of this work, characterized by a canonical mixed-methods approach that reappraises the value of field data. While one could point to the three anecdotes from safety drivers in the initial field test as a potential weakness, this is an established research method within the context of semi-naturalistic field observation, and numerous prior studies have successfully reported results using similar approaches. In the field of HCI and automated driving, it is common to rely on controlled simulations for situation awareness (SA) research. However, the authors complement and extend this tradition by generating experimental stimuli directly from real-world public road data. By grounding their findings in actual environmental complexity rather than purely synthetic scenarios, the authors provide a superior level of ecological validity. This design, further bolstered by a subsequent N=100+ online validation, effectively addresses concerns regarding external validity and cannot be dismissed on the basis of a simple frequentist critique of sample size. The resulting insights are, therefore, highly relevant to the practical challenges of real-world AV deployment.

D. Appropriate use of statistics and treatment of uncertainties
The authors appropriately use linear mixed-effects models to control for participant-level individual differences, report effect sizes (Cohen's d), and employ both non-parametric (Mann–Whitney U) and parametric (t) tests while applying Bonferroni correction for multiple comparisons. This level of analysis is commendable; however, several areas require improvement for technical completeness and clarity.
1. Reporting 95% confidence intervals (CIs) alongside every effect size would make the underlying uncertainty significantly easier for readers to grasp visually and interpret.
2. The "LLM-as-a-judge" component—a critical part of the current qualitative evaluation—would be considerably strengthened by releasing the specific prompts used and by conducting a simple inter-rater reliability check. Specifically, the authors should report Cohen's κ after recoding a sample (e.g., N=50) of the responses with one or two human coders to validate the LLM's assessments.
3. The authors should report the internal consistency (Cronbach's α) for the four SAGAT perception items. This is necessary to statistically justify the use of a composite score for perception and ensure the robustness of the situational awareness assessment.

E. Conclusions: robustness, validity, reliability
The current conclusions, after revision, are well-grounded and considerably more measured in tone than the previous version. The authors have faithfully addressed previous concerns by removing overreaching direct claims about AI safety and tightly confining the scope to "mental-model goodness" and "situational awareness." Furthermore, the inclusion of discussions regarding regulation and trust, in line with Reviewer 3’s suggestion, adds significant pragmatic value and credibility to the overall work. The robustness of the conclusions is now well-supported by the converging evidence across the three validation layers.

F. Suggested improvements: experiments, data for possible revision
In my judgment, the converging evidence presented is sufficient, and no additional experiments or re-analyses are required. However, for the sake of scientific accuracy and to further enhance the impact of the manuscript, I suggest the following minor improvements:

1. While the authors discuss the Las Vegas public road data, I recommend explicitly mentioning in the Discussion section how this approach addresses the limitations of purely synthetic or simplified simulator scenarios often found in prior situational awareness (SA) research. Emphasizing this transition from "controlled simulation" to "real-world environmental complexity" would better showcase the paper's ecological validity and its practical relevance to the AV industry.
2. In the phrase "establishes a general pathway to interpretability for autonomous agents," I recommend softening "general" to "promising" or "deployment-validated" to more accurately reflect the specific context of the study.
3. The assertion "allowing them to better predict its behavior" should be qualified. Since the reported improvements are primarily observed during unexpected events, adding "particularly in surprising situations" would accurately reflect the experimental results and maintain the scientific integrity of the findings.

G. References: appropriate credit to previous work?
The authors have correctly cited established XAI and HCI frameworks, including Hoffman et al. and the SAGAT methodology. To provide a more comprehensive context for the Nature readership, I suggest the following additions:
1. Distinction from Post-hoc Methods: Briefly mention the distinction between CW-Net (interpretable-by-design) and common post-hoc explanation methods such as LIME or SHAP within the Methods or Related Work section. This would clarify why an intrinsic bottleneck approach like CW-Net is particularly suited for safety-critical autonomous agents compared to additive or perturbative explanations.
2. Contrast with Simulator-based SA Research: I recommend citing a few benchmark studies that have historically utilized

SAGAT within purely simulated environments (e.g., standard driving simulator studies found in venues like AutoUI or HFES). By explicitly referencing these simulator-based precedents, the authors can better highlight how their transition to real-world public road data addresses long-standing gaps in ecological validity. This contrast will emphasize that the current work is not merely another SAGAT application, but a significant methodological step forward in validating XAI through real-world environmental complexity.

H. Clarity and context: lucidity of abstract/summary, appropriateness of abstract, introduction and conclusions
The manuscript is exceptionally lucid and well-structured, effectively bridging the "grand challenge" of translating theoretical XAI into operational autonomous vehicle deployment. The Abstract and Introduction provide the necessary context for a broad scientific readership, successfully articulating the importance of human-centric validation in safety-critical AI systems. The logical progression—moving from nuPlan benchmarks to semi-naturalistic private-track tests, and finally to large-scale online validation using real-world public road data—is presented with remarkable clarity. This coherent storytelling makes the complex integration of deep learning, human factors, and field engineering highly accessible and compelling. The conclusions are appropriately drawn from the results, providing a clear and significant roadmap for future research in human–autonomous systems interaction.

(Remarks on code availability)
I accessed the provided source code via the Code Ocean link to verify the reproducibility of the CW-Net implementation. While the code itself is well-organized and modular, I encountered significant technical hurdles in executing the application within the Code Ocean environment.

Referee #5

(Remarks to the Author)
I co-reviewed this manuscript with one of the reviewers who provided the listed reports.

(Remarks on code availability)

Version 2:

Reviewer comments:

Referee #4

(Remarks to the Author)
I would like to thank the authors for their exceptional diligence and thoroughness in addressing all of my previous concerns. The revised manuscript and the detailed response package demonstrate a rigorous commitment to scientific accuracy and technical completeness.

A. Summary of the key results
The authors propose a Concept-Wrapper Network (CW-Net) that replaces the reward layer of a pre-trained IRL planner with a human-interpretable concept classifier. As another reviewer previously noted, the revised manuscript has become significantly well-written, more careful in its wording, and highly accessible to the reader.

B. Originality and significance
The work provides a highly original and significant contribution by bridging the gap between theoretical XAI and real-world operational complexity. securing qualitatively different evidence through a successful industry-academic partnership sets a valuable baseline for the community, distinguishing this paper from the simulator-only studies that dominate the current literature.

C. Data & methodology: validity of approach, quality of data, quality of presentation
The methodology is exceptionally robust. The multi-layered evaluation framework—spanning nuPlan benchmarks, semi-naturalistic private-track tests, and large-scale online validation using real-world public road data from Las Vegas—provides an outstanding level of ecological validity by grounding the findings in actual environmental complexity.

D. Appropriate use of statistics and treatment of uncertainties
The authors have fully addressed my technical concerns. The inclusion of 95% confidence intervals (Table S6) and the new Supplemental Methods containing the LLM-as-a-judge prompts along with the inter-rater reliability check (N=54) significantly strengthen the qualitative framework. Furthermore, reporting both the conventional Cronbach's alpha (0.754) and the ordinal alpha (0.929) for the SAGAT items completely satisfies the statistical justification for the composite score, effectively handling the ceiling effects in binary items.

E. Conclusions: robustness, validity, reliability
The conclusions are well-grounded, reliable, and measured. The authors have appropriately qualified their assertions (e.g., specifying improvements "particularly in surprising situations" and shifting to "deployment-validated"). The explicit discussion on transitioning from controlled simulation to real-world environmental complexity clearly elevates the paper's ecological validity and scientific integrity.

F. Suggested improvements: experiments, data for possible revision
In my judgment, the converging evidence presented across the three validation layers is sufficient. No further experiments or data re-analyses are required.

G. References: appropriate credit to previous work?
The references are appropriate and comprehensive. The added distinctions between CW-Net (interpretable-by-design) and post-hoc methods (LIME/SHAP), alongside proper context regarding simulator-based SAGAT precedents (e.g., AutoUI/HFES), provide excellent clarity and context for the Nature readership.

H. Clarity and context: lucidity of abstract/summary, appropriateness of abstract, introduction and conclusions
The manuscript is exceptionally lucid and well-structured. The logical progression from nuPlan benchmarks to real-world public road data is presented with remarkable clarity. The abstract and introduction successfully articulate the importance of human-centric validation in safety-critical AI systems, and the conclusions are appropriately drawn from the results.

I have no further comments or requests. I strongly recommend the manuscript for immediate acceptance and publication. Congratulations to the authors on this exemplary work.

(Remarks on code availability)
I appreciate that the authors made the Code Ocean capsule fully deterministic in response to my previous feedback. The capsule now converges reliably and the broader results are successfully reproduced in the toy domain by simply pressing Run. The code is well-organized, modular, and serves as a highly usable resource for the community.

Referee #5

(Remarks to the Author)
I co-reviewed this manuscript with one of the reviewers who provided the listed reports.

(Remarks on code availability)

Open Access This Peer Review File is licensed under a Creative Commons Attribution 4.0 International License, which permits use, sharing, adaptation, distribution and reproduction in any medium or format, as long as you give appropriate credit to the original author(s) and the source, provide a link to the Creative Commons license, and indicate if changes were made.
In cases where reviewers are anonymous, credit should be given to 'Anonymous Referee' and the source.
The images or other third party material in this Peer Review File are included in the article's Creative Commons license, unless indicated otherwise in a credit line to the material. If material is not included in the article's Creative Commons license and your intended use is not permitted by statutory regulation or exceeds the permitted use, you will need to obtain permission directly from the copyright holder.
To view a copy of this license, visit https://creativecommons.org/licenses/by/4.0/

Editorial Remarks

> *[Reframe] ...the message that more accurately reflects what is being empirically tested. ie if you are not explicitly testing safety reduction then temper these claims. Similarly, more work needs to be done in situating your working definition of explanation in the broader human-computer interaction literature (reports 1&2). This may require additional experiments to justify certain design choices or claims made in this regard.*

Thank you for this, we have addressed these points by doing the following:

- We have removed any direct claims we are testing AI safety, it is still mentioned in Section 5.3 as a descriptive term, but we only claim to test mental model goodness and situational awareness in the experiments (although of course these do impact AI safety [2]).
- We have clearly worded our definition of "explanation" to be in line with Gunning & Aha [4] as *'AI systems that can explain their rationale to a human user, characterize their strengths and weaknesses, and convey an understanding of how they will behave in the future'* in Section 3's first paragraph.
- We situate our evaluation within the broader HCI literature using the mental model tests suggested by Hoffman et al. [3], and The *Situation Awareness Global Assessment Technique* (SAGAT) [2] tailored for Explainable AI (XAI) by Sannerman & Shah [1].

All this did indeed entail many additional experiments, which we discuss next.

> *More generally, reviewers raised concerns about both the lack of realism of the empirical setting and the lack of complexity of the driving scenarios. I can imagine that improving upon both the 'realism' and complexity may be challenging for most labs, so this is something we can keep in mind when assessing editorially. However, it is clear that more work needs to be done in order to convince reviewers that the insights of this approach have broad relevance in AV safety generally. I wonder whether there is a way to link results from complex driving scenarios with humans in simulated environments with the more simple but 'real world' driving setup here?*

Thank you for understanding and appreciating the challenge involved with this type of research, we appreciate your suggestion and were inspired by it to do the following:

- We deployed the AV in *manual mode* (i.e., no self driving—as the AI planner is highly experimental [redacted]) around Las Vegas with several variants of CW-Net running in the background for a few hours to collect data in more realistic and complex environments, we refer to these new data as the "public roads" data, and our prior data last year as the "private track" data.
- We labelled the public roads data to find all scenarios which paralleled those from the private track data in that (1) the concept activation explanations were similar, (2) the AI planner behaved similarly, and (3) the human driver drove similarly to what the AV would have done if in *full self-driving* mode.
- With this we were able to quadruple the size of our materials set for user testing, and successfully link the prior private track data with the new public roads data.

- For these user tests, we first ran the mental model tests of Hoffman et al. [3] online with additional drivers at Motional (N=9) and a larger pool of non-experts (N=30) using the private track data, and showed a direct relationship between mental model goodness and performance metrics such as *counterfactual projection*.
- Then, following the suggestion of Hoffman et al. [3], we linked these results with the public roads data in a larger online SAGAT study [1, 2] (N=100) using these performance metrics as a proxy for mental model goodness to repeat the results on a larger scale and show a pragmatic usage of the improved mental model quality (see rebuttal Figures 1 and 2 below). Constructing the evaluation like this enabled us to link the private track data last year with the new public roads data while demonstrating causal links between CW-Net explanations and mental model quality to address issues related to the HCI framing and 'realism' concerns of the work.

Again, we appreciate your understanding of the complexity and challenges of this kind of research, we hope this has adequately addressed concerns regarding the study's realism and HCI framing.

Reviewer 1

Many thanks for the thoughtful review, we greatly appreciate the human-machine interaction viewpoint as it has massively improved the manuscript, we now iterate and respond to your comments and concerns.

> *While the paper addresses a critical issue—interpretable AI for safety-critical systems—the work exhibits substantial shortcomings in conceptual framing, methodological clarity, and evidentiary support. The claims of advancing driver mental models and improving alignment between humans and AVs are overinterpreted and insufficiently supported by the results.*

Thank you for acknowledging our work addresses a critical issue, we iterate all these concerns in the subsequent replies below, but to summarise, we situate our evaluation within the broader HCI and human factors literature using the mental model tests suggested by Hoffman et al. [3] and the *Situation Awareness Global Assessment Technique* (SAGAT) [2] tailored for Explainable AI (XAI) by Sannerman & Shah [1] to make substantiated claims regarding mental model goodness. See our rebuttal Figures 1 and 2 below, both of which outline our new evaluation pipeline and give an example of the logical flow.

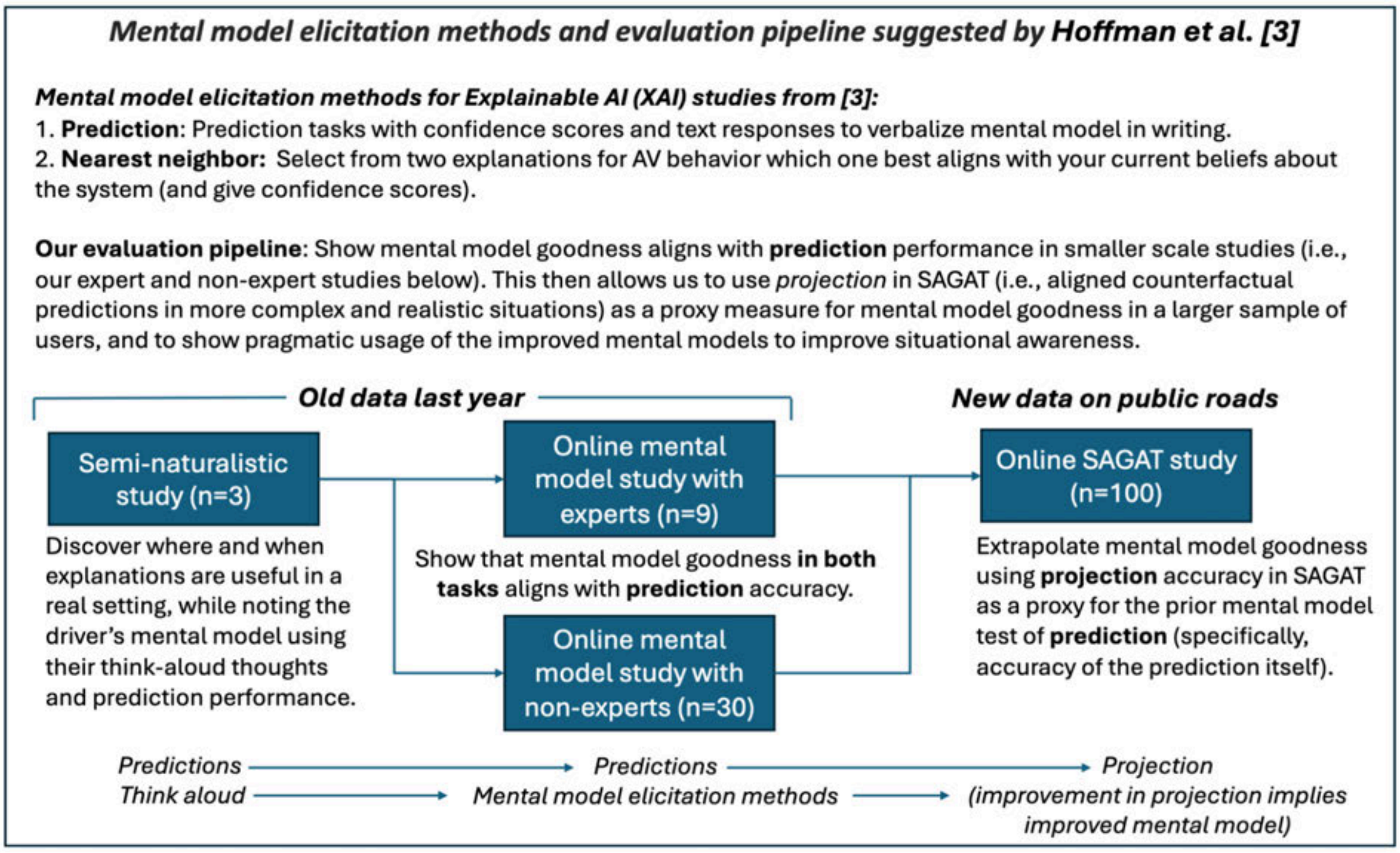

Figure 1: Overview of our evaluation methodology and study design suggested by Hoffman et al. [3] for Explainable AI (XAI) specifically. After collecting scenarios from a semi-naturalistic deployment setting and noting the driver's mental model and performance, we help verify the robustness of these findings in a larger online study with both experts and non-experts. After showing that mental model goodness and performance metrics align, we followed the suggestion by Hoffman et al. [3] and used the same performance metric (i.e., *projection* in SAGAT) as a proxy to elicit mental model goodness in a larger study with new data from complex scenarios on public roads. We can make this link because *projection* in our SAGAT study mirrors the prediction tasks themselves within the mental model elicitation method '**Prediction**' in our studies (see example below).

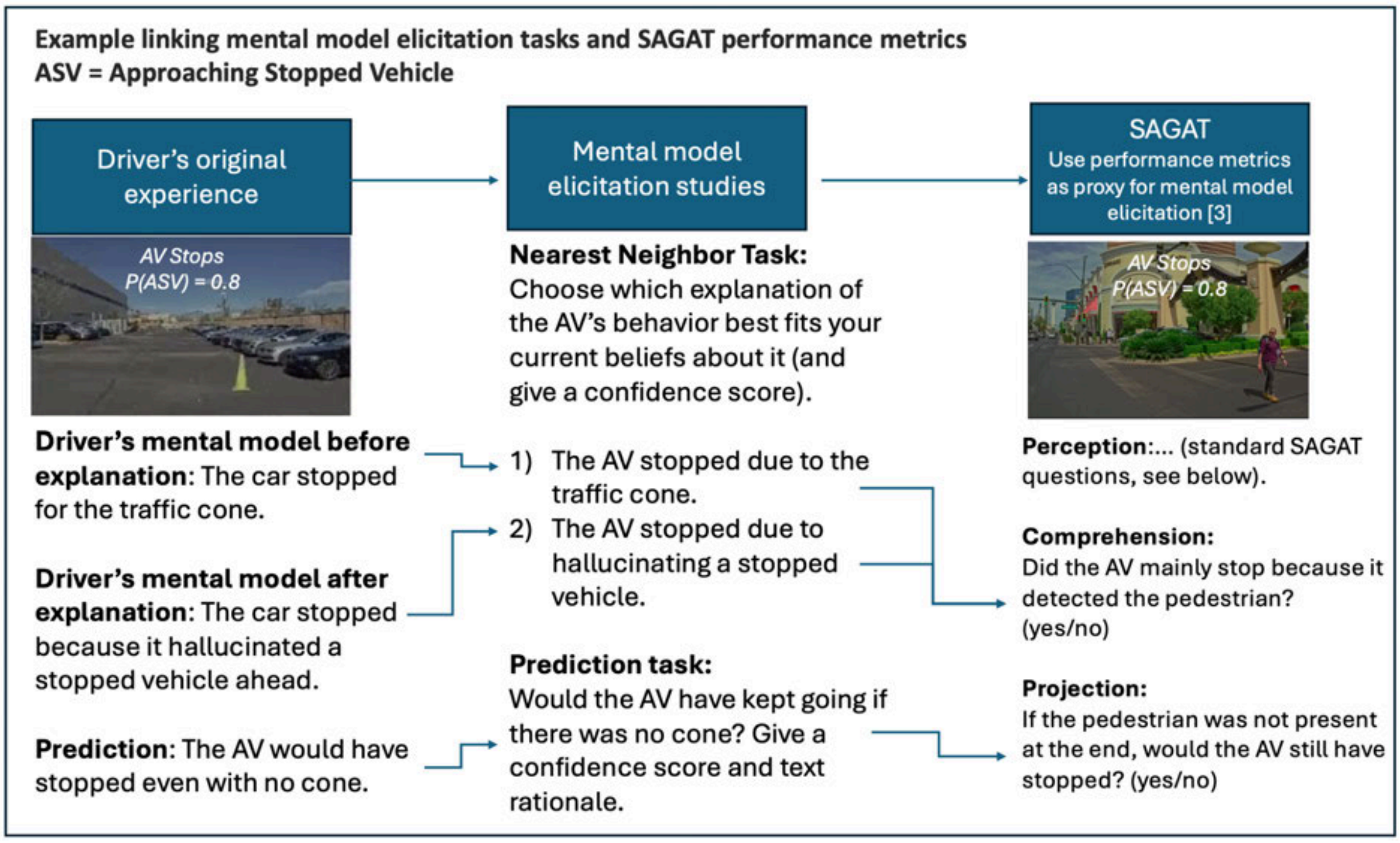

Figure 2: Overview of how we linked the driver's mental model progression last year to the new online tests with drivers and non-experts, and finally the SAGAT study to demonstrate causal links between CW-Net explanations and mental models. Note, although "Projection" in SAGAT is a valid proxy metric for the prediction task within the mental model elicitation task "Prediction", "Comprehension" in SAGAT is not strictly equivalent to the "Nearest Neighbor" (NN) mental model elicitation task, but we discuss the results for additional context. Specifically, the NN task asks if explanation A or B is closer to your current beliefs, whilst SAGAT "Comprehension" asks if e.g. explanation A is right/wrong.

Revision: We added the two figures to the supplement, and discussed this more at the beginning of Sections 5 and 6.

> ***Lack of Clarity and Logical Coherence***. *It is sometimes hard to follow the author's rationale, and the general approach used. The paper lacks a clear explanation of how CW-Net aligns human mental models with AV decision-making. The relationship between the AV's explanations and the driver's understanding is described in vague terms, without demonstrating causal links…*

As we outlined in our renewed study design in the rebuttal Figures 1 and 2 above, we now have a vastly expanded evaluation over last year's manuscript based on the reviewer's suggestions and insights. To elaborate, the logical flow is now as follows:

1. We ran a semi-naturalistic study with real safety drivers in their day to day job in order to understand if explainable AI—and more specifically CW-Net—was useful in an authentic setting. During this time, the researcher only dictated high-level plans for the day (e.g., *"drive around the private track again"*, *"test stopping ability for cyclists"* etc.). These tests lasted 4 days and encompassed 3 different drivers and 3 different support engineers.
2. In line with the cognitive science literature, we found the explanations were most sought during "surprising events" [10], at which point we noted the driver's think-aloud thoughts,

and their ability to project the AV's behaviour into the future after explanation exposure. All of which was compared against a ground truth obtained either through counterfactual tests immediately during deployment, and/or subsequent post-hoc analysis of the data. Importantly, we did *not* prompt the driver to do any of this.

3. With these scenarios at hand, we showed the videos to 9 other drivers/engineers at Motional familiar with the AV and test courses in the original data to see if their mental models progressed similarly. The two tests we ran were taken from Hoffman et al. [3] and are:
    a. **Nearest neighbour (NN)**: We presented subjects with two explanations for the surprising event (i.e., the original driver's think-aloud mental model before and after explanation exposure), and asked them which explanation best fitted their current beliefs about the AV, and to give a confidence score.
    b. **Prediction**: Subjects were asked to make the same counterfactual prediction the original driver made, with a confidence score, and text rational to probe their mental models of why they made the prediction.
4. Since AVs at Motional have progressed substantially since the original tests and we observed bias in their text responses giving weight to this (despite being asked not to do so), we again ran this study with 30 non-experts sampled from Prolific.com to see how non-biased users react. All users were paid far above the normal rate to maximize their engagement. Both studies indicated alignment between mental model quality and counterfactual prediction performance, indicating the latter may be used as a proxy measure for the former (see supplement Figures S4-S9).
5. Then, following the suggestion by Hoffman et al. [3] we extrapolated the counterfactual prediction performance metric into another larger study to use it as a proxy for mental model goodness on a larger scale. At this point, we took the reviewer's suggestion and used the SAGAT framework because it is quite pragmatic, known to be influenced by a user's mental model [2, 9], and its *projection* test can be used as a direct substitution for the 'prediction' mental model elicitation task (specifically, the prediction itself, not the text response). As an aside, SAGAT 'comprehension' also serves as a reasonable (but less valid) proxy for the 'nearest neighbor' mental model elicitation task. All this allowed us to make a causal link between CW-Net's explanations and user mental models as the reviewer asked for.

Revision: We explain this at the beginning of Sections 5 and 6, as well as adding the prior two figures above to the manuscript (now Figures S2 S3 in the supplement).

> ***Insufficient Theoretical Framing****. The paper's framing highlights the opacity of deep neural networks but does not engage with prominent theoretical models of transparency and explainability. No clear reference is made to drivers' representations of the situation, even in a pragmatic fashion such as Situation Awareness assessment…*

Our original rationale was to follow a prominent evaluation methodology in XAI called 'model simulation' (which is well established by seminal papers [15]) when we evaluated the driver's counterfactual projections (note this is linked to mental model quality [2]). As discussed above, to address this concern we engaged with the work of Hoffman et al. [3] and the *Situation Awareness Global Assessment Technique* (SAGAT) tailored for XAI by Sannerman & Shah [1,2] to make substantiated claims regarding mental model goodness of the drivers and their representation of the situation.

Revision: We cite and discuss these works throughout Section 6 to help frame the work better.

> ***Absence of Direct Measurement of Mental Models****. The study does not include a direct or validated measure of changes in driver mental models. Instead, it relies on indirect proxies such as behavioral adjustments (from a single participant?) and subjective ratings from an online user study…*

As our initial tests with the drivers (n=3) were in a semi-naturalistic setting, our measures necessarily revolved around observational data. This involved waiting for the "surprising" situations noted in the paper, while recording the driver's think-aloud thoughts and counterfactual predictions (which are reasonable methods for mental model elicitation in XAI [3]). This carried the enormous advantage of studying explanations "in the wild" rather than a pre-designed experimental setup, which was again necessary as our core research question is whether or not explanations help in natural settings between humans and AVs. However we understand the limitations of such setups, and to strengthen these findings, as discussed above, we now strictly employ two rigid mental model elicitation tasks from Hoffman et al. [3] in our followup online studies, to elaborate more the tasks are:

- **Nearest Neighbour (NN)**: The users are shown the videos of the driver's "surprising" situations, and given two possible explanations for the AV's behaviour. The two explanations correspond to the original driver's mental model both before and after explanation exposure (as noted through their think-aloud thoughts). Users must pick which best aligns with their current beliefs about the AV, and give a confidence score to understand how their "distance" to this belief is changing; in case they did not flip the prediction, this allows us to understand how the mental model is trending (i.e., either improving, staying the same, or worsening).
- **Prediction**: As a second question, the users must (1) make the same counterfactual prediction as the original driver, (2) give a confidence score, and (3) explain their reasoning behind this prediction in as much detail as possible as a text response so we can ensure they are e.g. making a correct prediction for the right reasons.

The "nearest neighbor" data and confidence scores were used to bin users' mental models into three categories (worsened, no change, improved) and the text rationale for the "prediction" task was used to bin it into two (worsened or improved), see Section 6.1 in the revised manuscript (and Methods section) for details on this. To summarise, the data indicated a clear relationship between mental model goodness and predictive performance. Specifically, linear mixed-effects models confirmed that mental model improvements in the NN task correlated with prediction accuracy gains for both experts (β=2.02, p=0.02) and non-experts (β=9.86, $p < .001$), while improvements in the text rationale task also showed positive correlations (β=1.70, p=0.06 for experts; β=5.03, $p < .001$ for non-experts), indicating enhanced counterfactual prediction was driven by a more accurate underlying understanding of the system. Note larger p-values in the expert study are largely caused by a necessarily smaller sample size.

Moreover, the two mental model measures themselves were strongly aligned (and indeed aligned between experts/non-experts), indicating robustness of these findings (note this test was suggested by Hoffman et al. [3] because occasionally different measures may diverge, indicating issues with the study—see the paper's Figure S8—but ours aligned and thus indicated the study was sound). Taken as

a whole, all this allowed us to use similar prediction tasks as a proxy for mental model goodness in the subsequent SAGAT study, see Section 6 and supplement Figures S4-S9 for full details.

Revision: We completely redesigned our online simulation studies to directly measure mental models using established approaches in the recent XAI literature [3], added figures S4-9 in the paper, and rewrote Section 6 to reflect all this.

> ***Weakness of the Confirmation Study***. *The so-called "confirmation study" relies on an online survey where participants view pre-recorded videos and provide subjective ratings. This approach is far removed from the operational context of AVs and fails to replicate the real-world cognitive and emotional demands of driving. It undermines the validity of the findings regarding improved driver understanding. This is surprising as the authors underlined the benefit of the real driving experiment also reported as compared to simulated studies.*

Our main contribution is the fully self-driving tests last year in which we showed the first example of XAI being useful in a real deployment context with AVs; the online studies only serve to strengthen these findings. We argue the online video-based protocol functions as a valid proxy for the in-vehicle experience, because the drivers in the real-world study had limited cognitive and emotional demands. Cognitively, they did not execute manual takeovers during 'surprising' events, but rather remained in a state of passive monitoring identical to that required of the online viewers. Emotionally, as the drivers are quite accustomed with such testing, their emotional state is actually more complacent than stressed (aligning with the literature on the topic, e.g. see [18]), which again aligns with online users. Furthermore, previous research on simulation validity demonstrates that such video and simulator-based setups actually possess high *relative* validity [11, 12, 16]. Of course, users online will lack the extensive context the driver has, but the convergence of results between the real-world study last year and our new online studies presented in the current revision strongly suggest that the online protocol serves as a very good proxy.

Nevertheless, we agree that the online study could be further improved, so we did the following:

1. We deployed the AV on the Las Vegas Strip in 'manual mode' where the AI planner was running the background as the human drove for several hours in full control. Driving in 'manual mode' was necessary as our explainable planner is highly experimental [redacted]. This allowed us to collect data from the planner in more complex scenarios involving dozens of other cars, pedestrians, stop signs, traffic lights, crosswalks, and intersections etc.
2. These data were labelled to find scenarios which mimicked those discovered last year in the fully automated private track tests. To be labelled as such, they had to (1) similarly activate the relevant concept, (2) exhibit similar behavior to the AI planner in the analogous original scenarios (i.e., it had to be stuck beside another vehicle for `CLOSE`, brake suddenly for `ASV` whilst hallucinating a stopped vehicle ahead, and not brake for `BIKE`), and (3) the human driver had to closely mimic the AI planner so as to accurately capture what would happen in full self-driving when showing users the videos. We were able to label at least 2 such scenarios for each concept. Note that because there were no bikes encountered on the public

roads, we mimicked the setup with pedestrians, because the concept behaved similarly. We also replaced the traffic cone in the `ASV` tests with pedestrians as there were no cones encountered either.

3. We further labelled an equal number of “unsurprising” scenarios which had the same setup, but without the surprising aspect. For example, with `ASV`, instead of presenting users with scenarios where the AV hallucinated a stopped vehicle ahead, we showed them scenarios in which there was one present. This allowed us to observe the situational awareness of users in “surprising” and “unsurprising” events, to verify the explanation helped in the prior, and didn’t hurt in the latter.
4. We reran our online study with these new public roads scenarios to draw parallels to the private track driving scenarios collected last year (taking the editor’s suggestion). This allowed us to generalize our results beyond the private-track testing to more complex realistic scenarios.
5. These videos were generated to show two concept activations for the experimental group, and two placeholders for the control to balance cognitive load (which were just steering and speed).
6. Following SAGAT, users were asked 4 questions on *perception*, 1 on *comprehension*, and 1 on *projection.* This was designed to be a close reenactment of the driver’s experiences last year to draw a direct comparison and measure mental models using the pipeline discussed in the rebuttal Figures 1 and 2 above.

We explain all this in full detail in the Methods section and Section 6 of the main paper. Please see the figures below for examples of these new materials and the SAGAT study results. In short, the results showed the explanations significantly improved situational awareness during surprising events. Specifically, we observed large effect sizes for perception (d=1.29), comprehension (d=0.996), and a medium effect size for projection (d=0.606). Furthermore, analysis of unsurprising events confirmed that explanations introduced no adverse effects on situational awareness. Interestingly, we found converging results in the relative effect sizes of the mental model elicitation tasks (Nearest Neighbour and Prediction, 41.17% and 26.65% improvements) and the SAGAT surrogates (Comprehension and Projection, 20.26% and 12.78% improvements). The fact that the 'counterfactual' tasks (Prediction and Projection) consistently showed lower but proportionate improvement compared to the 'recognition' tasks (Nearest Neighbour and Comprehension) across both independent studies indicates that the SAGAT metrics are likely capturing the same underlying cognitive constructs, validating them further as reasonable mental model surrogate measures.

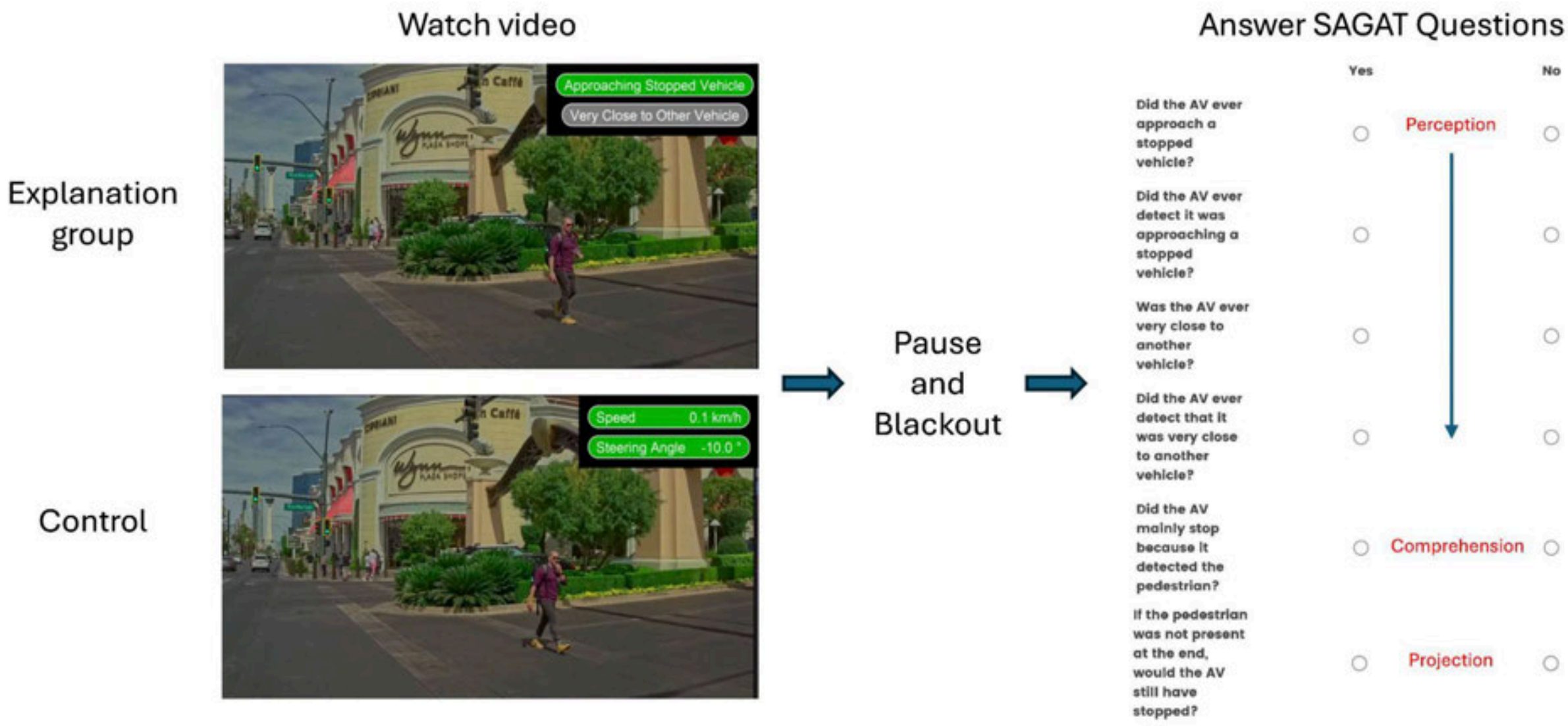

Figure 4: Public roads material example for SAGAT: *New material example on the Las Vegas Strip: The "surprising" event mirrors the close-court tests from last year when the AV hallucinated a stopped vehicle ahead and braked for a traffic cone. After viewing the video in full, users experienced a "blackout" and were asked six questions, with the questions designed to mirror the private track tests last year with the safety drivers (particularly comprehension and projection).*

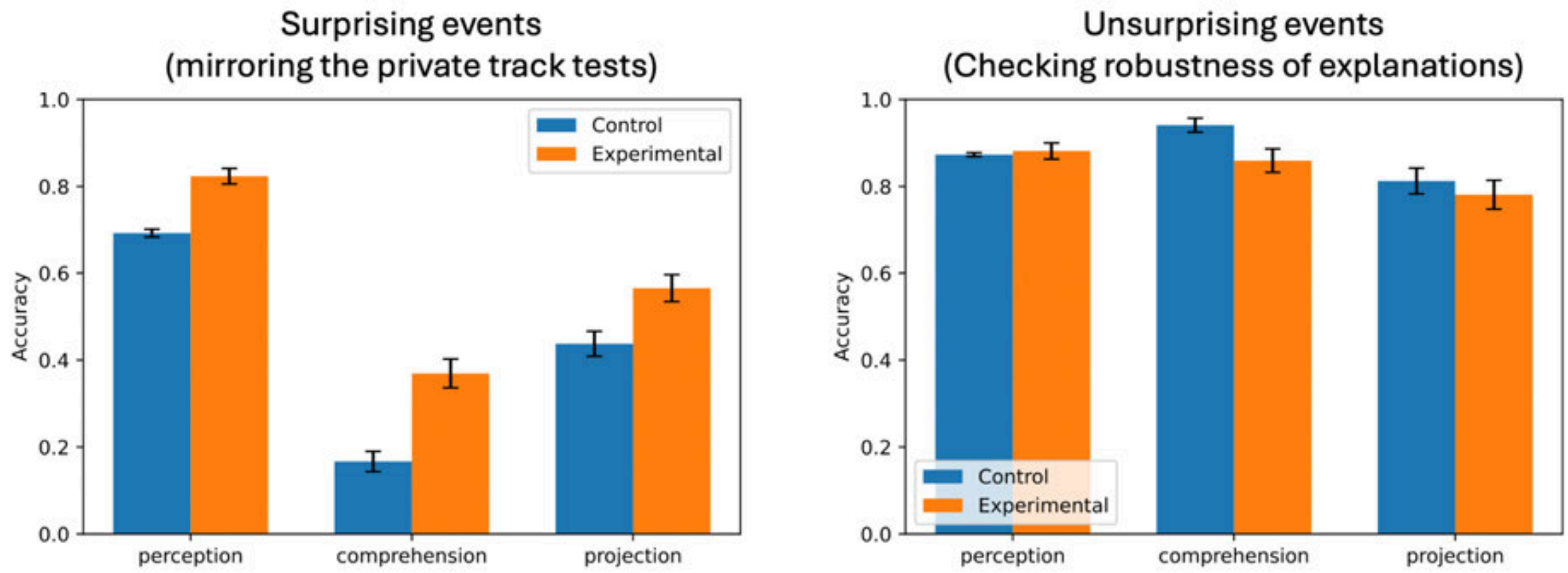

Figure 5: SAGAT results*: The results of the improved user study show significant improvements in situational awareness when "surprising" events occur in all dimensions of perception, comprehension, and projection, mirroring our results of the private track tests last year with drivers. In contrast, although comprehension trended downwards slightly in "unsurprising events" for the explanation group, this did not reach statistical significance after Bonferroni correction.*

Revision: We redeployed the AV on public roads to collect more realistic scenarios which mirror those discovered in the private track tests (on the editor's suggestion), showing CW-Net's effects on user mental models remains robust. In addition, we used SAGAT as a pragmatic approach to show the practical utility of these improvements in mental models (on the reviewer's suggestion), see Section 6.2 and supplement Figures S10-S15.

Possible cherry picking: *The rationale for focusing on only three scenarios (unexpected stopping, phantom braking, and cyclist detection) is absent, despite the model being trained on a much broader dataset. This raises concerns about cherry-picking favorable situations and the usefulness of the approach in a variety of more frequent situations.*

Thank you for raising this, we can explain the rationale in two parts:

1. ***Concept relevance and wider impact***: We mainly chose the three concepts because they deal with other road users, and are thus most relevant for safety broadly. For example, the first deployment used a dataset with concepts [`LEFT, RIGHT, STRAIGHT, STOP, SLOW, ASV, CLOSE, INTERSECTION`]. Of these, only `ASV` and `CLOSE` deal with other road users and benefit most from real-time feedback (note most—if not all—AV companies currently hardcode concepts such as 'intersection' or 'traffic light' on the map, and are thus not relevant to most companies' AI planners). The other deployment setup used concepts [`LEFT, RIGHT, STRAIGHT, STOP, SLOW, FAST, FOLLOWING, BIKE, PEDESTRIAN, INTERSECTION, TRAFFIC_LIGHT, STOP_SIGN`]. Of these, only `FOLLOWING`, `BIKE`, and `PEDESTRIAN` dealt with other road users. `FOLLOWING` required longer testing times due to the need for additional safety drivers interacting with the AV. We focused on `BIKE`, which was easier to test, although we would have observed similar results with `PEDESTRIAN` (as evidenced by the public road tests). There were no other datasets available to us at the time of testing.
2. ***Counterfactual testing***: The concepts were also easier to test counterfactual *projection* with (a.k.a., 'model simulation' [15]). For example, if the AV strongly detects `ASV`, `CLOSE`, or `BIKE`, it is expected it would likely stop (or at least slow down), which allows more straightforward testing. For concepts such as `STRAIGHT` this is not the case, as the AVs speed depends upon other factors besides the concept, such as obstacles on the road etc., which we may not have labels for.

As an aside, from a technical perspective, we also included additional miscellaneous concepts (e.g. the steering and speed) because it allowed us to expand the bottleneck in our model, which is better for performance. For example, at the most extreme, a CW-Net model with a single concept will have to traverse a bottleneck of a single neuron, and hence compress the model's representations into this single neuron, while also doing concept classification with the same neuron, which radically reduces performance. We overcame this by adding additional concepts which, although were not the focus of our tests, allowed an expanded concept bottleneck.

Revision: We now explicitly mention this at the end of the second paragraph of Section 5.

Overinterpretation of Results*: The authors make sweeping claims about the impact of CW-Net on driver behavior and safety, which are not substantiated by the data. In the abstract, the authors state: "We deploy CW-Net on a real self-driving car and show that the resulting explanations refine the human driver's mental model of the car, allowing them to better predict its behavior and adjust their own behavior accordingly." This assertion is not supported by any rigorous evaluation of driver mental models or substantial behavioral adaptation in real-world conditions. The evidence presented—three handpicked scenarios and a limited online study—is insufficient to draw such conclusions.*

Overall, the study centers on technological solutions without adequately considering the human factors involved in human-AV interaction. It presumes that providing explanations will inherently lead to safer driving behaviors, overlooking the complexity and the variability among users.

We thank you for this candid assessment. To revise, we took this very seriously and addressed concerns regarding overinterpretation, human factors, and scenario selection in the following ways:

- **Claims**: We carefully revised the abstract and text to remove claims regarding direct testing of safety reduction (although of course our results do impact AI safety [2]). We now strictly frame our contributions around improvements in "mental model goodness" and "situational awareness," safety is only mentioned as a descriptive term in Section 5.3.
- **Human factors and real-world concerns**: We moved beyond our semi-naturalistic study (and original online evaluation) to provide robustness with a rigorous quantitative pipeline involving 139 participants (9 experts, 30 non-experts, and 100 SAGAT participants), demonstrating alignment in mental models and performance metrics across tests. Furthermore, by collecting data from public roads we addressed your concerns over real-world conditions.
- **Concept selection**: We explained our rationale for focusing on the three scenarios by way of their relevance to current deployment conditions of AVs globally and our particular experimental constraints.
- **Theoretical HCI framing**: Lastly, by grounding our evaluation in the established theoretical frameworks of Hoffman et al. [3] and SAGAT [1, 2], we have addressed issues related to HCI framing.

We hope the reviewer now finds we have adequately considered the human factors involved in human-AV interaction.

Best regards,

The Authors

Reviewer 2

Many thanks for your review, it has helped us a lot to improve the work, we now respond to all your points one-by-one.

> *(I) Explanation conveyance is a challenging problem and needs to consider who they are targeted for. Furthermore, equally important, explanations should be intelligible for targeted interaction partners of various functional and cognitive capabilities and preferences (people with sight, hearing issues, age factors, etc.) to gain real-time situation awareness. I'm not sure if the user study considers this aspect. If not, this nuance, as a rule of thumb, should be discussed briefly in the introduction. Refer to the following papers, which show the challenges with explanation delivery for various user categories in real-time:*
> *[1] S. Arfini, P. Bellani, A. Picardi, M. Yan, F. Fossa, and G. Caruso, "Design for Inclusivity in Driving Automation: Theoretical and Practical Challenges to Human-Machine Interactions and Interface Design," in Connected and Automated Vehicles: Integrating Engineering and Ethics. Springer, pp. 63-85, 2023.*
> *[2] S. Atakishiyev, M. Salameh, R. Goebel. Incorporating Explanations into Human-Machine Interfaces for Trust and Situation Awareness in Autonomous Vehicles. In Proceedings of the 2024 IEEE 35th Intelligent Vehicles Symposium, Jeju, South Korea, pp. 2948-2955, 2024.*

Thank you for emphasising this important aspect, we completely agree and referenced the papers suggested. As an aside, we have also conducted a *situation awareness global assessment technique* (SAGAT) study in Section 6 to bolster our overall results. If the reviewer wishes they may view our responses to Reviewers 1 and 3 for more details on this.

Revision: We added these citations and briefly discussed this in the introduction.

> *(II) How did you select the control group and experimental group? Was it done randomly?*

Yes, we sampled the users using Prolific.com, which is known for its high-quality user base compared to other options like Amazon Mechanical Turk [8]. The application sampled random users from the United States who were between 18-80, native English speakers, and had an approval rating of 95%+ on Prolific. The last constraint specified was that there was an even split of male/female. In our new user tests (see Sections 5 and 6), we have taken the same approach and additionally paid prolific users above average to help ensure high-quality responses. We describe the studies in full detail in the Methods section.

> *(III) What simulator did you use? Please clarify and cite it.*

Revision: We used the nuPlan simulator, this is now cited in Section 4.

(IV) Minor corrections: Figure 3a: anther→ another

Revision: Done, thank you for pointing this out.

(V) Please provide future directions in the Conclusion section. How can CW-Net be extended for other scenarios, and what are the potential challenges with those cases?

Revision: Great idea, we have included this as the final sentence of the conclusion. In short, we believe the most promising direction for future research is to learn concepts in an unsupervised manner to assist with scalability, possibly with the usage of sparse autoencoders..

I have reviewed the source code. It contains a README file with information for DEMO purposes and the modular code of CW-Net, but details on the System Requirements and Installation Guide are still missing. Therefore, the authors still need to improve the README file.

Revision: Thank you for reviewing our code, as the code was run on CodeOcean which automates the process largely, we felt system requirements were less relevant. However this is important for when users export the code, we have fixed this, many thanks for pointing this out, it will enable easier reproducibility.

If these comments are resolved, I believe the paper will be ready for publication. Thanks for your time and dedication to this research.

We hope this has addressed your remaining concerns, many thanks for your help and for the kind words.

Best regards,

The Authors

Reviewer 3

We express our gratitude for the review, it has substantially improved the quality of our manuscript, we now respond to all your concerns.

> ***Realism Concern***: *Real-world implementation is considered using a semi-automated vehicle. However, only simple scenarios in a designated private lot are evaluated. ...*
> *It is not clear how an AV can cope with other complex scenarios in multi-lane roads or intersections. The obtained results might be very different and non-acceptable.*

We appreciate the reviewer noting this. Just to be clear, please note the autonomous vehicle (AV) used in all our original tests was 100% self-driving. There were instances in which the driver took over for safety reasons or if the planner ran into issues, and indeed the AV's emergency braking system did occasionally override the AI planner (still this did not involve human intervention), but all the data analysed in the original manuscript was from self-driving only, it was *not* 'semi-automated'.

We focused on private-track testing because the experimental nature of our black-box ML planner meant it could not be safely deployed on public roads with full autonomy. Indeed, even flagship AVs struggle with this and are only cleared to drive within 'operationally defined domains' where traffic lights, buildings, sidewalks etc. are frequently hard-coded, or even have safety drivers sitting in the AV [14]. It is also important to consider that as AVs are frequently refined and debugged in private tracks, explanations may prove the most useful there to help prevent accidents through debugging *before* they happen, so this formed a large focus of our work.

All this said, we understand your concern regarding more complex environments, and to address it we have done the following:

1. We deployed the AV around Las Vegas in 'manual mode' where the AI planner was running the background as the human drove for several hours in full control (distinct from the previous private track tests that were fully automated). **This allowed us to safely collect data** from the experimental planner in more complex scenarios involving dozens of other cars, pedestrians, intersections etc.
2. These data were labelled to find scenarios which connected to those discovered last year in the fully automated private track tests. To be labelled as such they had to (1) activate the relevant concept the same, (2) the AI planner had to select trajectories the same way in the same type of scenario (i.e., it had to be stuck beside another vehicle for `CLOSE`, brake suddenly for `ASV` whilst hallucinating a stopped vehicle ahead, and not stop for a `BIKE`), and (3) the human driver had to closely mimic the AI planner so as to accurately capture what would happen in full self-driving when showing users the videos online. We were able to label at least 2 such scenarios for each concept which naturally occurred. Note that because there were no bikes (or traffic cones) encountered on the public roads, we mimicked the setups with pedestrians (see rebuttal Figure 7).
3. We further collected an equal number of "unsurprising" scenarios which had the same setup, but without the "surprising" aspect. For example, with `ASV`, instead of presenting users with scenarios where the AV hallucinated a stopped vehicle ahead, we showed them scenarios in which there was one (see Figure 6 below), this allowed us to observe the situational

awareness of users in “surprising” and “unsurprising” events. The point of this was to verify the explanation helped in the prior, and didn’t hurt in the latter, thus helping to further demonstrate robustness of the explanations.

4. We reran our online study with these more realistic scenarios to draw parallels to the ‘real world’ driving scenarios collected last year (taking the editor’s suggestion). This allowed us to generalize our results safely beyond the private track testing to more complex public road scenarios as the reviewer asked for.
5. These videos were generated to show two concept activations for the experimental group, and two placeholders for the control (which were just steering and speed, see the rebuttal Figure 6 below).
6. Users were asked 6 questions using the *Situation Awareness Global Assessment Technique* (SAGAT) test [1], 4 for *perception*, 1 for *comprehension*, and 1 for *projection*, all of which were designed to mimic the driver’s situations last year, but again in more realistic, complex scenarios on public roads (see rebuttal Figures 6-8 below).

The results showed significant improvement in situational awareness for the explanation group (across all dimensions) compared to the control in surprising events, thus helping to answer the reviewer’s concern about how our model—and explanations—would deal with more complex scenarios. Specifically, we observed large effect sizes for perception (d=1.29), comprehension (d=0.996), and a medium effect size for projection (d=0.606). Please see the figures below and the main paper’s supplement Figures S10-15 for more. No significant differences were observed in unsurprising events, lending evidence the explanations do not hurt mental models or situational awareness in more typical driving scenarios.

If the reviewer wishes, they may view our response to Reviewer 1 (and the rebuttal Figures 1 and 2 above in particular) for additional details on our new HCI framing of this evaluation and we how these results were due to improved mental models in the participants (see supplement Figures S2-S9 and Section 6 for further details).

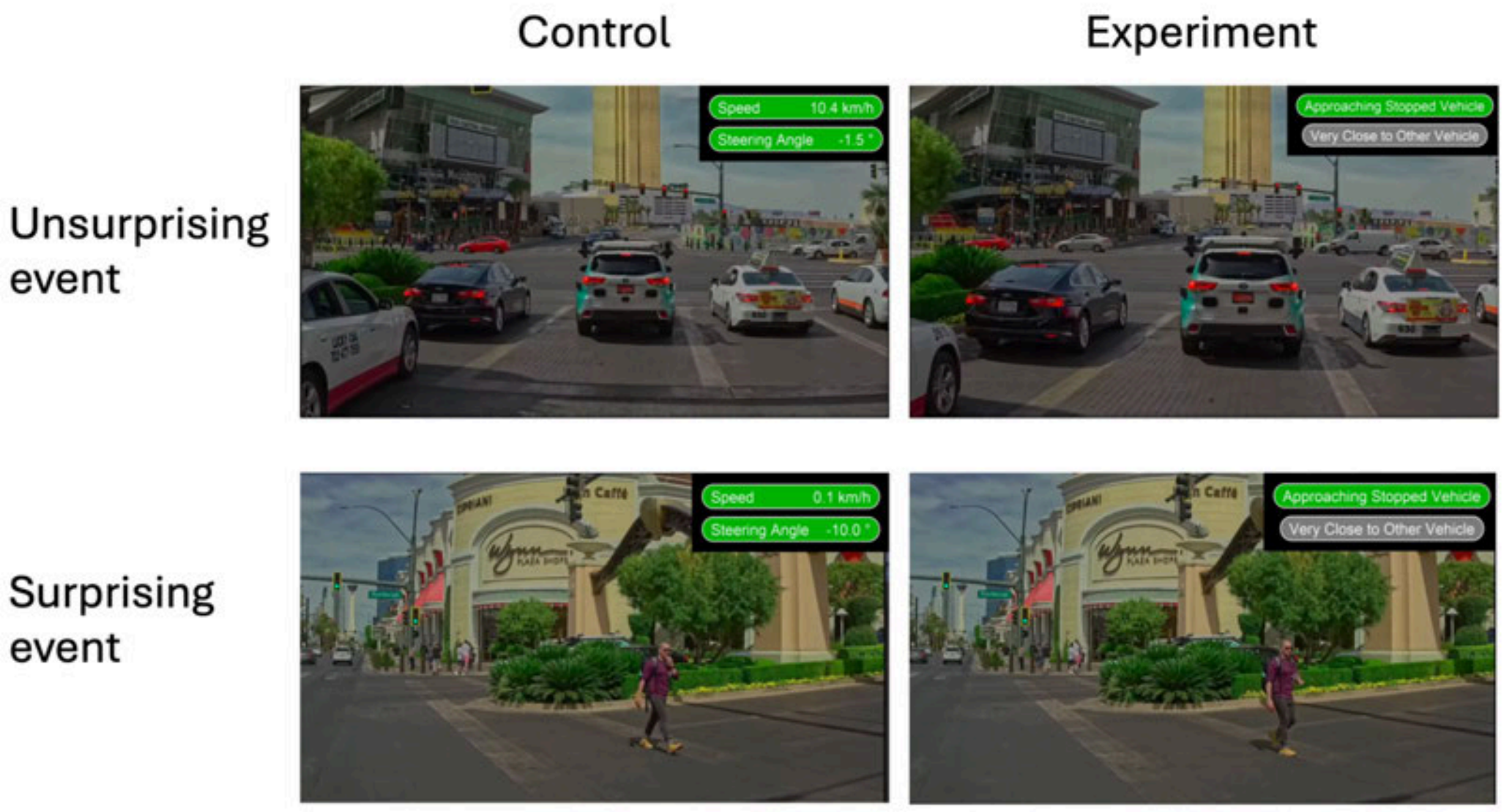

Figure 6: Comparing Control vs. Experiment and Surprising vs. Unsurprising events*: New material example on the Las Vegas Strip: The "surprising" event mirrors the private-track tests from last year when the AV appeared to stop for a traffic cone but in fact hallucinated a stopped vehicle ahead (but here we replace the traffic cone with a pedestrian). The “Unsurprising event” shows the AV and explanations behaving correctly to verify the explanations do not negatively impact user mental models.*

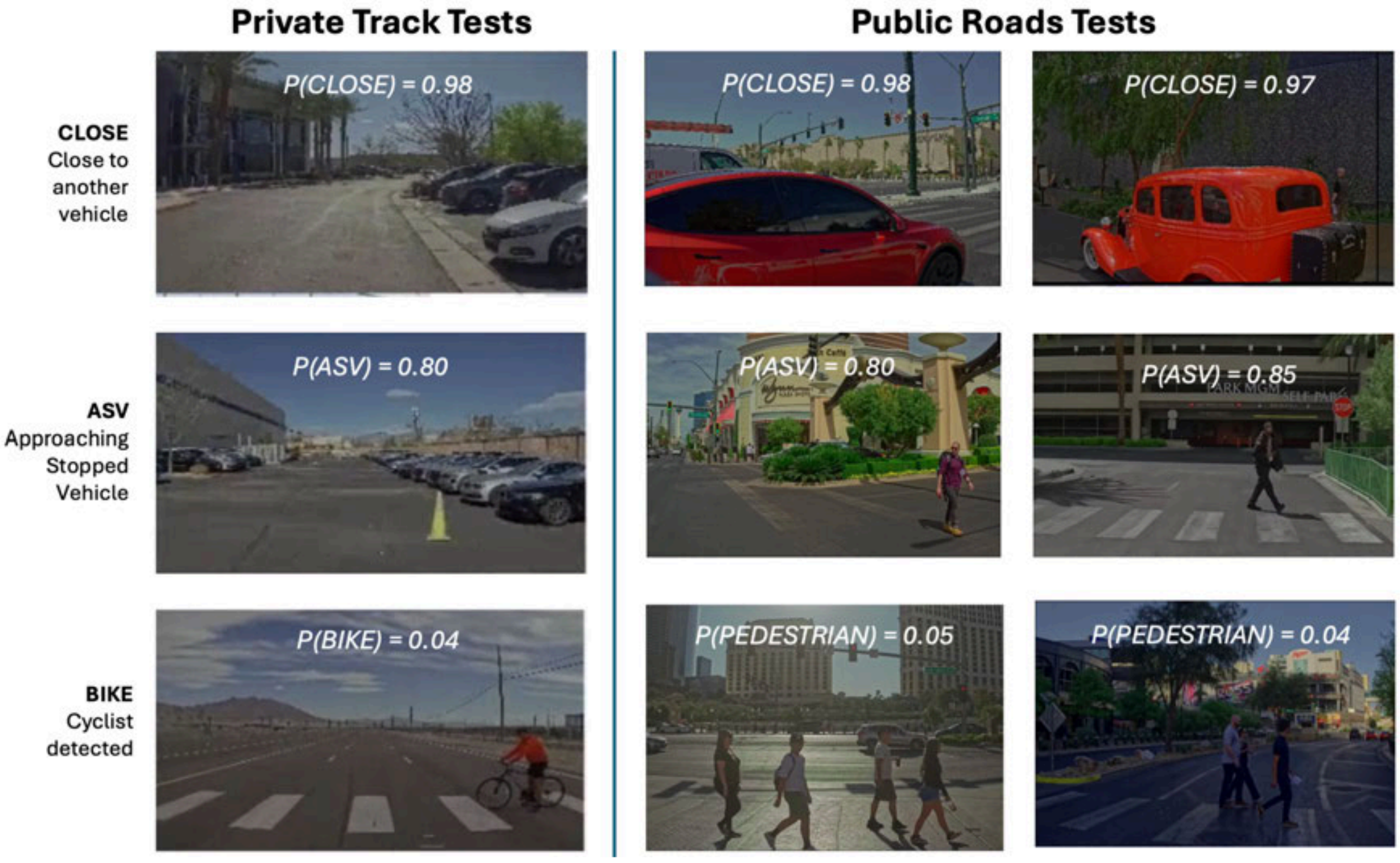

***Figure 7: Linking surprising scenarios from the private track last year, to new data on public roads**. After running our algorithm around the greater Las Vegas area for several hours, we were able to label at least two similar events to what occurred in the fully automated tests for each concept. **CLOSE**: We found two scenarios in which the AV again froze next to stationary vehicles but appeared to stop for other reasons—namely traffic lights and pedestrians in a PUDO region about to emerge behind a parked red car. **ASV**: We did not observe traffic cones on public roads, but we did find several scenarios in which the AV appeared to stop for pedestrians, while again actually stopping due to hallucination of a stopped vehicle ahead. **BIKE**: Again we did not observe cyclists on public roads, but the concept PEDESTRIAN behaved almost identically, and we found several instances where the AV (if in full self driving) would have appeared to stop for the pedestrians, but actually would have stopped due to the AV's emergency braking system, again mirroring the BIKE setup from the private-track tests.*

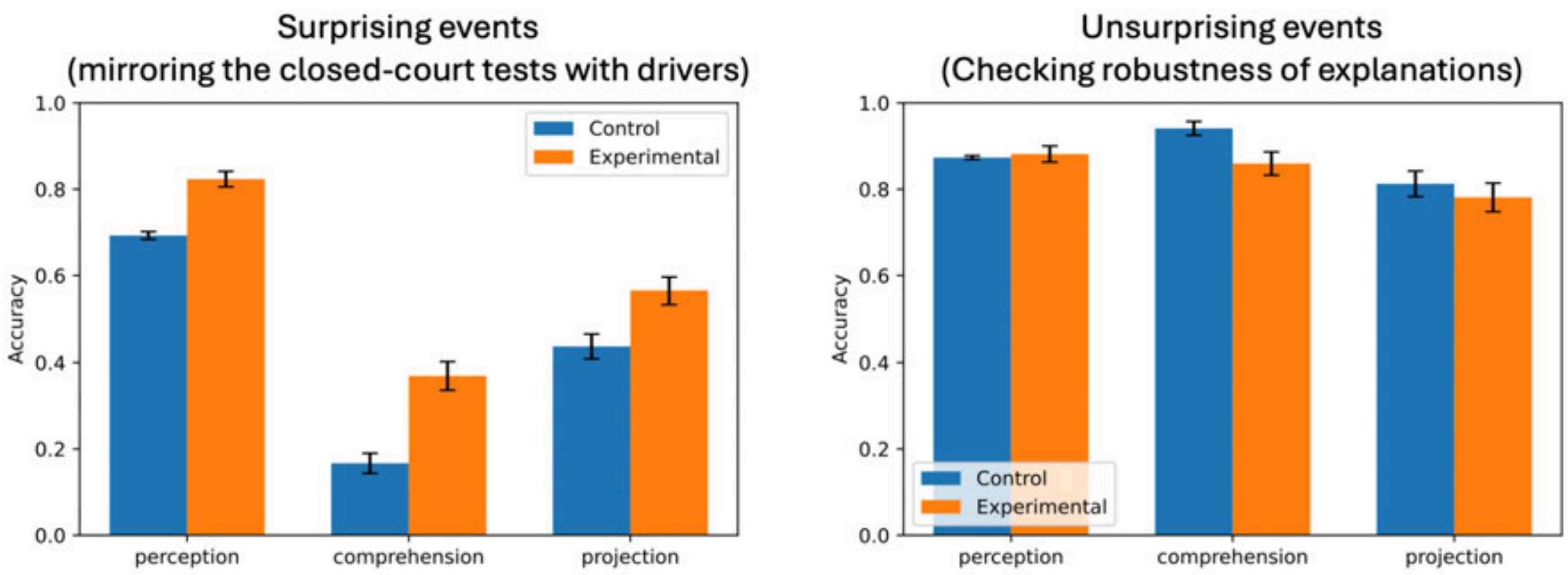

***Figure 8: SAGAT user study results with new public roads data**: The results show significant improvements in situational awareness when "surprising" events occur in all dimensions of perception, comprehension, and projection. In contrast, although comprehension trended downwards in "Unsurprising events" for the explanation group, this did not reach statistical significance after Bonferroni correction.*

Revision: We redeployed the AV on public roads to collect data for a vastly improved user study which (1) acquired more complex realistic scenarios, (2) quadrupled the size of our material set, (3) used a more validated evaluation framework SAGAT, (3) included "unsurprising" events, and (4) showed robustness of people's mental models in a wider variety of events. This updated study is in Section 6.2.

> ***Rationale for Concepts****: The proposed model CW-Net adds explainability to existing black-box planners by using interpretable concepts for humans such as "Close to another vehicle", "Approaching stopped vehicle", or "close to a bike"; however, there is no justification on how and why these concepts are selected? Obviously, these three concepts cannot cover the AV decision-making domain.*

Thanks for raising this, you are right they do not cover the entire AV decision making domain, we explain our reasoning in two main points:

3. ***Concept relevance and wider impact***: The three concepts we chose deal with other road users, and are thus most relevant for safety broadly. For example, one deployment used a dataset with concepts [`LEFT, RIGHT, STRAIGHT, STOP, SLOW, ASV, CLOSE, INTERSECTION`]. Of these, only `ASV` and `CLOSE` deal with other road users and benefit most from realtime feedback (note most—if not all—AV companies currently hardcode concepts such as 'intersection' or 'traffic light' on the map, and are thus not relevant to most companies' AI planners). The other deployment setup used concepts [`LEFT, RIGHT, STRAIGHT, STOP, SLOW, FAST, FOLLOWING, BIKE, PEDESTRIAN, INTERSECTION, TRAFFIC_LIGHT, STOP_SIGN`]. Of these, only `FOLLOWING`, `BIKE`, and `PEDESTRIAN` dealt with other road users. `FOLLOWING` was challenging to discover useful scenarios for its explanations, and required longer testing times due to the need for additional safety drivers interacting with the AV. We focused on `BIKE` , which was easier to test, although we would have observed similar results with `PEDESTRIAN` (as evidenced by the public road tests). There were no other datasets available to us at the time of testing. This largely explains why these concepts were the focus of the paper, as they are the most meaningful for AV safety broadly, and we were limited to these two datasets of concepts.
4. ***Counterfactual testing***: The concepts were also easier to test counterfactually with the drivers. For example, if the AV strongly detects `ASV`, `CLOSE`, or `BIKE`, it is expected it would drive more cautiously (or stop entirely), which allows more straightforward testing. For concepts such as `STRAIGHT` this is not the case, as the AV's speed depends upon much more than just this concept, but also obstacles on the road etc. which we may not have labels for.

As an aside, from a technical perspective, we also included these additional miscellaneous concepts (e.g. the steering and speed) because it allowed us to expand the bottleneck in our model, which is better for performance. For example, at the most extreme a CW-Net model with a single concept will have to traverse a bottleneck of a single neuron and compress the model's representations into this single neuron, which radically reduces performance, we overcame this by adding additional concepts which, although were not the focus of our tests, allowed an expanded concept bottleneck which boosted performance.

Revision: We explicitly mention this in the second paragraph of Section 5.

> *The manuscript expands the previous work of authors on PW-Net, however, no alternative models other than CW-Net are discussed or evaluated.*

The main reason for this is that there are no other notable "interpretable-by-design" methods for deep inverse reinforcement learning (IRL) which we are aware of to discuss or compare to (especially one that could take advantage of the provided labels). We noted this in the Methods section. However, in the paper we are able to compare a control condition without explanation. We believe this simple comparison also allows the impact of the work to be broader, as it indicates concept-based explanations (in general) may help driver understanding of AVs compared to the alternative, which is no explanation at all.

Revision: We mention this in the "Architecture" part of the Methods section.

> *The approach is only tested on the DriveIRL planner architecture. There is no evidence that CW-Net generalizes effectively to other planners or architectures used in AV systems.*

Thanks for bringing up this concern. There are sound theoretical performance guarantees which state PW-Net (and by extension CW-Net) will generalize to any model which makes decisions based on some latent representation of the state-trajectory pair [17]. Considering our original work [17], and the submission here, all planners used either some variant of RL or IRL. CW-Net, and its predecessor PW-Net, have empirically proven they are capable of generalizing across these two approaches, as performance of the AV has never degraded notably, even when we deployed our old algorithms last year on the updated AVs this year.

Put another way, fundamentally all AV's (based on neural nets) will have a latent representation of a state trajectory pair in some latent space before predicting a ranking, and this is the only assumption the algorithm makes to give these performance guarantees. There is no theoretical reason CW-Net would not generalize to any such setup, and the empirical evidence thus far—which is substantial—indicates this is true (see the 'Performance Guarantees' Section 3.3 of [17] and our results in the present/prior manuscripts [17]).

Revision: We mention this at the end of Section 3.

> *The proposed solution is more like an engineering setup with limited novelty.*

Although our approach of grounding the IRL concept bottleneck post-training with human defined labels is (to our knowledge) a novel approach to Explainable AI (XAI), this is not the real novelty of our work; it is instead to understand if XAI can be useful in AVs in a real-world setting, which is not previously explored to the extent we have done so here [7]. In explainable AI, this is arguably the biggest challenge the field currently faces, there are a plethora of techniques proposed over the past 10

years, but authors almost never demonstrate they are understandable and useful to intended practitioners of the system [5, 6, 7]. Indeed, Bridging the gap between theoretical XAI and operational deployment is arguably the "grand challenge" of the field. Hence, the novelty of this work is to address this gap in the context of AVs and demonstrate the usefulness of XAI. Note this is also more in line with this specific journal which has prioritized application papers with XAI (e.g., see [13]).

Revision: We have emphasized this more in the introduction's first two paragraphs.

> *Obviously, the authors have tried to link some basic human decision-making with interpretability, however, I do not believe the model can be generalizable in complex traffic environments or other domains like autonomous drones, despite the claims made at the beginning of the manuscript.*

We responded to concerns about generalization to more complex traffic environments above. In addition, we have removed speculative claims related to drones etc., they were originally included to place our work in a broader context (as the journal suggested in its guide to writing a *Nature* summary), but they are not necessary and we agree the paper is more grounded without them, thanks for this.

> *In the section conclusion, the authors might advise on how this work may influence AV regulatory standards or enhance the users' trust.*

We are extremely interested in these topics. Originally, we decided to avoid talking about these points because it seems too speculative. However, since regulation (and indeed trust) is one of the most important future considerations of AVs, as evidenced by recent survey papers [7], we have opted to include this, based on the reviewer's encouragement.

Revision: We added this discussion to the conclusion.

> *There are a couple of complex terminologies which are worth further clarifications including "causally faithful explanations", and "mental model alignment".*

We have removed any mention of "mental model alignment", instead using the term of 'mental model goodness', which is used in the literature [3]. For *"causally faithful explanations",* we mean causal in the sense of the model (not the world). As the driving decisions of CW-Net are directly made by the concept predictions, we consider them *"causally faithful explanations"*.

Revision: We added a definition for "causally faithful explanations" at the end of the introduction's third paragraph, and replaced the term "mental model alignment" with more commonly used terminology.

The structure of the article can be significantly improved. Section 8 (Methodology), the most important part of the paper, comes after the section References. Then at the end of Section 8, there is another References section. And then after the second Reference a section for Appendix. I do not like the current structure of the manuscript with two sections for references and the methodology section out of the main context. This is very unusual and makes the Manuscript hard to read/follow.

This is the standard style of this particular journal. That is, putting the methods section after references, and a second reference section before the appendix.

While the manuscript emphasises a valid concern of interpretability in autonomous vehicles and provides some references on simulation and previous works of the same authors, it suffers from an in-depth literature review about the other research works done on the interpretability of AVs. Some of the common methods such as SHAP and LIME in XAI can be reviewed as well.

We strongly believe in good scholarship, but in this journal, it is requested that authors avoid any in-depth literature review and we are (for better or for worse) limited to around 50 citations. However, we are happy to adjust the related work mentioned if the reviewer has any particular citations they feel are very relevant which we have omitted, we are very happy to include them and grateful for the suggestions to improve the work. Many thanks for your hard work here, please just let us know.

Revision: We included references to LIME and SHAP in the methods section.

The authors need to provide further evidence and more experiments to support the validity, reliability, and robustness of the model.

We are grateful for the reviewer's push for greater realism and technical depth. We have extensively revised the manuscript to provide several additional experiments for evidence of the validity of our approach, to summarise:

- **Public road deployment**: To address your concern that our initial study was limited to a “private lot,” we deployed the AV around the greater Las Vegas area. This environment provided the high-density complexity requested, involving multi-lane roads, numerous pedestrians, and complex intersections. We collected several hours of data, allowing us to capture realistic interactions in a more natural setting in a safe manner which mirrored those in the fully automated private track tests last year to draw connections.
- **Situational awareness**: We utilized this new public road data to conduct a SAGAT study (N=100), showing robustness of our results last year in more complex driving environments and in both surprising/unsurprising events.

- **Concept Selection**: We have clarified that the three focal concepts were selected because they represent the most safety-critical interactions with other road users, were the available labels at the time of testing, and easiest to test counterfactually.
- **Model Generalization**: We elaborated how CW-Net generalizes to any deep learning-based architecture, both empirically and theoretically.
- **Mental model tests**: Although not directly related to your concerns, we also conducted new mental model elicitation studies showing robustness of user mental models from our initial semi-naturalistic study with the drivers last year, to two new studies with experts/non-experts, and finally the SAGAT study (see Rebuttal Figures 1 and 2 for details), all lending credible evidence that our results and conclusions are valid.

We hope our additional evidence has addressed the reviewer's concerns adequately, many thanks for your help to improve this work.

Kind regards,

The Authors

Bibliography for reviewer responses

[1] Sanneman, L. and Shah, J.A., 2022. The situation awareness framework for explainable AI (SAFE-AI) and human factors considerations for XAI systems. *International Journal of Human–Computer Interaction*, *38*(18-20), pp.1772-1788.

[2] Endsley, M.R., 2017. Toward a theory of situation awareness in dynamic systems. In *Situational awareness* (pp. 9-42). Routledge.

[3] Hoffman, R.R., Mueller, S.T., Klein, G. and Litman, J., 2023. Measures for explainable AI: Explanation goodness, user satisfaction, mental models, curiosity, trust, and human-AI performance. *Frontiers in Computer Science*, *5*, p.1096257.

[4] Gunning, D. and Aha, D., 2019. DARPA's explainable artificial intelligence (XAI) program. *AI magazine*, *40*(2), pp.44-58.

[5] Pičulin, M., Petek, B., Ograjenšek, I. and Strumbelj, E., Position: Explainable AI Cannot Advance Without Better User Studies. In *Forty-second International Conference on Machine Learning Position Paper Track*.

[6] Freiesleben, T. and König, G., 2023, July. Dear XAI community, we need to talk! Fundamental misconceptions in current XAI research. In *World conference on explainable artificial intelligence* (pp. 48-65). Cham: Springer Nature Switzerland.

[7] Atakishiyev, S., Salameh, M., Yao, H. and Goebel, R., 2024. Explainable artificial intelligence for autonomous driving: A comprehensive overview and field guide for future research directions. *IEEE Access*.

[8] Albert, D.A. and Smilek, D., 2023. Comparing attentional disengagement between Prolific and MTurk samples. *Scientific Reports*, *13*(1), p.20574.

[9] Endsley, M.R., 2000, July. Situation models: An avenue to the modeling of mental models. In *Proceedings of the human factors and ergonomics society annual meeting* (Vol. 44, No. 1, pp. 61-64). Sage CA: Los Angeles, CA: SAGE Publications.

[10] Foster, M.I. and Keane, M.T., 2013. Surprise! You've Got Some Explaining to Do. In *Proceedings of the Annual Meeting of the Cognitive Science Society* (Vol. 35, No. 35).

[11] Mullen, N., Charlton, J., Devlin, A., & Bédard, M. (2011). Simulator validity: Behaviors observed on the simulator and on the road. In *Handbook of Driving Simulation for Engineering, Medicine, and Psychology*. CRC Press.

[12] Eriksson, A., Banks, V. A., & Stanton, N. A. (2017). Transition to manual: Comparing simulator with on-road control transitions. *Accident Analysis & Prevention*, 102, 227-234.

[13] Wong, F., Zheng, E.J., Valeri, J.A., Donghia, N.M., Anahtar, M.N., Omori, S., Li, A., Cubillos-Ruiz, A., Krishnan, A., Jin, W. and Manson, A.L., 2024. Discovery of a structural class of antibiotics with explainable deep learning. *Nature*, *626*(7997), pp.177-185.

[14] MLA (9th Edition): Crisara, Matt. "Elon Musk Promised Driverless Taxis—Instead, You Get a Guy in the Passenger Seat." *Popular Mechanics*, www.popularmechanics.com/cars/hybrid-electric/a65210102/tesla-robo-taxi-supervisor/. Accessed 11 Oct. 2025.

[15] Doshi-Velez, F. and Kim, B., 2017. Towards a rigorous science of interpretable machine learning. *arXiv preprint arXiv:1702.08608*.

[16] Islam, M., She, J., & Chagdes, J. (2024). *Assessing Pedestrian Trust in Autonomous Vehicles: a Comparative Study of Immersive Virtual Reality and Non-Immersive Video Media*. Proceedings of the ASME 2024 International Design Engineering Technical Conferences

[17] Kenny, E.M., Tucker, M. and Shah, J., 2023. Towards interpretable deep reinforcement learning with human-friendly prototypes. In The Eleventh International Conference on Learning Representations.

[18] Reagan, I.J., Cicchino, J.B., Teoh, E.R., Gershon, P., Reimer, B. and Mehler, B., 2025. Behavior change associated with using partial automation among three samples of drivers during a 4-week field trial. *Journal of Safety Research*, *94*, pp.404-414.

Referee #2

> *I do not have any further comments at this point and recommend acceptance of the manuscript for publication. Thanks for your dedication to this research.*

Many thanks for all your suggestions which have improved the quality of our manuscript.

Best wishes,
The Authors

Referee #4

We thank the reviewer for their positive comments acknowledging our study addresses the repeatedly noted '*lack of effectiveness validation in front of actual practitioners*', and provides a significant data point for the community through a successfully executed industry partnership. In the following pages, we iterate all your comments and suggestions.

> *D. Appropriate use of statistics and treatment of uncertainties*
> *1. Reporting 95% confidence intervals (CIs) alongside every effect size would make the underlying uncertainty significantly easier for readers to grasp visually and interpret.*

Revision: Agreed, we added this as Table S6 and in Section 6.2.

> *2. The "LLM-as-a-judge" component—a critical part of the current qualitative evaluation—would be considerably strengthened by releasing the specific prompts used and by conducting a simple inter-rater reliability check. Specifically, the authors should report Cohen's κ after recoding a sample (e.g., N=50) of the responses with one or two human coders to validate the LLM's assessments.*

We thank the reviewer for the excellent suggestion.

Revision: we added a new Supplemental Methods section (due to space constraints) which includes all the prompts and a new inter-rater reliability check (N=54) that validates the LLM's assessments.

3. The authors should report the internal consistency (Cronbach's α) for the four SAGAT perception items. This is necessary to statistically justify the use of a composite score for perception and ensure the robustness of the situational awareness assessment.

We thank the reviewer for raising this. Following Hultin et al. [1], we treat perception as a single latent construct measured by our 48 binary indicators (12 materials × 4 items) and compute Cronbach's alpha on the resulting 99 × 48 participant-by-item matrix. As our analysis used the aggregated composite perception score, reliability at this level is the relevant quantity. Cronbach's alpha is 0.754, above the conventional 0.70 threshold.

Gadermann et al. [2] note that, for highly skewed binary items, conventional alpha may be attenuated. This is relevant here because several of our correct/incorrect perception items were near ceiling. This applies particularly to our world-state items (Q1, Q3; "what was in the video"), where pooled mean accuracy was 0.858 for Q1, with 9/12 material-level Q1 items near ceiling (i.e., accuracy ≥ 0.85), and 0.846 for Q3, compared to the AV-state items, which were 0.786 for Q2 and 0.785 for Q4.

The AV-state items (Q2, Q4; "what the AV detected"), which are the component most directly targeted by CW-Net explanations, show strong reliability with alpha = 0.792. As a complementary reliability analysis for these binary items, we additionally compute ordinal alpha [2] (which is arguably more appropriate), giving 0.929 for the full perception scale, 0.907 for the AV-state subscale, and 0.862 for the world-state subscale. One item (an1, Q1; that is, one question out of the 48 items) was excluded from ordinal alpha because all 99 participants answered correctly.

These analyses support the reliability of the composite perception score used in our main results, we thank the reviewer for the suggestion.

Revision: We added this analysis to the Supplementary Information and Table S8/9.

E. Conclusions: robustness, validity, reliability
1. While the authors discuss the Las Vegas public road data, I recommend explicitly mentioning in the Discussion section how this approach addresses the limitations of purely synthetic or simplified simulator scenarios often found in prior situational awareness (SA) research. Emphasizing this transition from "controlled simulation" to "real-world environmental complexity" would better showcase the paper's ecological validity and its practical relevance to the AV industry.

Revision: Thanks for the suggestion, we added this as the last sentence in the Conclusion's first paragraph.

2. In the phrase "establishes a general pathway to interpretability for autonomous agents," I recommend softening "general" to "promising" or "deployment-validated" to more accurately reflect the specific context of the study.

Revision: We changed this to “deployment-validated” as you suggest.

3. The assertion "allowing them to better predict its behavior" should be qualified. Since the reported improvements are primarily observed during unexpected events, adding "particularly in surprising situations" would accurately reflect the experimental results and maintain the scientific integrity of the findings.

Revision: We added “particularly in surprising situations” to the end of this sentence.

G. References: appropriate credit to previous work?
The authors have correctly cited established XAI and HCI frameworks, including Hoffman et al. and the SAGAT methodology. To provide a more comprehensive context for the Nature readership, I suggest the following additions:
1. Distinction from Post-hoc Methods: Briefly mention the distinction between CW-Net (interpretable-by-design) and common post-hoc explanation methods such as LIME or SHAP within the Methods or Related Work section. This would clarify why an intrinsic bottleneck approach like CW-Net is particularly suited for safety-critical autonomous agents compared to additive or perturbative explanations.

Revision: Thanks for the suggestion, we have extended a sentence in our introduction with the following: *“Since the inferred concepts are the sole input to CW-Net’s final decision-making module and therefore directly determine AV behavior, we refer to them as causally faithful. Notably, our approach contrasts with popular post-hoc explanation methods [3, 4], which are applied to pretrained models and do not, by construction, guarantee faithfulness to the model’s decision process [5].*

2. Contrast with Simulator-based SA Research: I recommend citing a few benchmark studies that have historically utilized SAGAT within purely simulated environments (e.g., standard driving simulator studies found in venues like AutoUI or HFES). By explicitly referencing these simulator-based precedents, the authors can better highlight how their transition to real-world public road data addresses long-standing gaps in ecological validity. This contrast will emphasize that the current work is not merely another SAGAT application, but a significant methodological step forward in validating XAI through real-world environmental complexity.

Revision: Thanks for the suggestion, we added this to the second paragraph of Section 6.2.

> *I accessed the provided source code via the Code Ocean link to verify the reproducibility of the CW-Net implementation. While the code itself is well-organized and modular, I encountered significant technical hurdles in executing the application within the Code Ocean environment.*

Thank you for noting that our code is well-organized and modular. We are grateful that the reviewer flagged difficulties in running the Code Ocean capsule, and we regret that the execution was not as straightforward as intended. Although we could not determine the precise source of the reviewer's technical hurdles, we identified stochasticity in the training procedure as a potential cause of inconsistent convergence in the default run. We have now made the capsule fully deterministic and verified that, by simply pressing "Run," the CW-Net algorithm converges and the broader results are reproduced in our code. We thank the reviewer for prompting this improvement.

Revision: We made the Code Ocean capsule deterministic so that the default run reliably converges and reproduces the broader results in a toy domain.

We hope this has addressed the reviewer's remaining concerns.

Best wishes,

The Authors

Referee #5

> *I co-reviewed this manuscript with one of the reviewers who provided the listed reports.*

Many thanks for your help to improve the manuscript.

Best wishes,
The Authors

Bibliography for Reviewer Responses

[1] Hultin, M., Härgestam, M., Grundberg, Å., Johansson, J. and Lindkvist, M. (2019) 'Reliability of instruments that measure situation awareness, team performance and task performance in a simulation setting with medical students', *BMJ Open*

[2] Gadermann, A.M., Guhn, M. and Zumbo, B.D., 2012. Estimating ordinal reliability for Likert-type and ordinal item response data: A conceptual, empirical, and practical guide. *Practical assessment, research & evaluation*

[3] Ribeiro, M.T., Singh, S. and Guestrin, C., 2016, August. " Why should i trust you?" Explaining the predictions of any classifier. In Proceedings of the 22nd ACM SIGKDD international conference on knowledge discovery and data mining (pp. 1135-1144).

[4] Lundberg, S.M. and Lee, S.I., 2017. A unified approach to interpreting model predictions. Advances in neural information processing systems, 30.

[5] Rudin, C., 2019. Stop explaining black box machine learning models for high stakes decisions and use interpretable models instead. Nature machine intelligence, *1*(5), pp.206-215.

Response to Reviewers

Referee #4:

> *I would like to thank the authors for their exceptional diligence and thoroughness in addressing all of my previous concerns. The revised manuscript and the detailed response package demonstrate a rigorous commitment to scientific accuracy and technical completeness… I have no further comments or requests. I strongly recommend the manuscript for immediate acceptance and publication. Congratulations to the authors on this exemplary work.*

Many thanks for the kind words.

Best wishes,
The Authors

Referee #5:

> *I co-reviewed this manuscript with one of the reviewers who provided the listed reports.*

Many thanks for your assistance.

Best wishes,
The Authors
